# Differential regulation of H3K9/H3K14 acetylation by small molecules drives neuron-fate-induction of glioma cell

**DOI:** 10.1038/s41419-023-05611-8

**Published:** 2023-02-20

**Authors:** Xincheng Liu, Cui Guo, Tiandong Leng, Zhen Fan, Jialuo Mai, Jiehong Chen, Jinhai Xu, Qianyi Li, Bin Jiang, Ke Sai, Wenzhuo Yang, Jiayu Gu, Jingyi Wang, Shuxin Sun, Zhijie Chen, Yingqian Zhong, Xuanming Liang, Chaoxin Chen, Jing Cai, Yuan Lin, Jiankai Liang, Jun Hu, Guangmei Yan, Wenbo Zhu, Wei Yin

**Affiliations:** 1 Department of Pharmacology, Zhongshan School of Medicine, Sun Yat-sen University, Guangzhou, 510080 P. R. China; 2 Department of Emergency Medicine, Guangdong Cardiovascular Institute, Guangdong Provincial People’s Hospital (Guangdong Academy of Medical Sciences), Southern Medical University, Guangzhou, 510080 P. R. China; 3 Department of Neuroscience, Morehouse School of Medicine, Atlanta, GA 30310 USA; 4 Guangdong Province Key Laboratory of Brain Function and Disease, Zhongshan School of Medicine, Sun Yat-sen University, Guangzhou, 510080 P. R. China; 5 Department of Neurosurgery/Neuro-oncology, Sun Yat-sen University Cancer Center, Guangzhou, 510060 P. R. China; 6 State Key Laboratory of Oncology in South China, Collaborative Innovation Center for Cancer Medicine, Sun Yat-sen University Cancer Center, Guangzhou, 510060 P. R. China; 7 Department of Biochemistry and Molecular Biology, Zhongshan School of Medicine, Sun Yat-sen University, Guangzhou, 510080 P. R. China

**Keywords:** CNS cancer, Epigenetics

## Abstract

Differentiation therapy using small molecules is a promising strategy for improving the prognosis of glioblastoma (GBM). Histone acetylation plays an important role in cell fate determination. Nevertheless, whether histone acetylation in specific sites determines GBM cells fate remains to be explored. Through screening from a 349 small molecule-library, we identified that histone deacetylase inhibitor (HDACi) MS-275 synergized with 8-CPT-cAMP was able to transdifferentiate U87MG GBM cells into neuron-like cells, which were characterized by cell cycle arrest, rich neuron biomarkers, and typical neuron electrophysiology. Intriguingly, acetylation tags of histone 3 at lysine 9 (H3K9ac) were decreased in the promoter of multiple oncogenes and cell cycle genes, while ones of H3K9ac and histone 3 at lysine 14 (H3K14ac) were increased in the promoter of neuron-specific genes. We then compiled a list of genes controlled by H3K9ac and H3K14ac, and proved that it is a good predictive power for pathologic grading and survival prediction. Moreover, cAMP agonist combined with HDACi also induced glioma stem cells (GSCs) to differentiate into neuron-like cells through the regulation of H3K9ac/K14ac, indicating that combined induction has the potential for recurrence-preventive application. Furthermore, the combination of cAMP activator plus HDACi significantly repressed the tumor growth in a subcutaneous GSC-derived tumor model, and temozolomide cooperated with the differentiation-inducing combination to prolong the survival in an orthotopic GSC-derived tumor model. These findings highlight epigenetic reprogramming through H3K9ac and H3K14ac as a novel approach for driving neuron-fate-induction of GBM cells.

## Introduction

Glioblastoma (GBM) ranks as the most dangerous human brain tumor, which is also extremely malignant in pan-cancer analysis. At present, temozolomide and other alkylating agents are the mainstay of chemotherapy in the clinic [1]. However, due to the high undifferentiation property of glioma, even if only a few glioma cells survive from chemotherapy, radiotherapy, and surgery, they could be the root of relapse. Thus, an additional strategy transforming GBM cells into mature phenotype has high potential to improve the ultimate outcome of GBM patients with standard therapy.

Targeting histone epigenetic regulation to transform a cell´s identity has proved efficient in stem cells [2], which led to efforts aiming at histone modification to transform cancer cells and even cancer stem cells. Most research reported that histone acetylation inhibition has a uniform effect on gene regulation [3, 4]. Histone acetylation marks in Lys9 (H3K9ac) and in Lys14 (H3K14ac) are related to the activation of gene expression [5], and usually co-regulate the same genes. H3K9ac and H3K14ac have been reported to be involved in differentiation of neural stem cells to neurons [6, 7]. Although the relationship between neuron cell identity and histone acetylation regulation is over the horizon, the clinical agents for differentiation therapy through reprogramming the histone acetylation profile remain unavailable.

Differentiation-inducing therapy that can reduce the stemness of glioma cells has the potential to eradicate glioma [8]. cAMP activators such as cholera toxin, dbcAMP, and forskolin are well established as differentiation-inducers [9, 10], however, the further development of cAMP activators has been slow due to the non-lasting efficacy. In this study, we identified histone deacetylase inhibitor (HDACi) as potential effective synergist with 8-CPT-cAMP via screening in 349 compounds and unexpectedly found there exists differential regulation of H3K9ac and H3K14ac to oncogenes and differentiation-related genes.

## Results

### A synergic differentiation-inducing regimen consisting of cAMP activator and HDACi drives GBM cells into neuron-like cells

**Fig. 1:**
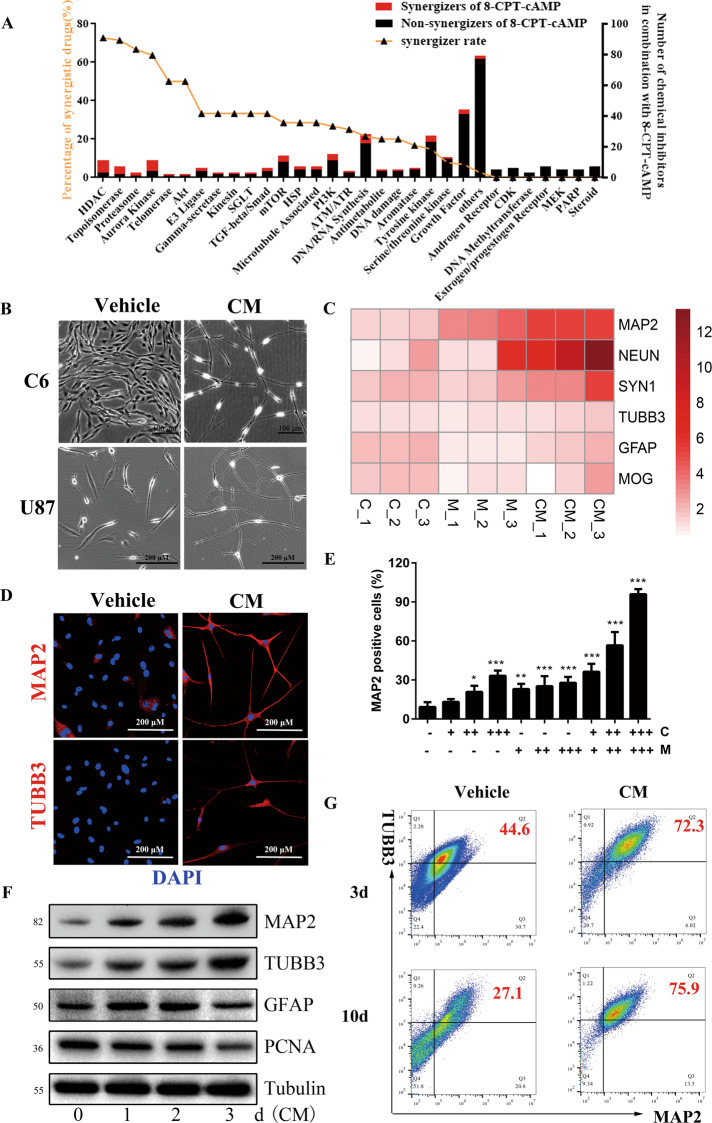
HDAC inhibitors cooperate with 8-CPT-cAMP to induce neuronal differentiation of human glioblastoma cells. **A** The percentage of drugs showing synergistic activity with 8-CPT-cAMP in agents grouped by targets. **B** Morphology alteration of rat malignant glioma C6 cells and human glioblastoma U87MG cells treated with CM for 2 days. V: Vehicle (1‰ DMSO); C: 0.5 mM 8-CPT-cAMP; M: 1 μM MS275. **C** The fold change of neuronal genes in the C, M, CM-treated group compared with the control group. **D** Immunofluorescence analysis of MAP2 (upper, red) and TUBB3 (down, red) in U87MG cells treated with CM for 2 days. The nucleus was stained with DAPI (green). **E** The percentage of MAP2-positive cells exposure to different regimens of CM. C: +0.125 mM, ++ 0.25 mM, +++ 0.5 mM; M: +0.25 μM, ++ 0.5 μM, +++ 1 μM. Data are shown as mean ± SD (*n* = 3 per group). Compared to vehicle control, one-way ANOVA with Dunnett post-test was used. *P < 0.05; **P < 0.01; ***P < 0.001. **F** Western blot analysis of MAP2/TUBB3/GFAP/PCNA in CM-treated U87MG cells for 2 days. Tubulin was used as a loading control. **G** Flow cytometry analysis of the MAP2/TUBB3 in U87MG cells treated with CM for 2 days for 3 and 10 days. The red numeric in the upper right corner represents the percentages (%) of MAP2-positive and TUBB3-positive cells in all tested cells.

In our previous study [9, 11], cAMP signal activation has been confirmed as a differentiation-inducing force in glioma cells. To maximize the differentiation-inducing activity, we tested the adjuvant differentiation-inducing activity of 349 known anti-cancer compounds in presence of cAMP analog 8-CPT-cAMP in C6 glioma cells. The result revealed that 8 of 11 tested HDACi facilitate the differentiation of glioma cells in presence of 8-CPT-cAMP (Fig. 1A, Tab S1). And other common epigenetic drugs including 5-Aza-deoxycytidine (a typical DNA methylase inhibitor) and UNC1999 (a histone methylase inhibitor) didn’t show synergic activity with 8-CPT-cAMP (Fig. S1).

Moreover, in human U87MG GBM cells and rat C6 glioma cells, CM combination consisting of 8-CPT-cAMP (C) and MS-275 (M) targeting histone deacetylases 1 and 3 (HDAC1 and HDAC3) induced marked morphologic phenotype characterized by shrinking cell bodies and long synapse-like structure (Fig. 1B). Notably, expressions of *MAP2*, *NEUN*, and *SYN1* representing neurons were upregulated by CM while expression of *GFAP* representing glia cells and *MOG* representing oligodendrocytes had no marked change (Fig. 1C), indicating the neuron fate driven by CM. Consistent with it, CM-induced cells were highly expressed with MAP2 and TUBB3 (Fig. 1D). Furthermore, a dose-dependent effect of two-agent combination on MAP2 was observed in U87MG cells (Fig. 1E). MAP2 and TUBB3 but not GFAP was increasing with time while proliferating cell nuclear antigen (PCNA) was decreasing with time (Fig. 1F), and expressions of MAP2 and TUBB3 were lasting up to 10 days (Fig. 1G). Alternative combinations, such as HDACi plus 8-CPT-cAMP, MS-275 plus forskolin, et al., were also able to induce high rate of MAP2-positive cells (Fig. S2). Taken these together, we identified cAMP activator plus HDACi as an efficient differentiation-inducing regimen driving GBM cells into neuron-like cells.

**Fig. 2:**
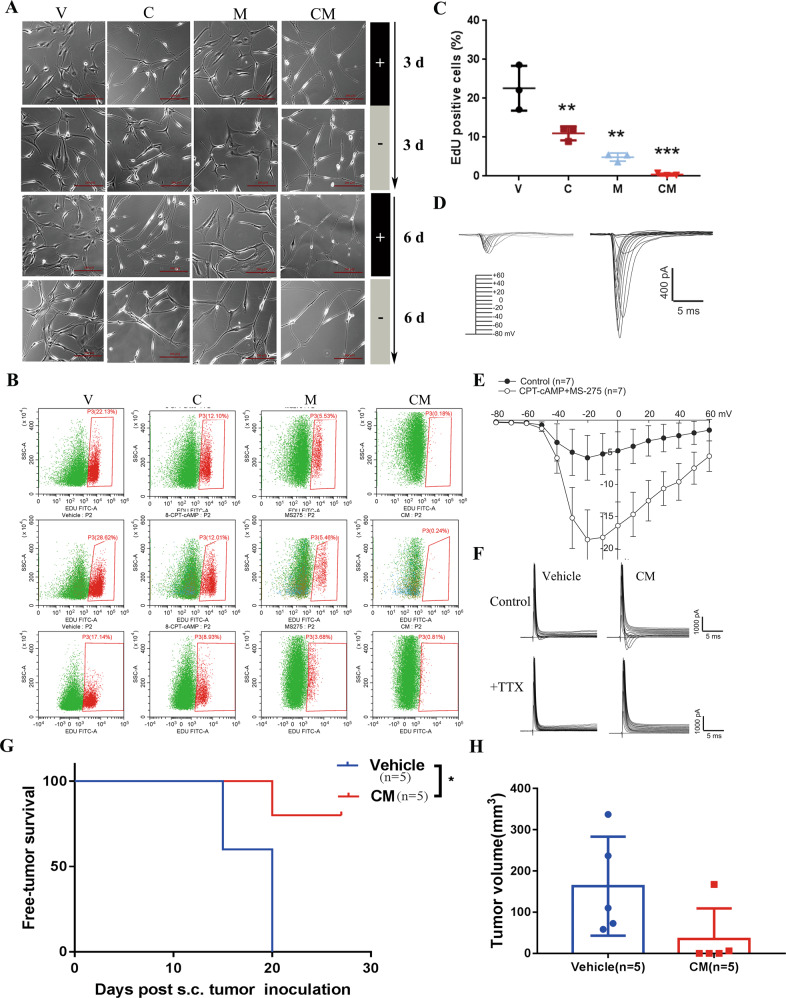
Chemical-induced neurons (CiNs) are characterized by proliferation-loss, neuron electrophysiological activity, and tumorgenicity-loss. **A** Morphology alteration of U87MG cells after drug treatment and drug withdrawal +: drug treatment; −: drug withdrawal. The cells were treated for 3 days, followed by 3 days withdrawal or treated for 6 days, followed by 6 days withdrawal. **B** Flow cytometry analysis of EdU staining (green) in U87MG cells with different combinations of CM for 72 h. **C** The percentage of EdU-positive cells in groups treated with V, C, M, CM for 3 days. Mean ± SD is shown in the bar plot (*n* = 3 per group). Compared to vehicle control, One-way ANOVA with Dunnett post-test was used. **P < 0.01; ***P < 0.001. **D** Representative trace of fast inward currents recorded in voltage-clamp mode. **E** Voltage-gated Na^+^ current amplitude of U87MG cells treated with V or CM for 2 days (*n* = 7 per group). Cells were depolarized from −80 mV to 60 mV in 10 mV increments. **F** Na^+^ currents in U87MG cells treated with V or CM for 2 days in the presence or absence of tetrodotoxin (TTX) (*n* = 7 per group). **G**, **H** Free-tumor survival of mice after inoculation of treated U87MG cells and tumor volume on the 27th day. U87MG cells were treated with V or CM for 72 h, and then 2 × 10^6^ cells were injected subcutaneously in BALB/c-nu/nu mice (*n* = 5). Tumor greater than 30 mm^3^ was considered as developed one and free-tumor survival was presented as Kaplan–Meier survival curve. The log-rank test was used for comparing V and CM. *P <0.05. V: Vehicle (1‰ DMSO); C: 0.5 mM 8-CPT-cAMP; M: 1 μM MS275.

To explore if the neuron phenotype by CM is stable, we intentionally withdrew agents 3 or 6 days post dosing and observed existing differentiated morphology (Fig. 2A). Via EdU assay, Chemical-induced neurons (CiNs) display a significant growth arrest and only 0.41% cells remain in the proliferating state (Fig. 2B, C). Furthermore, we determined whether CiNs have electrophysiological activity like mature neurons and tested the voltage-gated sodium channels (NaV) currents known to be exclusive to excitable cells. NaV current amplitudes were increased by CM (Fig. 2D, E), which was totally abolished by specific NaV blocker tetrodotoxin (TTX) (Fig. 2F).

To assess the pathogenic activity of CiNs in vivo, we inoculated tumor cells treated with vehicle or CM in nude mice and observed the tumorigenicity and tumor growth. At 20 days after inoculation, all the mice (5/5) inoculated with vehicle-treated U87MG cells developed tumors which exceeds 50 mm^3^ on the 27th day, while only one mouse (1/5) inoculated with CM-treated U87MG cells developed tumors (Fig. 2G, H). This result strongly supports that the in vivo pathogenic activity of GBM-derived CiNs was impaired pronouncedly. In all, these data further support the neuron-driving fate of GBM cells by cAMP activator and HDACi.

### Transcriptome analysis reveals down-regulation of oncogenes and upregulation of differentiation-related genes in CiNs

**Fig. 3:**
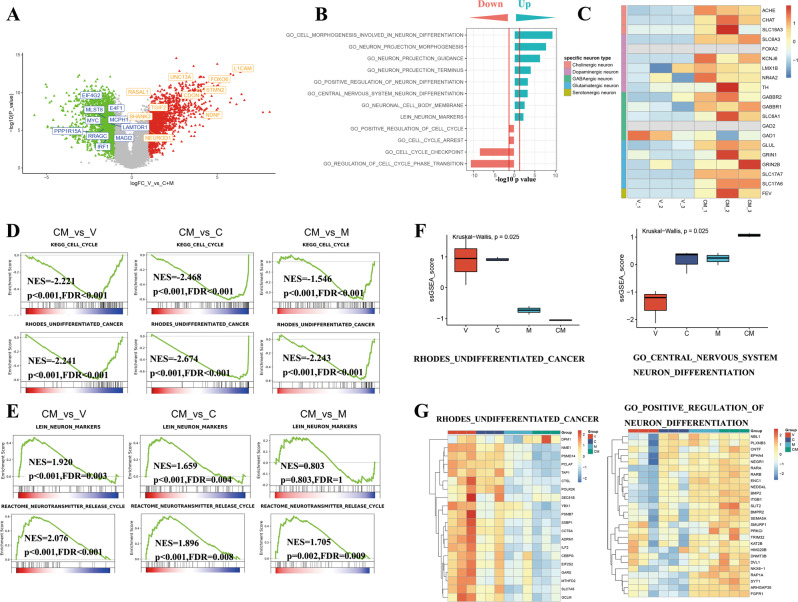
Transcriptomics analysis reveals that 8-CPT-cAMP and MS275 synergically induce GBM cells differentiation. **A** Volcano plot showing the changes in gene expression of U87MG cells treated with V or CM for 2 days. The gene symbols with orange/blue represent typical genes related to neuron-differentiation/oncogenesis. **B** Gene set enrichment analysis (GSEA) comparing V- and CM-treated U87MG cells. **C** The heatmap of specific neuron biomarkers in V- and CM-treated U87MG cells. **D**, **E** GSEA analysis of cell cycle/undifferentiation gene sets (**D**) and neuron marker/neurotransmitter gene sets (**E**). *P* < 0.05 and FDR < 0.05 were considered as significant. **F** Single sample GSEA (ssGSEA) analysis of V-, C-, M-, and CM-treated U87MG cells. Kruskal–Wallis test was used to determine the significance of differences between groups. **G** Heatmap of gene sets of undifferentiation-related genes and neuron differentiation-related genes. V, Vehicle (1‰ DMSO); C, 0.5 mM 8-CPT-cAMP; M, 1 μM MS275.

In order to further understand the gene expression profile alteration by CM, we conducted the RNA-sequencing (RNA-seq) of CiNs which is subject to transcriptome analysis. As shown in Fig. 3A, a series of neuron-related genes such as NEUROD1, NDNF, and oncogenes such as MYC, MLST8 were respectively upregulated or downregulated by CM. The upregulated genes accumulate in gene sets of neuron cell morphogenesis, projection morphogenesis, projection guidance, and projection terminus, while the downregulated genes accumulate in gene sets of cell cycle regulation (Fig. 3B). The heatmap of classical biomarkers shows a broad upward expression of neurotransmitter-related genes for CiNs, including cholinergic, dopaminergic, GABAergic, glutamatergic and serotonergic neuron markers (Fig. 3C), indicating no specific subtype of neuron fate driven by CM. The further analysis revealed that either 8-CPT-cAMP or MS-275 alone induced the downregulated of cell cycle-related gene and inhibited cell proliferation significantly (Fig. 2B, C), in which the role of MS275 is stronger than that of 8-CPT-cAMP (Fig. 3D, F, G). When it comes to neuron differentiation-related gene, both of 8-CPT-cAMP and MS-275 contributed to the increase of this gene cluster (Fig. 3E–G). These results indicate that CiNs are characterized with a neuron-like transcriptome profile which is attributed to synergic effects of 8-CPT-cAMP and MS-275.

### H3K9ac and H3K14ac induced by CM differentially regulated oncogene and neuron differentiation-related genes

**Fig. 4:**
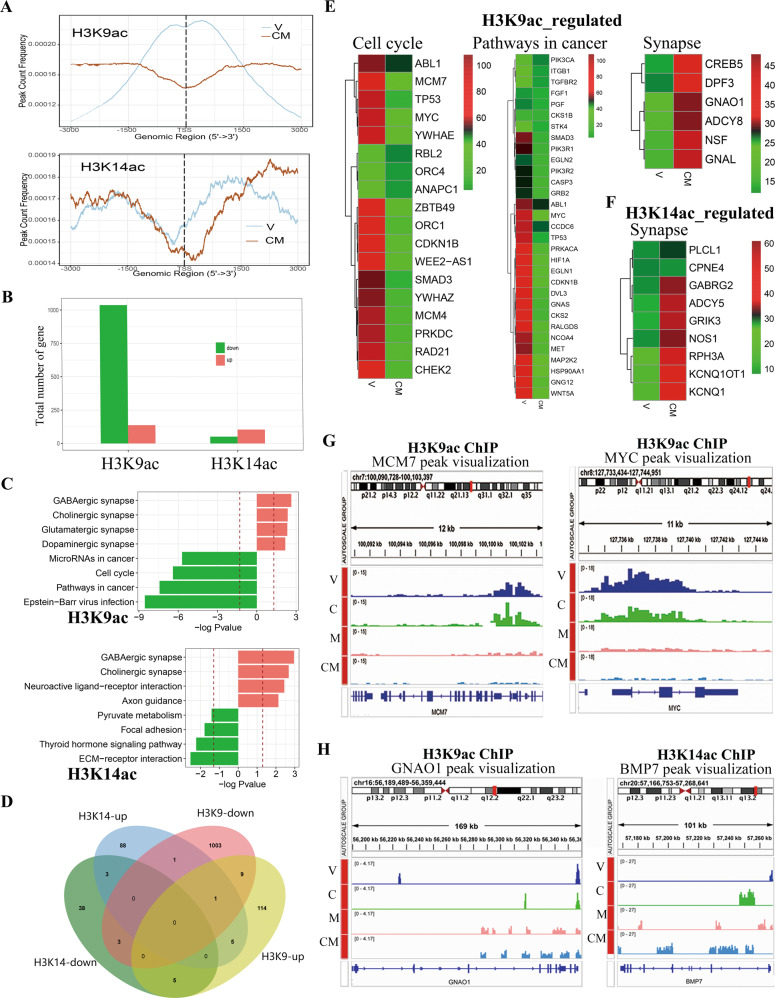
ChIP-seq analysis shows the effect of CM on H3K9ac/H3K14ac-marked genes. **A** Average profile of H3K9ac/H3K14ac peaks binding to TSS region in U87MG cells treated with V or CM for 2 days. **B** Dysregulated genes with H3K9ac/H3K14ac tags identified by ChIP-seq. **C** Gene enrichment analysis of genes with altered H3K9ac/H3K14ac tags. Red trace lines indicate adjusted *P* = 0.05. **D** Venn diagrams showing gene counts regulated by H3K9ac and/or H3K14ac. **E**, **F** Heatmap of genes with altered H3K9ac/H3K14ac tags. **G**, **H** Representative peak plot of oncogenes regulated by H3K9ac (MCM1 and MYC). **H** Representative peak plot of neuron development-related genes regulated by H3K9ac/H3K14ac (GNAQ1 and BMP7). V, Vehicle (1‰ DMSO); C, 0.5 mM 8-CPT-cAMP; M, 1 μM MS275.

Given that HDAC1 and HDAC3 targeted by MS-275 mainly account for the deacetylation of histone subunits 3 (H3), especially on histone H3 lysine9 (H3K9), H3 lysine 14 (H3K14), and H3 lysine 27 (H3K27) [12–14], we sought to clarify if the acetylation of H3K9, H3K14, and H3K27 (H3K9ac, H3K14ac, and H3K27ac) involve in changed gene profile driving differentiation form cancer cells to neuron-like cells. Through Chromatin immunoprecipitation-sequencing (ChIP-seq) assay, we surprisingly found that tags of H3K9ac and H3K27ac on the promoter intensely decreased by 13.1% and 6.8%, respectively, while one of H3K14ac on the promoter increase by 1.2% (Fig. 4A, S3A–C). In details, for H3K9ac, 137 genes with increased H3K9ac tags cluster in synapse, while 1037 genes with decreased H3K9ac tags cluster in cell cycle regulation and pathways in cancer (Fig. 4B, C). For H3K14ac, 104 genes with increased H3K14ac tags also cluster in synapse, while 51 genes with decreased H3K14ac tags cluster in metabolism and adhesion-related pathway (Fig. 4B, C). For H3K27ac, 3145 genes with increased H3K27ac tags cluster in predominantly adhesion-related pathways, while there’s no enriched pathways in 1125 genes with decreased H3K27ac tags (Fig. S3D, E). These data suggest that H3K9ac and H3K14ac but not H3K27ac made major contributions to differentiation-induction.

To further characterize the alteration of H3K9ac and H3K14ac, we tested that the protein levels of H3K9ac and H3K14ac increase with MAP2 and TUBB3 and highly co-locate in nucleus of CM-treated cells (Fig. S4). Intriguingly, there’s little overlap between genes controlled by H3K9ac and H3K14ac (Fig. 4D). As shown in Fig. 4E, F, genes in cell cycle- and cancer-related pathways were weakly marked by H3K9ac in CM group, and genes in synapse-related pathway were strongly marked by H3K9ac/K14ac.

To clarify the molecular mechanism underlying the synergy between HDACi and cAMP agonist, we analyzed the respective contribution of two agents to gene regulation by H3K9ac and H3K14ac via focusing on several representative genes. Our analyses showed that the mark alterations of H3K9ac on the promotor region of oncogenes (MCM7 and MYC) and differentiation-related gene (GNAO1) were predominantly brought by MS275 (Fig. 4G, H). The increase in tags of H3K14ac on the promotor region of differentiation-related gene BMP7 were attributed to both of 8-CPT-cAMP and MS275 (Fig. 4H).

Thus, all these data suggest that 8-CPT-cAMP and MS275 synergistically regulate the acetylation of H3K9 and H3K14 which differentially control the expressions of oncogenes and neuron differentiation-related genes.

### H3K9ac and H3K14ac regulated genes are strongly associated with glioma grade and survival

**Fig. 5:**
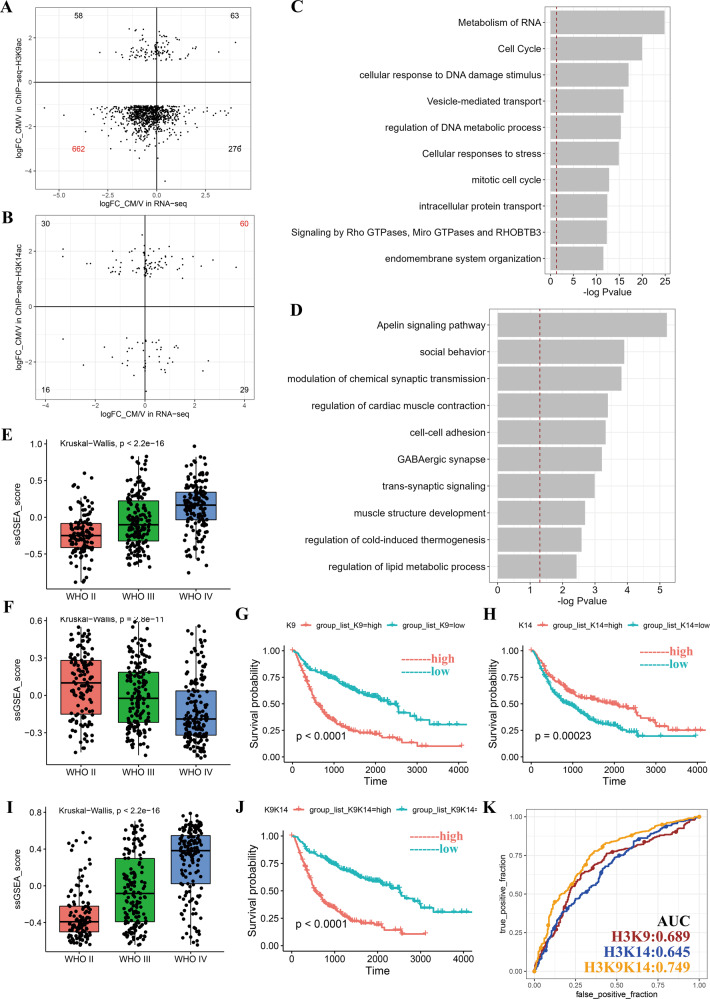
Integrated analysis reveals the diagnostic value of genes regulated by H3K9ac and H3K14ac in clinic. **A**, **B** Integrated analysis of RNA-seq and ChIP-seq data. The genes with indicated fold change (>1 or <−1, CM vs V) in the ChIP-seq are plotted. The x-axis represents fold changes of genes in RNA-seq analysis and the y-axis represents fold changes of genes in ChIP-seq analysis. The numbers represent gene counts. **C**, **D** Gene enrichment analysis of H3K9ac-associated 662 downregulated genes (**C**) and H3K14Ac-associated 60 upregulated genes (**D**). Red trace lines indicate adjusted *P* = 0.05. **E**, **F**, **I** ssGSEA score of H3K9ac- (**E**), H3K14ac- (**F**), and H3K9/K14ac-regulated genes (**I**) in WHO II/III/IV patients from Chinese Glioma Genome Atlas (CGGA), calculated by GSVA. Kruskal–Wallis test was used. **G**, **H**, **J** Kaplan–Meier curves of the patients in CGGA, which were grouped into high or low group according to their ssGSEA score of H3K9ac- (**G**), H3K14ac- (**H**), and H3K9/K14ac-regulated genes (**J**). Kruskal–Wallis test were used. **K** Predictive power of the ssGSEA score of H3K9, H3K14 and H3K9/H3K14 for clinical grade of glioma, which was classified into lower-grade glioma (LGG, WHO II/III) or GBM (WHO IV), using the receiver operation characteristic (ROC) curve. AUC is area under the curve.

Through integrated analysis, we identified 662 genes marked by H3K9ac were consistently downregulated and 60 genes marked by H3K14ac were consistently upregulated in both of RNA-seq data and ChIP-seq data (Fig. 5A, B, and Tab S2). Functional annotation showed 662 downregulated genes were mainly clustered in cell cycle and protein transport pathways, while 60 upregulated genes were clearly associated synapse formation and transmission (Fig. 5C, D). Through mining the transcriptomic data from 693 GBM patients from the Chinese Glioma Genome Atlas (CGGA) [15, 16], we found that a high single sample GSEA score of H3K9ac were strongly related with high WHO grade and low survival probability, while the opposite was observed for H3K14ac (Fig. 5E–H). Furthermore, we defined an integrated score for all 693 patients by simply using the score of H3K9ac minus one of H3K14ac, which we called the H3K9/K14 score. As expected, the integrated score increased along with the severity of the disease (Fig. 5I, J and Tab S3), and has a higher predictive value for classifying the WHO grade of glioma than the individual scores (Fig. 5K).

### Neuron-like fate induction of GSC by CM is also attributed to H3K9ac and H3K14ac

**Fig. 6:**
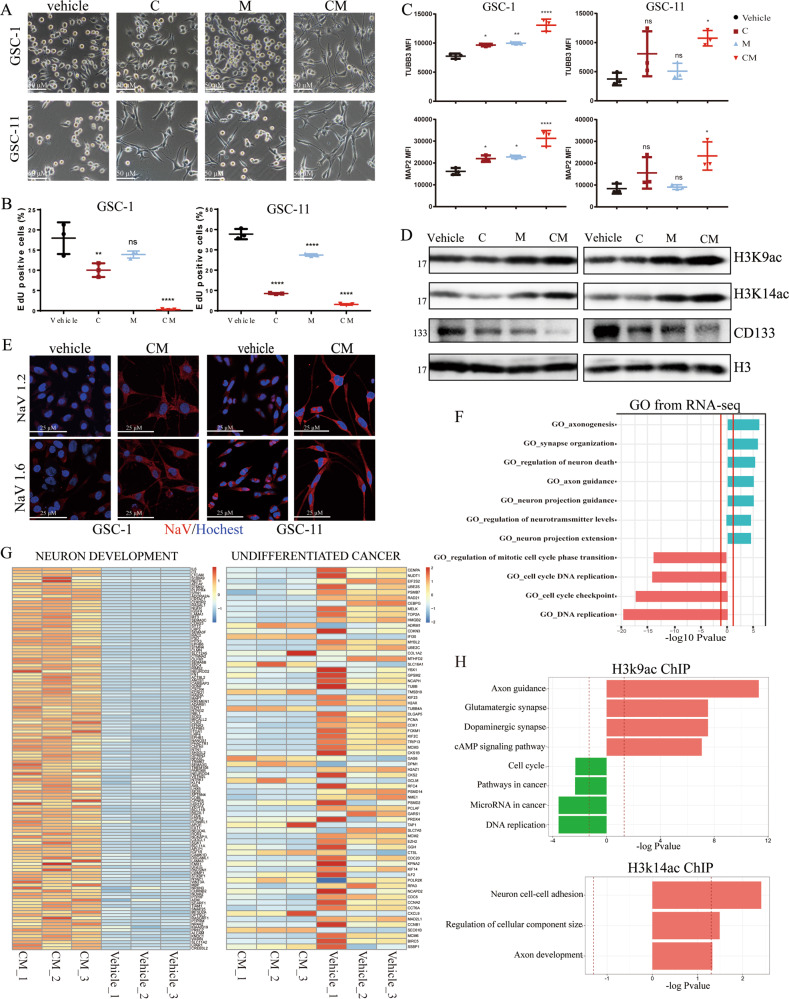
HDAC inhibitors cooperate with 8-CPT-cAMP to induce neuronal differentiation of human glioma stem cells (GSCs). **A** Morphology alteration of human GSCs (GSC-1 and GSC-11 cells) treated with V, C, M, CM for 48 h. Pictures were taken at ×20 magnification. Scale bars, 50 μm. **B** Quantification of EdU-positive cells in GSC-1 and GSC-11 treated with V, C, M, CM for 48 h. **C** Flow cytometry analysis for testing the protein levels of MAP2/TUBB3 in GSC-1 and GSC-11 cells treated with V, C, M, CM for 48 h. **D** Western blot analysis for testing H3K9/K14ac and CD133 expression in GSC-1 (left) and GSC-11 cells (right) treated with V, C, M, CM for 48 h. **E** Immunofluorescent analysis for voltage-gated sodium channels (NaV) in GSC-1 and GSC-11 treated with CM for 72 h. Pictures were taken at ×40 magnification. Scale bars, 25 μm. **F**, **G** GO analysis after RNA-seq and heatmaps of dysregulated genes for GSC-11cells treated with V and CM for 48 h. Blue: upregulated genes (CM vs V), red: downregulated genes (CM vs V). Red lines indicate adjusted *P* = 0.05. **H** Gene enrichment analysis of genes with altered H3K9ac/H3K14ac tags after ChIP-seq in GSC-11 cells treated with V and CM for 48 h. Red trace lines indicate adjusted *P* = 0.05. All data was compared to vehicle with one-way ANOVA. n.s., not significant; *P < 0.05; **P < 0.01; ****P < 0.0001. Mean ± SD is shown in the bar plot (*n* = 3 per group). V, Vehicle (1‰ DMSO); C, 0.5 mM 8-CPT-cAMP; M, 1 μM MS275.

To clarify if CM combination has the same differentiation-inducing effect on glioma stem cells (GSCs) which are recurrence seeds of glioma, we evaluated the phenotype change, the transcriptome profile, and the profile of genes marked by H3K9ac and H3K14ac. First, we used two GSCs lines GSC1 and GSC11, both of which have been systematically validated to be characterized with stem cell properties [17–20]. Consistent with data from U87MG cells, CM-treated GSC cells were characterized by shrinking cell bodies and long synapse-like structure (Fig. 6A), reduced ratio of EdU-positive cells (Fig. 6B), augmented expression of TUBB3 and MAP2 (Fig. 6C), downregulated express of stem cell marker CD133 (Fig. 6D), and increased signal of voltage-gated sodium channels NaV 1.2 and NaV 1.6 (Fig. 6E), accompanied with upregulated protein levels of H3K9ac/K14ac (Fig. 6D). Transcriptome analysis revealed that the upregulated genes clustered in a subset of neuron development and downregulated genes clustered in a subset of cell cycle regulation, further supporting the judgment of neuron-like phenotype transformation (Fig. 6F, G). Furthermore, via ChIP-seq analysis, we also observed the decrease in H3K9ac tags of cell-proliferation genes and the increase in H3K9ac/H3K14ac tags of the neuron-related genes in GSC-11 cells (Fig. 6H), in agreement with U87MG. In addition, H3K27ac also exhibits very little contribution to the neuron transdifferentiation of GSC-11 (Fig S3F). On the whole, cAMP agonist combined with HDACi also induced GSCs to differentiate into neuron-like cells through the regulation of H3K9ac/K14ac, indicating that combined induction has the potential for recurrence-preventive application.

### cAMP activator and HDAC inhibitor cooperate with temozolomide to improve the tumor growth and survival in glioma stem cell-derived GBM models

**Fig. 7:**
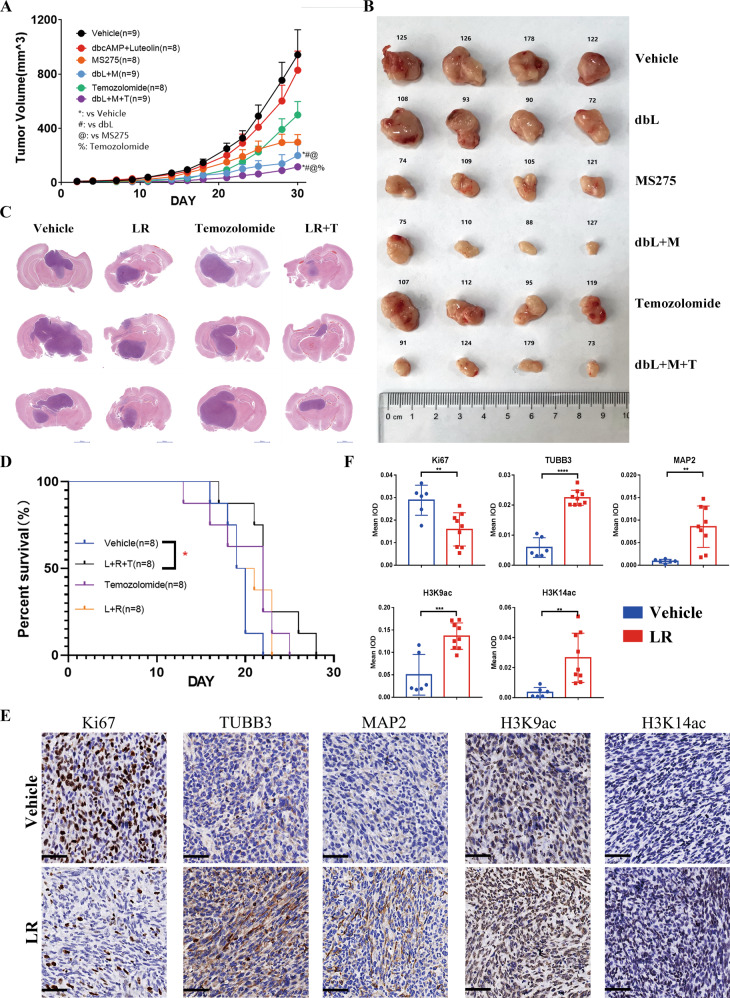
The pharmacologic activity of small molecules in vivo. **A**, **B** Tumor-growth curve and representative xenografts. Nu/nu mice were subcutaneously inoculated with GSC-1 (2 × 10^5^ cells). Seven days after inoculation, mice were intraperitoneally treated with vehicle (V, 20% HB-β-CD), dbcAMP (db, 20 mg/kg), luteolin (L, 40 mg/kg), MS275 (M, 20 mg/kg), temozolomide (T, 100 mg/kg), two-agent combination or three-agent combination. Tumor volumes were recorded every two days until the biggest tumor volume reached 1500 mm^3^. Mean ± SD is shown in the tumor-growth curve (**A**). *P*-value was determined by one-way ANOVA (*P < 0.05). The top four largest tumors in each group were shown in (**B**), in which the value above the tumor represents animal number. **C**, **D** HE staining of brain slices (**C**) and Kaplan–Meier survival curve (**D**). Nu/nu mice were orthotopically inoculated with GSC-1. The mice were intraperitoneally treated with vehicle (V, 20% HB-β-CD), luteolin (L, 20 mg/kg) and RGFP109 (R, 50 mg/kg), temozolomide (T, 50 mg/kg), two-agent combination or three-agent combination. *P*-value was determined with the log-rank test (*P < 0.05). **E**, **F** Immunohistochemistry analysis of ki-67, TUBB3, MAP2, H3K9ac, and H3K27ac signal in brain slices. Representative images of immunohistochemistry were shown in E. Immunohistochemistry pictures taken at ×20 magnification. Scale bars, 50 μm. Quantification of Integrated optical density (IOD) was shown in (**F**) (means ± SD, unpaired t test). One point represents an optical microscope field, and three fields in every animal were collected for counting and statistical analysis. **P < 0.01; ***P < 0.001; ****P < 0.0001.

To illustrate the transdifferentiation efficacy of CM combination in vivo, we evaluated the tumor growth, differentiation markers, and H3K9/K14ac levels in subcutaneous and orthotopic GBM models. In subcutaneous xenografts derived from GSC-1, we used dbcAMP in combination with luteolin which targets phosphodiesterases and thus can increase the accumulation of dbcAMP in vivo via inhibiting dbcAMP metabolism. As shown as Fig. 7A, B, the three-agent (dbcAMP, luteolin plus MS275) combination group reached a better tumor-inhibition than the two-agent group (dbcAMP plus luteolin). In this model, the addition of temozolomide didn’t further potentiate this effect based on three-agent treatment. In the orthotopic model derived from GSC-1 cells, we used the cAMP agonist luteolin mentioned above and another HDAC1/3 inhibitor RGFP-109, both of which can readily cross the blood-brain barrier [21–23]. Before the administration, we confirmed the transdifferentiation activity of luteolin plus RGFP-109 (LR) in vitro. In agreement with data from CM combination in U87MG cells, LR treatment suppressed cell proliferation (Fig. S5A, B), augmented the expression of neuron marker protein TUBB3 and MAP2 as well as the levels of H3K9ac/K14ac (Fig. S5C–E). In vivo, significant smaller tumor mass and longer survival was observed in the three-agent (luteolin, RGFP109, and temozolomide) group than in two-agent (luteolin plus RGFP109) group or single-agent (temozolomide) group (Fig. 7C, D). Moreover, we also observed MAP2/TUBB3 and H3K9ac/K14ac were markedly upregulated by LR combination, while signal of Ki67 indicating the proliferative state was downregulated (Fig. 7E, F). Apparently, in vivo, glioma cells exhibit a proliferation-loss phenotype and a neuronal differentiation phenotype in the presence of differentiation-inducing agents.

## Discussion

**Fig. 8:**
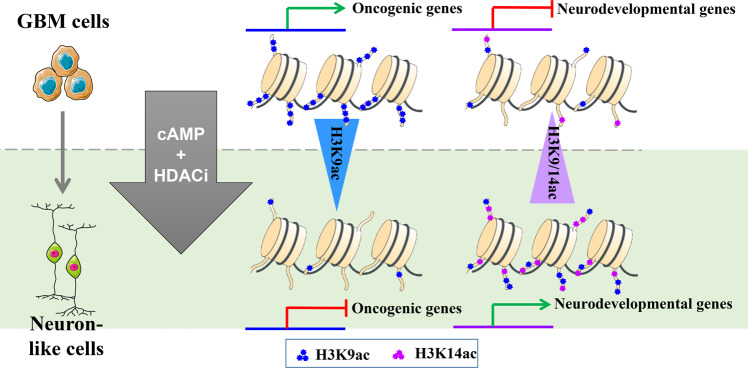
Illustration of a model on differential regulation of H3K9/H3K14 acetylation reshaping glioma cells into neuron-like cells. In Brief, this study shows that glioblastoma cells can be differentiated into neurons by a combination of cAMP activator and HDAC inhibitor. Mechanistically, ChIP-seq and RNA-seq illustrate that the drug combination can specifically decrease the level of H3K9ac in oncogenes and increase the level of H3K9ac and H3K14ac in differentiation genes to reshape glioma cells into neuron-like cells.

Our results identify HDAC inhibitors as excellent synergists of cAMP activators in reshaping glioma cells into neuron-like cells. Intriguingly, although inhibiting the deacetylation activity of HDAC1 and HDAC3 caused increasing histone acetylation levels of H3K9 and H3K14, not the global gene expression was upregulated. In detail, oncogenes were downregulated in result of decreased H3K9ac tags in the promoter region, while differentiation-related genes were upregulated in result of both of H3K9ac and H3K14ac. Intriguingly, the gene group regulated by H3K9ac and H3K14ac have a good predictive potential for WHO grade and prognosis. This study illustrates that specific histone acetylation changes could alter the cell´s identity by shifting the gene expression balance from oncogene-active to differentiation-active (Fig. 8).

Differentiation can be realized by transcription factor-mediated reprogramming and compounds-mediated reprogramming. Small molecular compounds have unique advantages in the field of differentiation therapy for tumor. Firstly, small molecules avoid importing viral genome into cells, which were usually used as the vector in transcription factor-mediated reprogramming [24, 25]. Secondly, small molecular compounds were conducive to the development of large-scale reproducible scientific research for their defined targets, easy availability and high selectivity. Finally, small molecular compounds are relatively cheap and can save costs in clinical use [26]. Tumor differentiation therapy via small molecular compounds such as all-trans retinoic acid and/or arsenic trioxide have improved the five-year survival rate of the M3 type acute promyelocytic leukemia patients from 10% to 90% strongly implying the potential of small molecule in tumor differentiation therapy [27]. In our study, to the best of our knowledge, we identified a small molecule combination driving GBM cells into neuron-like cells via epigenetic modification.

In studies focusing on strategy targeting epigenetic regulatory, there was widespread concern that HDAC inhibitors could widely increase the expression of genes. Here we demonstrated a site-specific histone acetylation pattern driven by small molecules in malignant glioma, constituting the “histone code” leading to transcriptional activation of neuron differentiation-related genes and silencing of oncogenes including cell cycle regulation genes. This histone code consists of H3K9ac and H3K14ac, two epigenetic marks representing transcriptionally active chromatin, which offers the opportunity to purposefully drive the directional differentiation of glioma cells into neurons. H3K9ac and H3K14ac regulated by HDAC1/3 have been reported to be involved in differentiation of neural stem cells to neurons [7, 28]. Consistent with this, embryonic development analysis has revealed HDAC1 and HDAC3 dynamically regulating H3K9ac as key players for neurogenesis [6]. Another independent study reported that H3K9/K14ac are involved in the neural commitment from pluripotent embryonic stem cells (ESCs) [29]. These data indicate that embryonic neurogenesis and GBM differentiation may share similar epigenetic mechanism mediated by H3K9ac and H3K14ac.

H3K9ac and H3K14ac, as pivotal epigenetic marks of transcriptionally active chromatin, were reported to show very high correlation between each other suggesting a coordinated regulation of active histone marks in mouse embryonic stem cells [30]. In our study, we also observed the highly co-location in nucleus of CM-treated cells (Fig. S4B, C). However, genes marked by H3K9ac and H3K14ac have little overlap (Fig. 4D), supporting the independent role of these two acetylated sites in gene expression regulation. In addition, in the same report, H3K9ac and H3K14ac mark active enhancers along with H3K27ac [30]. Herein, in neuron-fate-induction driven by small molecules, we also observed a remodeling of H3K27ac tags occurs in thousands of genes. However, only very a few of genes cluster in axon guidance and majority of dysregulated genes has nothing to deal with neurogenesis, indicating weaker regulatory effect of H3K27ac on neuron-fate-induction than originally thought.

Compared with neuron development genes controlled by H3K9ac/K14ac, cell cycle-related genes were predominantly regulated by H3K9ac alone (Fig. 4G), keeping in consistent with the study showing H3K9ac transcriptionally targets carcinoma-related genes including cell cycle-regulated genes and activates their expression to promote tumor formation in liver cancer [31]. A significant reduction of H3K9ac tags on cell cycle gene in the presence of HDAC1/3 inhibition is clear and provides a good explanation for the almost complete inhibition of proliferation of glioma cells and GSC cells. However, what underlies the opposite change of H3K9ac tags marking on neuronal differentiation gene and cell cycle-related gene is largely unknown and needs a further exploration.

In this study, transcriptome data uncovered that HDAC1/3 inhibitor MS-275 accounts for the repression of cell proliferation genes, whereas cAMP agonist and MS275 cooperates with each other to promote the expression of neuronal differentiation genes (Fig. 3D–G). Correspondingly, ChIP-seq analysis showed that MS275 is responsible for the regulation of H3K9ac to cell proliferation and neuronal differentiation genes while cAMP agonist and MS275 contributes to the regulation of H3K14ac to neuronal differentiation genes (Fig. 4G, H). MS275 as HDAC1/3 inhibitor has a general acetylation-promoting effect to H3K9/H3K14, in line with the knowledge that HDAC1/3 underlie H3K9 acetylation and HDAC3 underlies H3K14 acetylation [6, 32]. cAMP agonists primarily mediate the regulation of H3K14ac, which may be attributed to PKA/p300/CBP the downstream of cAMP. It’s known that histone acetylation is evenly controlled by histone acetylases (HATs) and HDACs. Intriguingly, acetylation of H3K9 and H3K14 is regulated by different members of HATs family. Specifically, GCN5/PCAF and/or Tip60 is involved in H3K9 acetylation, whereas H3K14 acetylation is performed by GCN5/PCAF, p300/CBP, and/or Myst3 [30]. There’s an evidence supporting that neuronal differentiation requires CBP/P300-mediated acetylation of H3K14 [7], thus we could reasonably deduce that cAMP agonist facillitates the acetylation of H3K14 through CBP/P300. By different mechanism, MS275 and 8-CPT-cAMP synergically reprogram the profile of H3K9ac and H3K14ac, which drive the specific cell fate differentiation of glioma cells and GSCs.

Overall, this study demonstrates that combination of cAMP activator and HDAC inhibitor is able to precisely drive the cell fate of GBM cells to neurons. Moreover, the differential regulation mode of H3K9ac and H3K14ac on oncogenes and differentiation-related genes provide a new view to see how specific acetylation sites exactly regulate embryogenesis, carcinogenesis, and cell fate. Meaningfully, the gene profile regulated by the acetylation of H3K9 and H3K14 can be used as evaluating differentiation level and predicting the prognosis after therapy in the future differentiation therapy for GBM.

## Materials and methods

### Cell culture

Human GBM U87MG cells and rat glioma C6 cells were purchased from the American Type Culture Collection and cultured in DMEM supplemented with 10% fetal bovine serum (Gibco, USA).

GSC1 and GSC11 cell lines were derived from human GBM tumor samples and were characterized as reported previously [17–20]. Briefly, freshly resected GBM tissues were cut into small pieces, washed three times in DMEM/F12 medium (Gibco, Carlsbad, CA, USA), digested with trypsin (Gibco), triturated mechanically with surgical scissors, and filtered through a cell strainer (BD Biosciences, Franklin Lakes, NJ, USA). The erythrocytes were removed with a red cell lysis buffer (Tiangen, Beijing, China). Tumor cells were then washed repeatedly with DMEM/F12 medium and were cultured in serum-free DMEM/F12 medium supplemented with 2% B27 (Gibco), 20 ng/mL basic fibroblast growth factor (bFGF, Gibco), and 10 ng/mL epidermal growth factor (EGF, Gibco). These two cell lines were maintained in a humidified atmosphere at 37 °C under 5% CO_2_. GSC-spheres were dissociated by using StemPro Accutase Cell Dissociation Reagent (Gibco) for serial passaging or further experiments. For differentiation-inducing assay, we pre-coated 6-well plates with poly-L-Lysine (PLL) before seeding cells.

### Drug screening

C6 cells were treated with 0.5 mM 8-CPT-cAMP (Enzo life sciences, USA) in the presence or absence of one agent in 349 drugs (Selleck, USA) [33], and then subject to morphologic evaluation. Exposure to different combinations, when cells are characterized by neuron-like morphology, the agent in combination of 8-CPT-cAMP is judged as a differentiation-inducing synergist.

### qRT-PCR

Total RNA was extracted using TRIzol (Life Technologies, USA) reagent and reverse-transcribed to cDNA using oligo(dT). Specific gene expression was quantified with SuperReal PreMix SYBR Green (TIANGEN, China) using an Applied Biosystems 7500 fast real-time PCR system (Life Technologies, USA). The sequences of primers used are as follows:

*MAP2*, sense, GGGCCTTTTCTTTGAAATCTAGTTT;

*MAP2*, antisense, CAAA TGTGGCTCTCTGAAGAACA;

*NEUN*, sense, CCCATCCCGACTTACGGAG;

*NEUN*, antisense, GCTGAGCGTATCTGTAGGCT;

*SYN1*, sense, AGTTCTTCGGAATGGGGTGAA;

*SYN1*, antisense, CAAACTGCGGTAGTCTCCGTT;

*TUBB3*, sense, GGCCAAGGGTCACTACACG;

*TUBB3*, antisense, GCAGTCGCAGTTTTCACACTC;

*GFAP*, sense, ACATCGAGATCGCCACCTACA;

*GFAP*, antisense, GTCTGCACGGGAATGGTGAT;

*MOG*, sense, AGAACGCTACAGGCATGGAG;

*MOG*, antisense, CAGGGCTCACCCAGTAGAAAG.

### Western blotting

The cells were lysed using M-PER mammalian protein extraction reagent (Thermo Scientific, USA), followed by SDS-PAGE. After being electroblotted onto a polyvinylidene fluoride membrane (Roche, USA), target proteins were detected with corresponding antibodies. The following antibodies were used: *MAP2* (Cell Signaling Technology), *TUBB3* Cell Signaling Technology), H3 (Cell Signaling Technology), H2Ac (Millipore, USA), H3K9ac (Cell Signaling Technology), H3K14ac (Cell Signaling Technology, USA), *PCNA* (Cell Signaling Technology), Tubulin (Vazyme, China).

### Immunofluorescence staining

U87MG cells were fixed in 4% paraformaldehyde for 30 min, permeabilized in 0.2% TritonX100 solution for 20 min, and then incubated with primary antibodies overnight at 4 °C. The primary antibodies (Ab) used are as follows: anti-*MAP2* Ab, anti-H3K9ac Ab, anti-H3K14ac Ab, anti-*TUBB3*, anti-*PCNA* (Cell Signaling Technology). Sections were then incubated with donkey-anti-rabbit-Alexa Fluor 555 (Thermo Fisher, USA) and DAPI for 30 min and mounted with coverslips. Finally, the samples were observed by laser-scanning confocal microscopy (Nikon, Japan).

### Electrophysiological measurements

U87MG cells were seeded on the coverslips (density 3 × 10^4^/mL) and mounted on an Olympus BX50WI microscope (Olympus Optical, Tokyo, Japan). Cells were perfused at the rate of 2 ml/min. Whole-cell recordings were performed 6 days after CM treatment with electrode resistance of 4–6 MΩ. The Na^+^ currents were recorded in voltage-clamp at membrane potentials between −70 and +40 mV in 10 mV steps. Action potentials, were recorded in current-clamp using input currents from −100 to +140 pA in a 20 pA steps.

### Flow cytometry analysis

Flow cytometry analysis was performed on a Gallios flow cytometer (Beckman Coulter, USA). For cell proliferation assessment, the cells were incubated with 10 μM EdU (Sigma-Aldrich) for 2 h, and EdU was chemically conjugated to 50 μM Auto 488 (Sigma-Aldrich) for 1 h and incubated with 2.5 μg/mL DAPI for another 30 min. For cell differentiation assessment, the cells were incubated with 100 μM Anti-*MAP2* Ab (Cell Signaling Technology) and Anti-*TUBB3* Ab (Cell Signaling Technology) for 12 h, and *MAP2*/*TUBB3* was conjugated to Alexa Fluor 555 for 1 h and incubated with 2.5 μg/mL DAPI for another 30 min.

### Immunohistochemical staining

Brain tissue from mice were quickly dissected and fixed in 4% paraformaldehyde overnight, then dehydrated with ascending grades of ethyl alcohol, and cleaned in xylene and embedded in paraffin, and sectioned at a thickness of 4 μm. Immunohistochemical (IHC) staining was performed as follows. According to the manufacturer s instructions of the IHC staining kit (#ab80436, Abcam, Cambridge, MA, USA). Slices were placed with antigen retrieval for 30 min, and in 3% hydrogen peroxide for 15 min after the gradients of deparaffinization and hydration. Cooling to room temperature, slices were incubated with indicated primary antibodies against Ki-67 (#9449S, Cell Signaling Technology), TUBB3 (#4466S, Cell Signaling Technology), MAP2 (#4542S, Cell Signaling Technology), H3K9ac (#9649S, Cell Signaling Technology) and H3K14ac (#7627S, Cell Signaling Technology)overnight at 4C, and then incubated with an HRP conjugated secondary antibody (#7074S, Cell Signaling Technology) at room temperature. Washing with PBS three times, slices were stained with a Diaminobenzidine (DAB) substrate chromogen mixture in turn. Images were captured using a digital pathology slide scanner (KF-TB-400, KFBIO, China). For quantification, the integrated optical density (IOD) of the IHC results were analyzed using Image-Pro Plus software and the relative IOD values between groups were compared. Three optical microscope fields in every animal were collected for counting and IOD of the six field (Vehicle) or nine fields (CM) were used for statistical analysis.

### RNA sequencing and data processing

Total RNA was extracted from the cells using Trizol (Invitrogen, CA, USA) according to the manual and sent to The Beijing Genomics Institute (BGI) for further processing and RNA-seq analysis. RNA-seq libraries were prepared using the Illumina TruSeq RNA Sample Preparation v2 Guide, and then the pair end 100 bases reads were generated on BGIseq500 platform (BGI-Shenzhen, China).

The raw reads were aligned to the hg38 reference genome via TopHat (version 2.1.0) with the default parameters. The gene count mapped reads with the parameter “-s no -a 20” using the HTSeq program. Gene count normalization and differential expression analysis were performed using the DESeq2 package.

GSEA analysis was carried out with the GSEA package (v.3.0) (Broad Institute), following the protocol described by Reimand et al. [34]. Only gene sets with *P* < 0.05 and FDR < 0.25 were considered as significantly enriched. Single sample GSEA score was conducted to evaluate the comprehensive expression level of a single gene set, using the R package GSVA [35].

### ChIP-seq assay and data processing

Treated cell samples were subject to ChIP-seq assay as described [36] and sent to BGI for further sequencing.

Raw reads were filtered first to remove low-quality or adapter sequences by SOAPnuke with parameters: filter -l 5 -q 0.5 -n 0.1 -Q 2 -5 1 -c 50. Cleaned reads were mapped to the reference genome of hg19 using SOAP2 (https://github.com/BGI-flexlab/SOAPnuke), whose parameters was -v 2 -s 3. We used MACS2 (https://github.com/macs3-project/MACS) to identify peaks (open chromatin regions), in which ‘-bw 200 -g XXX -s 50 -p 1e-5 -m 10 30 -broad -B -trackline’ was used. The significant regions were picked up if |M| >= 1 and *P*-value < =10^−5^. Also, the gene element annotation of peak or different enrichment peak from different samples was carried out by bedtools (https://bedtools.readthedocs.io/en/latest/) intersect mode with overlap 50%. The peak annotation, peak distribution, and Kyoto Encyclopedia of Genes and Genomes pathway enrichment were analyzed by ChIPseeker [37]. The promoter region was defined as −3 to +3 kb of transcription start site (TSS).

### Subcutaneous and intracranial xenograft GBM models

Animal studies were approved by the Animal Ethical and Welfare Committee of Sun Yat-sen University. The hind flanks of 4-week-old female BALB/c-nu/nu mice were subcutaneously inoculated with 2 × 10^5^ GSC-1 cells. The mice were randomly divided into 6 groups after tumor cell injection and were injected intraperitoneally once per day with vehicle, 20 mg/kg dbcAMP (Sigma-Aldrich, USA), 40 mg/kg luteolin (Selleck, USA), 20 mg/kg MS275 (Selleck), 100 mg/kg temozolomide (Topscience, USA) or a combination of these drugs. Tumor diameters were measured every other day with a caliper, and tumor volumes were estimated using the following formula: width^2^ × length/2 = *V* (mm^3^).

The orthotopic implantation of glioma cells was performed using 1 × 10^5^ GSCs. In brief, cells were injected 2 mm lateral and 0.5 mm anterior to the bregma and 2.5 mm below the skull of 4-week-old athymic nude mice. The mice were randomly divided into four groups and injected intraperitoneally with vehicle, 100 mg/kg RGFP109 (Selleck) plus 40 mg/kg luteolin, 100 mg/kg temozolomide or a combination once per day for 14 days (*n* = 8 animals for each group). The mice were monitored daily and killed when neurological symptoms were observed. Their brains were then dissected and fixed in formalin for H&E staining.

### Statistical analysis

Results in graphs are expressed as means ± SD, as indicated in the Figure legends, for the indicated number of observations. Numerical data of two independent samples were analyzed with Student’s t test (two-tailed, unequal variance). Numerical data of multiple independent samples were analyzed with ANOVA. The tumor volume data were analyzed by a repeated-measure ANOVA. Animal survival time was analyzed by Kaplan–Meier process. Significance was defined as *P* < 0.05.

## Supplementary information

Fig S1

Fig S2

Fig S3

Fig S4

Fig S5

Supplemental figures legend

drug information, the list of H3K9_H3K14 regulated genes and the list of CGGA_ssGSEA analyse in this article.

Original Data File

reproducibility check list

## Data Availability

The data that support the findings of this study are available from the corresponding author upon reasonable request. RNA-sequencing raw data has been deposited in the GEO repository with accession code GSE158015 and GSE215401, and ChIP-sequencing raw data with accession code GSE158272 and GSE215401.
