# Supplementary figures and images for: Differential regulation of H3K9/H3K14 acetylation by small molecules drives neuron-fate-induction of glioma cell
Source: Cell Death Dis. 2023 Feb 20;14(2):142. doi: 10.1038/s41419-023-05611-8 (PMC9941105; doi:10.1038/s41419-023-05611-8)

A

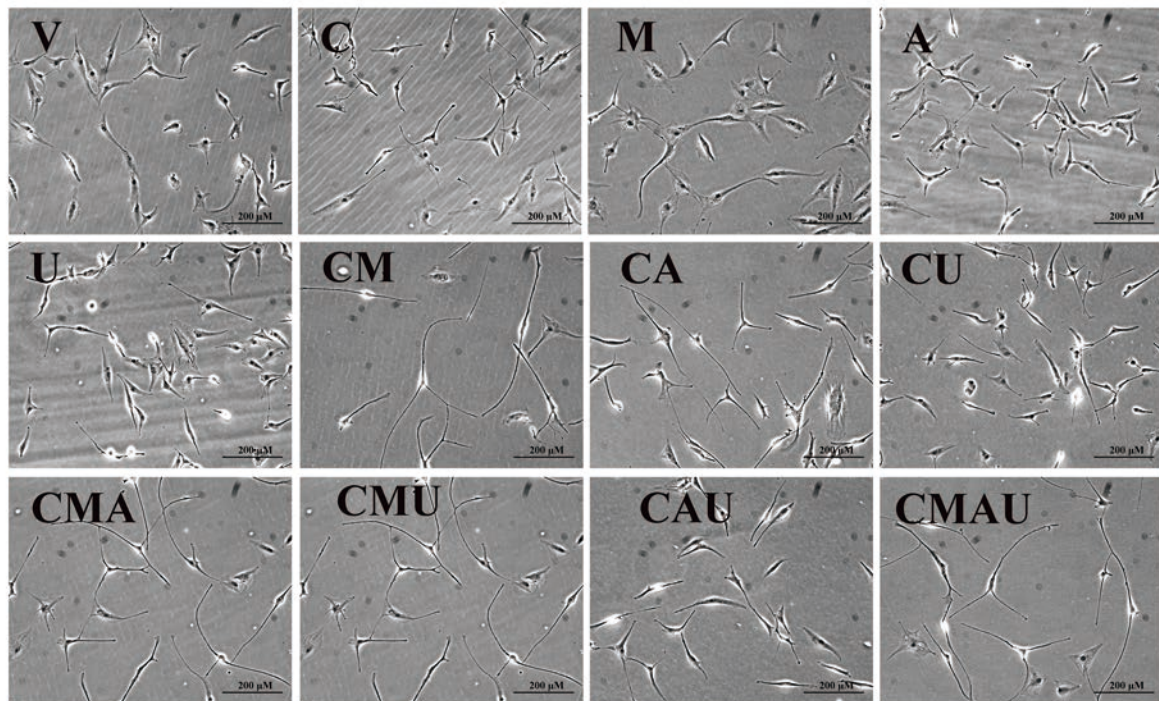

B

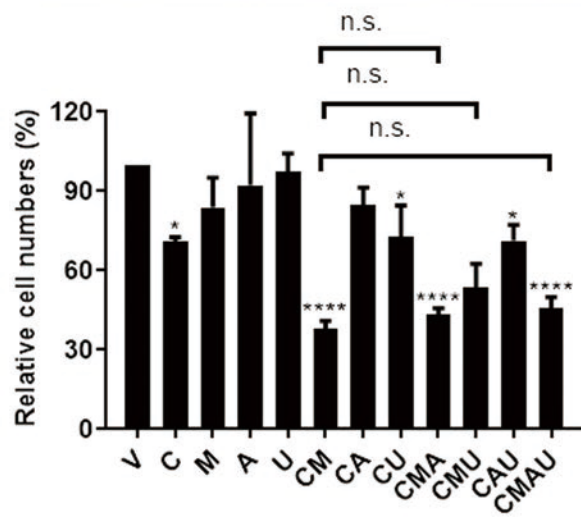

C

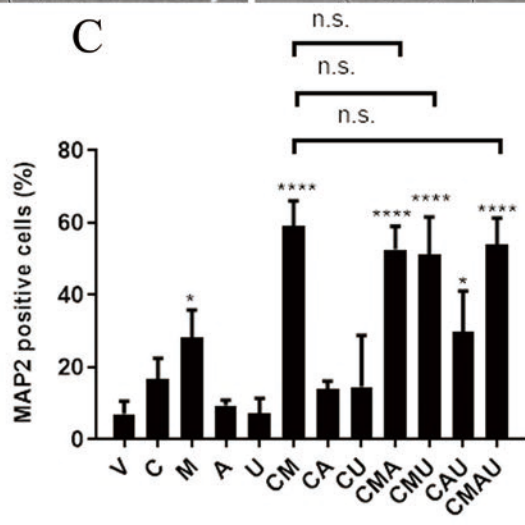

Supplement: Supplementary file 1 — Fig S1 [file 41419_2023_5611_MOESM1_ESM.pdf]

A

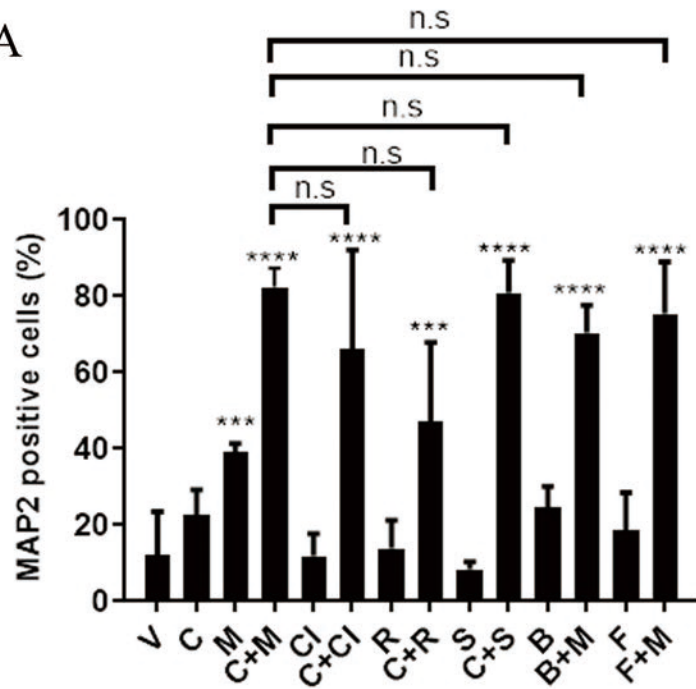

B

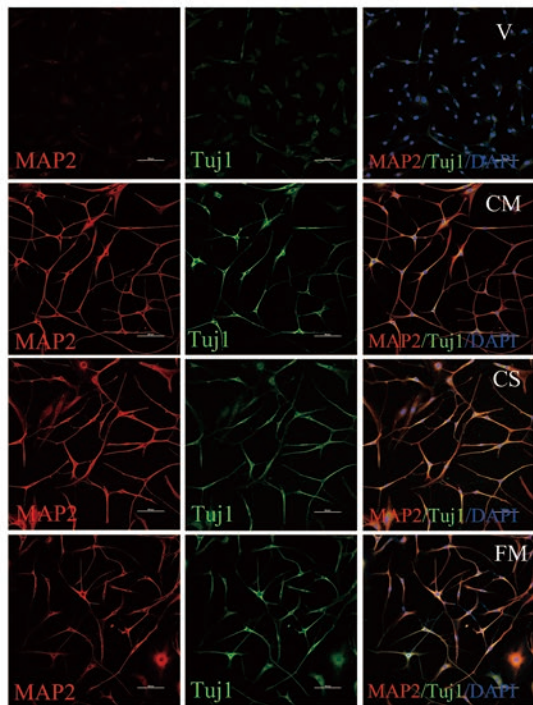

Supplement: Supplementary file 2 — Fig S2 [file 41419_2023_5611_MOESM2_ESM.pdf]

A

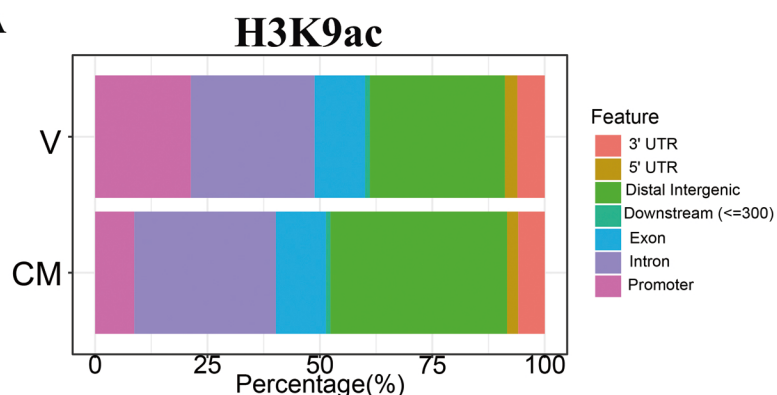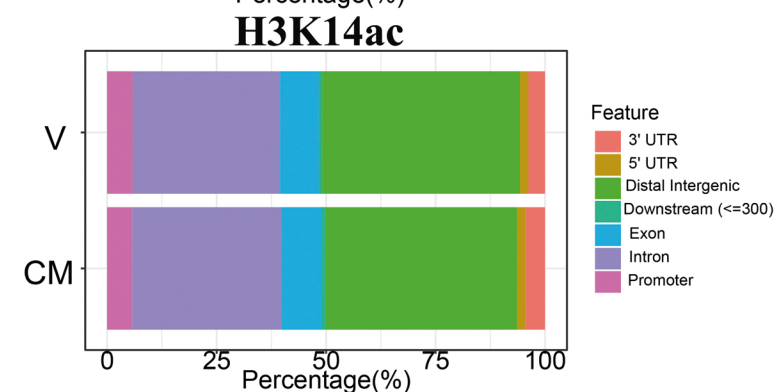

C

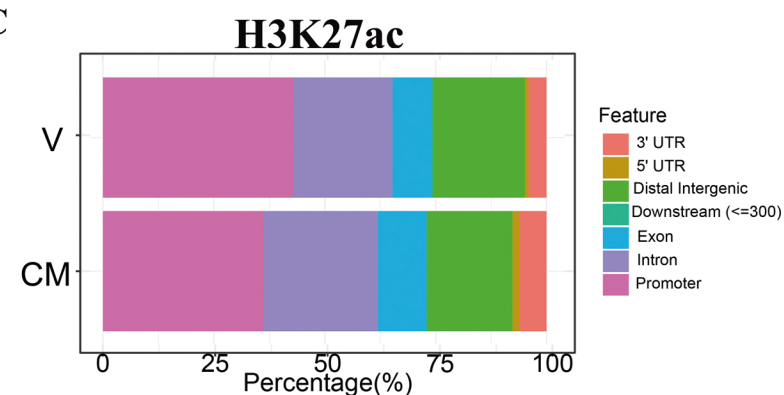

D

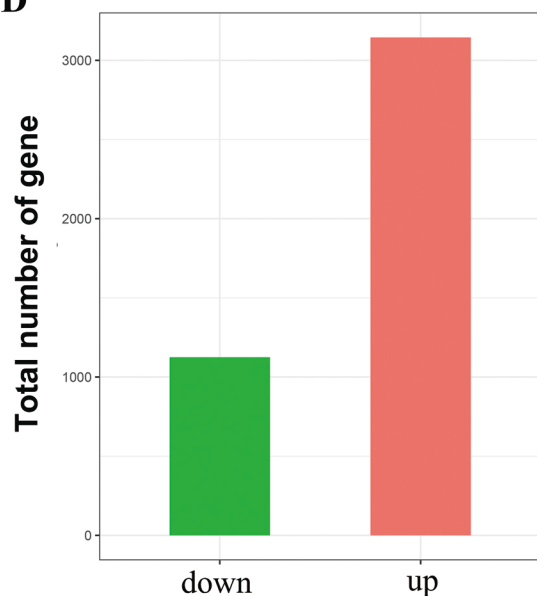

B

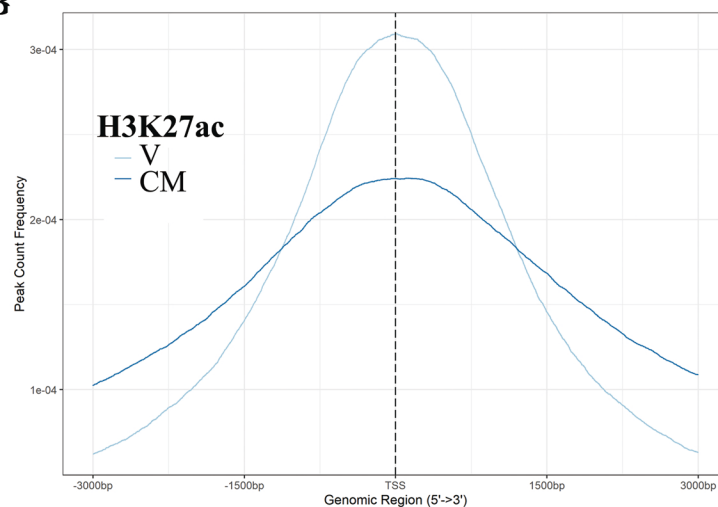

E

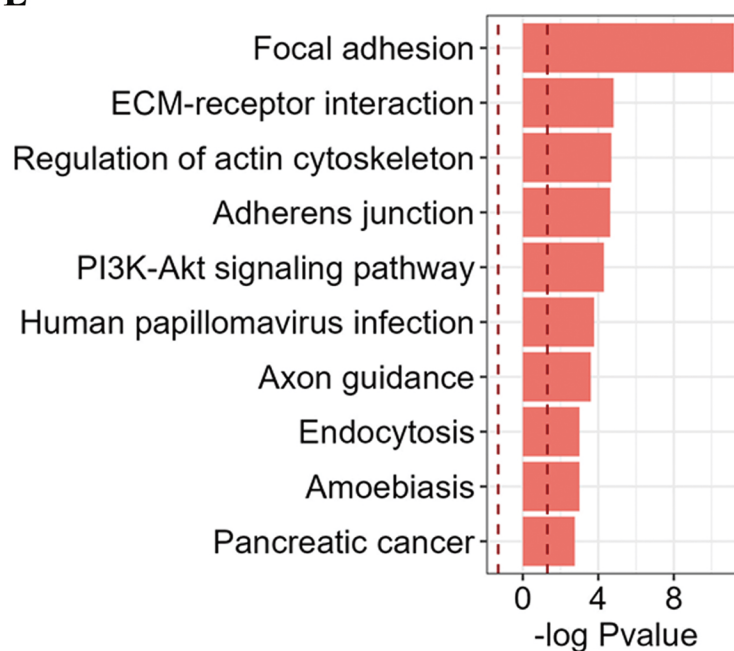

F

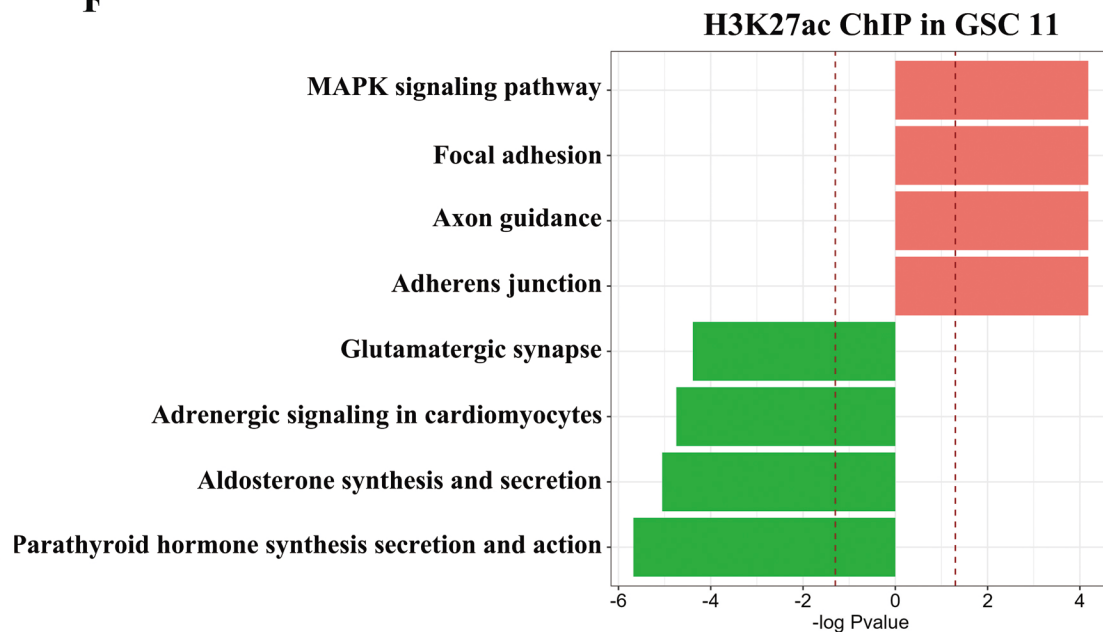

Supplement: Supplementary file 3 — Fig S3 [file 41419_2023_5611_MOESM3_ESM.pdf]

**A**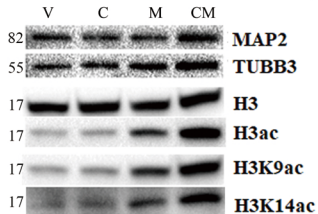**C**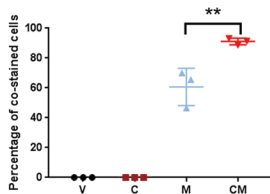**B**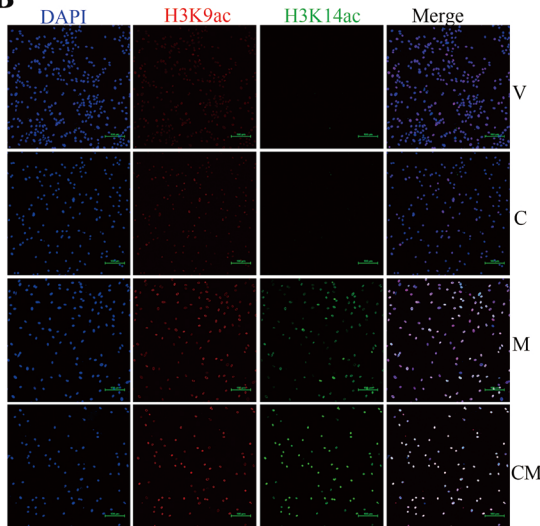

Supplement: Supplementary file 4 — Fig S4 [file 41419_2023_5611_MOESM4_ESM.pdf]

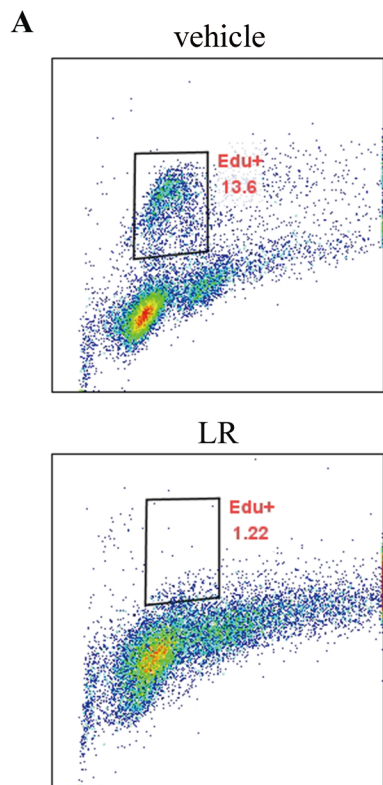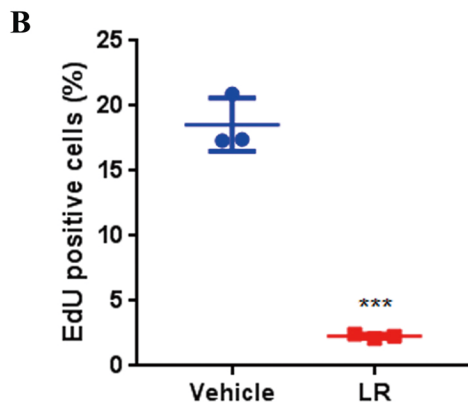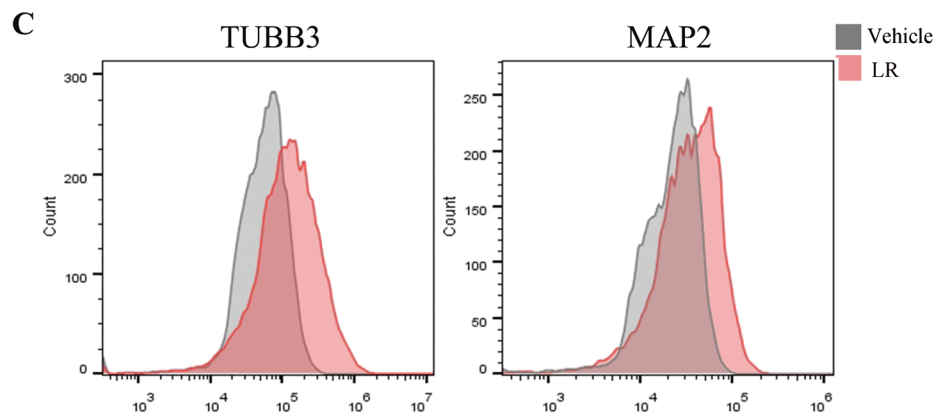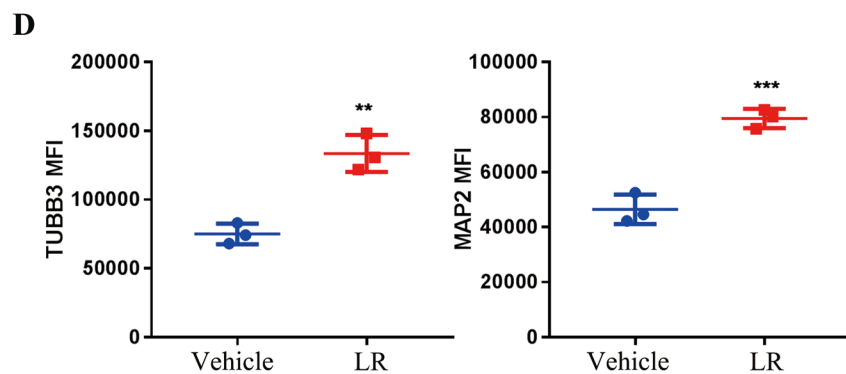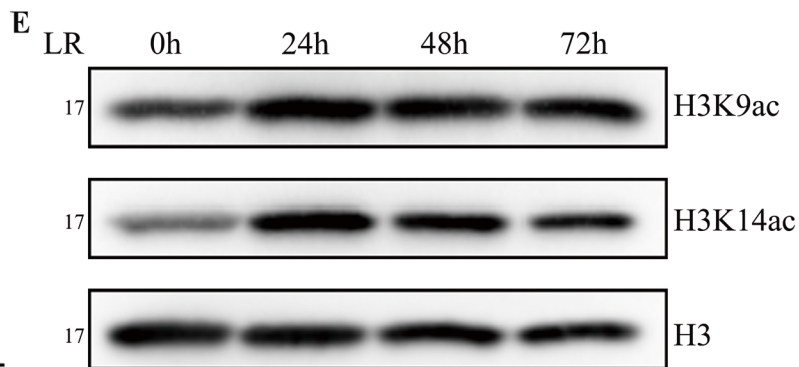

Supplement: Supplementary file 5 — Fig S5 [file 41419_2023_5611_MOESM5_ESM.pdf]
