# Supplementary material for: Differential regulation of H3K9/H3K14 acetylation by small molecules drives neuron-fate-induction of glioma cell
Source: Cell Death Dis. 2023 Feb 20;14(2):142. doi: 10.1038/s41419-023-05611-8 (PMC9941105; doi:10.1038/s41419-023-05611-8)
Supplement: Supplementary file 6 — Supplemental figures legend [file 41419_2023_5611_MOESM6_ESM.docx]

**Supplemental figures legend**

**Fig.S1 Effects of combinations of different epigenetic drugs on glioma differentiation.** (A-C) Morphology alteration of U87MG cells under different drug combinations for 2 days (A). V, Vehicle; C, 8-CPT-cAMP (0.5 mM); M, MS275 (1 μM); A, 5-azacytidine (a typical DNA methylase inhibitor, 1 μM); U, UNC1999 (a histone methylase inhibitor, 10 μM). The proliferation ability of these treated cells was quantified by the MTT method (B). Percentages of MAP2 positive cells were determined using immunofluorescence (C). Mean ± SD is shown in the bar plot (n=3 per group). The data were analyzed by a repeated-measure ANOVA. Treatment groups were compared with the V group. *, *P*＜0.05; ****, *P*＜0.0001; n.s, not significant.

**Fig.S2 Effects of cAMP activator plus HDAC inhibitor on glioma differentiation.** (A) Percentages of MAP2-positive U87MG cells under different drug combinations for 2 days. V, Vehicle; C, 8-CPT-cAMP (0.5 mM); M, MS275 (1 nM); CI, CI994 (HDAC1 inhibitor, 10 μM); R, HDAC3 inhibitor RGFP966 (0.5 μM); S, broadly-targeted HDAC inhibitor SAHA (0.5 μM); B, BIMP (cAMP activator, 0.25 mM); F, forskolin (cAMP activator, 10 μM). (B) Immunofluorescence analysis of MAP2 (red) and Tuj1 (green) in U87MG cells treated with V, C, M, CM for 2 days. DAPI is shown in blue. Mean ± SD is shown in the bar plot (n=3 per group). The data were analyzed by a repeated-measure ANOVA. Treatment groups were compared with the V group. ***, P＜0.001; ****, P＜0.0001; n.s, not significant.

**Fig.S3 ChIP-seq analysis of CM-induced H3K9ac/K14ac/K27ac.** (A-D) ChIP-seq assay in U87MG treated with CM for 48 h. (A) The percentages of peak location annotation in H3K9ac/H3K14ac region. (B) Average profile of H3K27ac peaks binding to TSS region. (C) The percentages of peak location annotation in H3K27ac region. (D) Gene counts regulated by H3K27ac identified. (E) Gene enrichment analysis of genes with altered H3K27ac tags. Red trace lines indicate adjusted P=0.05. (F) Gene enrichment analysis of genes with altered H3K27ac tags in GSC-11cells treated with CM for 48 h. Red trace lines indicate adjusted P=0.05. V: Vehicle; CM: 0.5 mM 8-CPT-cAMP plus 1 μM MS275.

**Fig.S4 Effects of 8-CPT-cAMP and/or MS-275 on H3K9ac and H3K14ac.** (A) Western blot analysis of MAP2, TUBB3, H3, H2Ac, H3K9ac and H3K14ac in U87MG cells after V, C, M, CM for 2 days. Tubulin as loading control. (B and C) Immunofluorescence analysis of H3K9ac (red) and H3K14ac (green) in U87MG cells after V, C, M, CM for 2 days. DAPI is shown in blue. Fluorescent images (B) and the percentage of co-stained cells (C). One-way ANOVA with Dunnett post-test. **, P＜0.01. V: Vehicle; CM: 0.5 mM 8-CPT-cAMP plus 1 μM MS275.

**Fig.S5 Luteolin cooperates with RGFP109 to induce neuronal differentiation of human GBM cells.** (A and B) Flow cytometry analysis and quantification for EdU+ cells in U87MG cells treated with LR (20 μM Luteolin plus 20 μM RGFP109) for 48 h. (C and D) Flow cytometry analysis and quantification for the protein levels of MAP2 and TUBB3 in U87MG cells treated with LR for 72 h. (E) Western blot analysis for H3K9ac/K14ac in U87 cells treated with LR for 24, 48, 72 h. Mean ± SD is shown in the bar plot (n=3 per group). All data were compared to vehicle with unpaired t test. **, P＜0.01, ***, P＜0.001.
