# Supplementary material for: Differential regulation of H3K9/H3K14 acetylation by small molecules drives neuron-fate-induction of glioma cell
Source: Cell Death Dis. 2023 Feb 20;14(2):142. doi: 10.1038/s41419-023-05611-8 (PMC9941105; doi:10.1038/s41419-023-05611-8)
Supplement: Supplementary file 7 — drug information, the list of H3K9_H3K14 regulated genes and the list of CGGA_ssGSEA analyse in this article. [file 41419_2023_5611_MOESM7_ESM.docx]

# Supplemental tables

# Table S1 drug information

| ***CAS Number*** | ***Concentration*** | ***molecular weight*** | ***Catalog Number*** | ***Target*** | ***Target(corrected)*** | ***Signal Pathway*** |
| --- | --- | --- | --- | --- | --- | --- |
| 923564-51-6 | 1mM | 974.61 | S1001 | Bcl-2 | Bcl-2 | Bcl-2 |
| 852808-04-9 | 1mM | 813.43 | S1002 | Autophagy | Bcl-2 | Bcl-2 |
| 796967-16-3 | 1mM | 375.41 | S1003 | CSF-1R | RTK | Tyrosine kinase |
| 912444-00-9 | 1mM | 244.29 | S1004 | PARP | PARP | PARP |
| 319460-85-0 | 1mM | 386.47 | S1005 | c-Kit | VEGFR | Growth Factor |
| 379231-04-6 | 1mM | 542.03 | S1006 | Bcr-Abl | Bcr-Abl | Bcr-Abl |
| 606143-52-6 | 1mM | 457.68 | S1008 | MEK | MEK | MEK |
| 915019-65-7 | 1mM | 469.55 | S1009 | ATM/ATR | PI3K | PI3K/Akt/mTOR |
| 656247-17-5 | 1mM | 539.62 | S1010 | FGFR | FGFR | Growth Factor |
| 439081-18-2 | 1mM | 485.94 | S1011 | EGFR | EGFR | Growth Factor |
| 179324-69-7 | 1mM | 384.24 | S1013 | Proteasome | Proteasome | Proteasome |
| 380843-75-4 | 1mM | 530.45 | S1014 | Src | Src-Abl | Tyrosine kinase |
| 288383-20-0 | 1mM | 450.51 | S1017 | VEGFR | VEGFR | Growth Factor |
| 405169-16-6 | 1mM | 392.43 | S1018 | c-Kit | FLT3 | Growth Factor |
| 212631-79-3 | 1mM | 478.67 | S1020 | MEK | MEK | MEK |
| 302962-49-8 | 1mM | 488.01 | S1021 | Bcr-Abl | Bcr-Abl | Bcr-Abl |
| 572924-54-0 | 1mM | 990.21 | S1022 | mTOR | mTOR | PI3K/Akt/mTOR |
| 183319-69-9 | 1mM | 429.90 | S1023 | Autophagy | HER1/EGFR | Growth Factor |
| 184475-35-2 | 1mM | 446.90 | S1025 | EGFR | EGFR | Growth Factor |
| 220127-57-1 | 1mM | 589.71 | S1026 | Bcr-Abl | Bcr-Abl | Bcr-Abl |
| 388082-77-7 | 1mM | 925.46 | S1028 | EGFR | EGFR | Growth Factor |
| 191732-72-6 | 1mM | 259.26 | S1029 | TNF-alpha | TNF-alpha | TNF-alpha |
| 857876-30-3 | 1mM | 569.44 | S1032 | c-Kit | VEGFR | Growth Factor |
| 641571-10-0 | 1mM | 529.52 | S1033 | Bcr-Abl | Bcr-Abl | Bcr-Abl |
| 391210-10-9 | 1mM | 482.19 | S1036 | MEK | MEK | MEK |
| 371935-74-9 | 1mM | 348.36 | S1038 | Autophagy | PI3K | PI3K/Akt/mTOR |
| 53123-88-9 | 1mM | 914.18 | S1039 | Autophagy | mTOR | PI3K/Akt/mTOR |
| 475207-59-1 | 1mM | 637.03 | S1040 | PDGFR | Raf | Serine/threonine kinase |
| 315702-99-9 | 1mM | 267.35 | S1041 | / | others | others |
| 341031-54-7 | 1mM | 532.56 | S1042 | c-Kit | RTK | Tyrosine kinase |
| 387867-13-2 | 1mM | 562.70 | S1043 | FLT3 | PDGFR | Growth Factor |
| 162635-04-3 | 1mM | 1030.29 | S1044 | mTOR | mTOR | PI3K/Akt/mTOR |
| 58880-19-6 | 1mM | 302.40 | S1045 | HDAC | HDAC | HDAC |
| 443913-73-3 | 1mM | 475.35 | S1046 | VEGFR | VEGFR | Growth Factor |
| 149647-78-9 | 1mM | 264.30 | S1047 | Autophagy | HDAC | HDAC |
| 639089-54-6 | 1mM | 464.59 | S1048 | Aurora Kinase | Aurora Kinase | Aurora Kinase |
| 129830-38-2 | 1mM | 320.26 | S1049 | Autophagy | ROCK1 | Serine/threonine kinase |
| 488832-69-5 | 1mM | 400.50 | S1052 | HSP (e.g. HSP90) | HSP | HSP |
| 209783-80-2 | 1mM | 376.41 | S1053 | HDAC | HDAC | HDAC |
| 170364-57-5 | 1mM | 515.61 | S1055 | PKC | PKC | PKC |
| 714971-09-2 | 1mM | 567.01 | S1056 | EGFR | EGFR | Growth Factor |
| 803712-79-0 | 1mM | 413.49 | S1057 | Autophagy | Bcl-2 | Bcl-2 |
| 763113-22-0 | 1mM | 434.46 | S1060 | PARP | PARP | PARP |
| 890090-75-2 | 1mM | 581.50 | S1061 | E3 Ligase | E3 Ligase | E3 Ligase |
| 790299-79-5 | 1mM | 498.64 | S1064 | c-Kit | Tyrosin kinase | Tyrosine kinase |
| 957054-30-7 | 1mM | 513.64 | S1065 | PI3K | PI3K | PI3K/Akt/mTOR |
| 301836-41-9 | 1mM | 384.39 | S1067 | TGF-beta/Smad | ALK | TGF-beta/Smad |
| 877399-52-5 | 1mM | 450.34 | S1068 | ALK | c-Met | Growth Factor |
| 747412-49-3 | 1mM | 465.54 | S1069 | HSP (e.g. HSP90) | HSP | HSP |
| 477575-56-7 | 1mM | 641.61 | S1070 | c-Met | c-Met | Growth Factor |
| 475110-96-4 | 1mM | 417.41 | S1072 | PI3K | PI3K | PI3K/Akt/mTOR |
| 280744-09-4 | 1mM | 371.22 | S1075 | GSK-3 | GSK-3 | GSK-3 |
| 152121-47-6 | 1mM | 377.43 | S1076 | p38 MAPK | p38 MAPK | p38 MAPK |
| 1032350-13-2 | 1mM | 480.39 | S1078 | Akt | Akt | PI3K/Akt/mTOR |
| 183322-45-4 | 1mM | 396.67 | S1079 | EGFR | EGFR | Growth Factor |
| 658084-23-2 | 1mM | 568.09 | S1080 | c-Met | c-Met | Growth Factor |
| 879085-55-9 | 1mM | 421.30 | S1082 | Hedgehog | P-gp | Hedgehog |
| 649735-46-6 | 1mM | 370.38 | S1084 | FGFR | VEGFR | Growth Factor |
| 414864-00-9 | 1mM | 318.35 | S1085 | HDAC | HDAC | HDAC |
| 160003-66-7 | 1mM | 292.03 | S1087 | PARP | PARP | PARP |
| 783355-60-2 | 1mM | 397.42 | S1090 | HDAC | HDAC | HDAC |
| 867160-71-2 | 1mM | 421.49 | S1091 | IGF-1R | IGF-1R | Growth Factor |
| 587871-26-9 | 1mM | 395.49 | S1092 | ATM/ATR | ATM/ATR | ATM/ATR |
| 1089283-49-7 | 1mM | 851.96 | S1093 | IGF-1R | IGF-1R | Growth Factor |
| 956905-27-4 | 1mM | 372.38 | S1094 | c-Met | c-Met | Growth Factor |
| 875320-29-9 | 1mM | 394.48 | S1096 | HDAC | HDAC | HDAC |
| 957217-65-1 | 1mM | 431.39 | S1097 | / | DprE1 | others |
| 459868-92-9 | 1mM | 421.36 | S1098 | PARP | PARP | PARP |
| 212141-51-0 | 1mM | 419.73 | S1101 | c-Kit | VEGFR | Growth Factor |
| 905281-76-7 | 1mM | 334.37 | S1104 | Raf | Raf | serine/threonine kinase |
| 154447-36-6 | 1mM | 307.34 | S1105 | Autophagy | PI3K | PI3K/Akt/mTOR |
| 827318-97-8 | 1mM | 474.55 | S1107 | Aurora Kinase | Aurora Kinase | Aurora Kinase |
| 761439-42-3 | 1mM | 614.20 | S1108 | ALK | ALK | TGF-beta/Smad |
| 755038-02-9 | 1mM | 521.66 | S1109 | PLK | PLK | Serine/threonine kinase |
| 1022150-57-7 | 1mM | 359.41 | S1112 | c-Met | c-Met | Growth Factor |
| 937174-76-0 | 1mM | 425.48 | S1113 | Akt | Akt | PI3K/Akt/mTOR |
| 943540-75-8 | 1mM | 377.35 | S1114 | c-Met | c-Met | Growth Factor |
| 827022-32-2 | 1mM | 483.99 | S1116 | CDK | CDK | CDK |
| 35943-35-2 | 1mM | 320.30 | S1117 | Akt | DNA synthesis | DNA/RNA Synthesis |
| 956958-53-5 | 1mM | 448.52 | S1118 | PI3K | PI3K | PI3K/Akt/mTOR |
| 159351-69-6 | 1mM | 958.22 | S1120 | mTOR | mTOR | PI3K/Akt/mTOR |
| 877877-35-5 | 1mM | 573.70 | S1121 | Bcl-2 | Bcl-2 | Bcl-2 |
| 726169-73-9 | 1mM | 396.44 | S1122 | HDAC | HDAC | HDAC |
| 154229-19-3 | 1mM | 349.51 | S1123 | P450 (e.g. CYP17) | P450 | P450 |
| 1001645-58-4 | 1mM | 506.02 | S1129 | Sirtuin | Sirtuin | Sirtuin |
| 781661-94-7 | 1mM | 443.29 | S1130 | E3 Ligase | E3 Ligase | E3 Ligase |
| 1028486-01-2 | 1mM | 518.92 | S1133 | Aurora Kinase | Aurora Kinase | Aurora Kinase |
| 896466-04-9 | 1mM | 381.43 | S1134 | Aurora Kinase | JAK | Aurora Kinase |
| 150399-23-8 | 1mM | 471.37 | S1135 | DHFR |  | Antimetabolite |
| 401900-40-1 | 1mM | 441.36 | S1140 | Androgen Receptor | Androgen Receptor | Androgen Receptor |
| 75747-14-7 | 1mM | 585.69 | S1141 | HSP (e.g. HSP90) | HSP | HSP |
| 467214-21-7 | 1mM | 653.21 | S1142 | HSP (e.g. HSP90) | HSP | HSP |
| 345627-80-7 | 1mM | 380.53 | S1145 | CDK | CDK | CDK |
| 4449-51-8 | 1mM | 411.62 | S1146 | Hedgehog | Hedgehog | Hedgehog |
| 722544-51-6 | 1mM | 507.56 | S1147 | Aurora Kinase | Aurora Kinase | Aurora Kinase |
| 114977-28-5 | 1mM | 807.88 | S1148 | Microtubule Associated | Microtubule Associated | Microtubule Associated |
| 122111-03-9 | 1mM | 299.66 | S1149 | Autophagy | DNA synthesis | DNA/RNA Synthesis |
| 33069-62-4 | 1mM | 853.91 | S1150 | Autophagy | Microtubule Associated | Microtubule Associated |
| 186692-46-6 | 1mM | 354.45 | S1153 | CDK | CDK | CDK |
| 1146618-41-8 | 1mM | 527.04 | S1154 | Aurora Kinase | Aurora Kinase | Aurora Kinase |
| 154361-50-9 | 1mM | 359.35 | S1156 | DNA/RNA Synthesis | DNA/RNA Synthesis | DNA/RNA Synthesis |
| 888216-25-9 | 1mM | 364.40 | S1159 | HSP (e.g. HSP90) | HSP | HSP |
| 417716-92-8 | 1mM | 426.85 | S1164 | VEGFR | VEGFR | Growth Factor |
| 141430-65-1 | 1mM | 371.41 | S1165 | Microtubule Associated | Microtubule Associated | Microtubule Associated |
| 15663-27-1 | 1mM | 300.05 | S1166 | DNA/RNA Synthesis | DNA/RNA Synthesis | DNA/RNA Synthesis |
| 1069-66-5 | 1mM | 166.19 | S1168 | Autophagy | HDAC | HDAC |
| 693228-63-6 | 1mM | 368.46 | S1171 | Aurora Kinase | Aurora Kinase | Aurora Kinase |
| 881202-45-5 | 1mM | 328.41 | S1172 | E3 Ligase | E3 Ligase | E3 Ligase |
| 1213269-23-8 | 1mM | 494.18 | S1173 | EGFR | EGFR | Growth Factor |
| 841205-47-8 | 1mM | 389.33 | S1174 | Androgen Receptor | Androgen Receptor | Androgen Receptor |
| 848695-25-0 | 1mM | 318.76 | S1175 | HSP (e.g. HSP90) | HSP | HSP |
| 755037-03-7 | 1mM | 482.82 | S1178 | c-Kit | VEGFR | Growth Factor |
| 284028-89-3 | 1mM | 312.31 | S1180 | Wnt/beta-catenin | Wnt/beta-catenin | Wnt/beta-catenin |
| 934353-76-1 | 1mM | 375.47 | S1181 | Aurora Kinase | Aurora Kinase | Aurora Kinase |
| 321674-73-1 | 1mM | 331.36 | S1186 | Telomerase | Telomerase | Telomerase |
| 677338-12-4 | 1mM | 351.36 | S1187 | PI3K | PI3K | PI3K/Akt/mTOR |
| 120511-73-1 | 1mM | 293.37 | S1188 | Aromatase | Aromatase | Aromatase |
| 170729-80-3 | 1mM | 534.43 | S1189 | Substance P | Substance P | Substance P |
| 90357-06-5 | 1mM | 430.37 | S1190 | Androgen Receptor | Androgen Receptor | Androgen Receptor |
| 129453-61-8 | 1mM | 606.77 | S1191 | Estrogen/progestogen Receptor | Estrogen/progestogen Receptor | Estrogen/progestogen Receptor |
| 112887-68-0 | 1mM | 458.49 | S1192 | DNA/RNA Synthesis | DNA/RNA Synthesis | DNA/RNA Synthesis |
| 50-35-1 | 1mM | 258.23 | S1193 | E3 Ligase | E3 Ligase | E3 Ligase |
| 1012054-59-9 | 1mM | 434.49 | S1194 | EGFR | EGFR | Growth Factor |
| 107868-30-4 | 1mM | 296.40 | S1196 | Aromatase | Aromatase | Aromatase |
| 97682-44-5 | 1mM | 586.68 | S1198 | Topoisomerase | Topoisomerase | Topoisomerase |
| 4291-63-8 | 1mM | 285.69 | S1199 | DNA/RNA Synthesis | DNA/RNA Synthesis | DNA/RNA Synthesis |
| 2353-33-5 | 1mM | 228.21 | S1200 | DNA Methyltransferase | DNA Methyltransferase | DNA Methyltransferase |
| 16208-51-8 | 1mM | 326.34 | S1201 | / | / | others |
| 372196-77-5 | 1mM | 488.74 | S1205 | DNA-PK | PI3K | PI3K/Akt/mTOR |
| 475108-18-0 | 1mM | 454.86 | S1207 | c-Kit | VEGFR | Growth Factor |
| 25316-40-9 | 1mM | 579.98 | S1208 | Autophagy | Topoisomerase | Topoisomerase |
| 51-21-8 | 1mM | 130.08 | S1209 | DNA/RNA Synthesis | DNA/RNA Synthesis | DNA/RNA Synthesis |
| 59-05-2 | 1mM | 454.44 | S1210 | DHFR | DHFR | antimetabolite |
| 99011-02-6 | 1mM | 240.30 | S1211 | / | / | others |
| 3543-75-7 | 1mM | 394.72 | S1212 | / | / | DNA damage |
| 121032-29-9 | 1mM | 297.27 | S1213 | DNA/RNA Synthesis | DNA/RNA Synthesis | DNA/RNA Synthesis |
| 9041-93-4 | 1mM | 1512.62 | S1214 | DNA/RNA Synthesis | DNA/RNA Synthesis | DNA/RNA Synthesis |
| 41575-94-4 | 1mM | 371.25 | S1215 | DNA/RNA Synthesis | DNA/RNA Synthesis | DNA/RNA Synthesis |
| 50-18-0 | 1mM | 261.09 | S1217 | DNA/RNA Synthesis | DNA/RNA Synthesis | DNA/RNA Synthesis |
| 123318-82-1 | 1mM | 303.68 | S1218 | DNA/RNA Synthesis | DNA/RNA Synthesis | DNA/RNA Synthesis |
| 371942-69-7 | 1mM | 467.48 | S1219 | PI3K | PI3K | PI3K/Akt/mTOR |
| 728033-96-3 | 1mM | 443.44 | S1220 | c-Kit | KDR | Tyrosine kinase |
| 4342-03-4 | 1mM | 182.18 | S1221 | DNA/RNA Synthesis | DNA/RNA Synthesis | DNA/RNA Synthesis |
| 56390-09-1 | 1mM | 579.98 | S1223 | Topoisomerase | Topoisomerase | Topoisomerase |
| 61825-94-3 | 1mM | 397.29 | S1224 | DNA/RNA Synthesis | DNA/RNA Synthesis | DNA/RNA Synthesis |
| 33419-42-0 | 1mM | 588.56 | S1225 | Topoisomerase | Topoisomerase | Topoisomerase |
| 938440-64-3 | 1mM | 465.54 | S1226 | mTOR | mTOR | PI3K/Akt/mTOR |
| 82640-04-8 | 1mM | 510.04 | S1227 | Estrogen/progestogen Receptor | Estrogen/progestogen Receptor | Estrogen/progestogen Receptor |
| 57852-57-0 | 1mM | 533.95 | S1228 | Topoisomerase | Topoisomerase | Topoisomerase |
| 75607-67-9 | 1mM | 365.21 | S1229 | DNA/RNA Synthesis | DNA/RNA Synthesis | DNA/RNA Synthesis |
| 119413-54-6 | 1mM | 457.91 | S1231 | Topoisomerase | Topoisomerase | Topoisomerase |
| 362-07-2 | 1mM | 302.41 | S1233 | HIF | HIF | HIF |
| 112809-51-5 | 1mM | 285.30 | S1235 | Aromatase | Aromatase | Aromatase |
| 6035-45-6 | 1mM | 601.58 | S1236 | / | DNA/RNA Synthesis | DNA/RNA Synthesis |
| 85622-93-1 | 1mM | 194.15 | S1237 | Autophagy | DNA damage | DNA damage |
| 2068-78-2 | 1mM | 923.04 | S1241 | Autophagy | Microtubule Associated | Microtubule Associated |
| 850879-09-3 | 1mM | 447.51 | S1244 | c-Kit | PDGF | Growth Factor |
| 57-22-7 | 1mM | 824.96 | S1248 | AChR | AChR | Microtubule Associated |
| 443797-96-4 | 1mM | 394.36 | S1249 | Aurora Kinase | Aurora Kinase | Aurora Kinase |
| 915087-33-1 | 1mM | 464.44 | S1250 | Androgen Receptor | Androgen Receptor | Androgen Receptor |
| 169590-42-5 | 1mM | 381.37 | S1261 | COX | COX | others |
| 219580-11-7 | 1mM | 523.67 | S1264 | FGFR | VEGFR | Growth Factor |
| 1062169-56-5 | 1mM | 495.53 | S1266 | mTOR | mTOR | PI3K/Akt/mTOR |
| 918504-65-1 | 1mM | 489.92 | S1267 | Raf | Raf | serine/threonine kinase |
| 371242-69-2 | 1mM | 397.43 | S1268 | PI3K | PI3K | PI3K/Akt/mTOR |
| 702675-74-9 | 1mM | 591.47 | S1274 | IκB/IKK | IκB/IKK | IκB/IKK |
| 645-05-6 | 1mM | 210.28 | S1278 | / | / | others |
| 61422-45-5 | 1mM | 257.26 | S1289 | DNA/RNA Synthesis | DNA/RNA Synthesis | DNA/RNA Synthesis |
| 152044-53-6 | 1mM | 493.66 | S1297 | Microtubule Associated | Microtubule Associated | Microtubule Associated |
| 50-91-9 | 1mM | 246.19 | S1299 | DNA/RNA Synthesis | DNA/RNA Synthesis | DNA/RNA Synthesis |
| 17902-23-7 | 1mM | 200.17 | S1300 | DNA/RNA Synthesis | DNA/RNA Synthesis | DNA/RNA Synthesis |
| 3778-73-2 | 1mM | 261.09 | S1302 | DNA/RNA Synthesis | DNA/RNA Synthesis | DNA/RNA Synthesis |
| 595-33-5 | 1mM | 384.51 | S1304 | Androgen Receptor | Androgen Receptor | Androgen Receptor |
| 50-44-2 | 1mM | 152.18 | S1305 | DNA/RNA Synthesis | DNA/RNA Synthesis | DNA/RNA Synthesis |
| 57248-88-1 | 1mM | 279.03 | S1311 | / | / | others |
| 18883-66-4 | 1mM | 265.22 | S1312 | / | / | others |
| 50-02-2 | 1mM | 392.46 | S1322 | Autophagy | Steroid | Steroid |
| 1225497-78-8 | 1mM | 473.47 | S1362 | PLK | PLK | Serine/threonine kinase |
| 152044-54-7 | 1mM | 507.68 | S1364 | Microtubule Associated | Microtubule Associated | Microtubule Associated |
| 130693-82-2 | 1mM | 360.90 | S1375 | Carbonic Anhydrase | Carbonic Anhydrase | others |
| 941678-49-5 | 1mM | 306.37 | S1378 | JAK | JAK | Tyrosine kinase |
| 4759-48-2 | 1mM | 300.44 | S1379 | Hydroxylase | Hydroxylase | Hydroxylase |
| 257933-82-7 | 1mM | 467.92 | S1392 | EGFR | EGFR | Growth Factor |
| 111406-87-2 | 1mM | 236.29 | S1443 | / | lipoxygenase | others |
| 336113-53-2 | 1mM | 517.06 | S1452 | Kinesin | Kinesin | Kinesin |
| 192185-72-1 | 1mM | 489.40 | S1453 | Transferase | Transferase | others |
| 186497-07-4 | 1mM | 424.43 | S1456 | Endothelin Receptor | Endothelin Receptor | others |
| 54573-75-0 | 1mM | 412.65 | S1467 | / | / | others |
| 356559-20-1 | 1mM | 343.42 | S1476 | TGF-beta/Smad | TGF-beta/Smad | TGF-beta/Smad |
| 497839-62-0 | 1mM | 440.58 | S1486 | EGFR | EGFR | Growth Factor |
| 718630-59-2 | 1mM | 361.48 | S1487 | CDK | CDK | CDK |
| 593960-11-3 | 1mM | 389.88 | S1489 | PI3K | PI3K | PI3K/Akt/mTOR |
| 943319-70-8 | 1mM | 532.56 | S1490 | Bcr-Abl | PDGFR | Growth Factor |
| 21679-14-1 | 1mM | 285.23 | S1491 | DNA/RNA Synthesis | DNA/RNA Synthesis | DNA/RNA Synthesis |
| 862507-23-1 | 1mM | 612.74 | S1494 | p38 MAPK | p38 MAPK | p38 MAPK |
| 128794-94-5 | 1mM | 433.49 | S1501 | Dehydrogenase | inosine monophosphate dehydrogenase | DNA/RNA Synthesis |
| 929016-96-6 | 1mM | 358.48 | S1515 | HDAC | HDAC | HDAC |
| 238750-77-1 | 1mM | 406.47 | S1522 | Aminopeptidase | Aminopeptidase | others |
| 1349796-36-6 | 1mM | 599.66 | S1523 | mTOR | mTOR | PI3K/Akt/mTOR |
| 844442-38-2 | 1mM | 382.24 | S1524 | CDK | CDK | CDK |
| 955365-80-7 | 1mM | 500.60 | S1525 | Wee1 | Wee1 | serine/threonine kinase |
| 950769-58-1 | 1mM | 560.67 | S1526 | FLT3 | FLT3 | Tyrosine kinase |
| 860352-01-8 | 1mM | 362.42 | S1532 | Chk | Chk | serine/threonine kinase |
| 841290-80-0 | 1mM | 470.45 | S1533 | Syk | Syk | Tyrosine kinase |
| 117570-53-3 | 1mM | 282.29 | S1537 | VDA | DT-diaphorase | others |
| 49843-98-3 | 1mM | 248.71 | S1541 | Sirtuin | Sirtuin | Sirtuin |
| 144060-53-7 | 1mM | 316.37 | S1547 | / | xanthine oxidase | others |
| 461432-26-8 | 1mM | 408.87 | S1548 | SGLT | SGLT | SGLT |
| 1009298-09-2 | 1mM | 465.54 | S1555 | mTOR | mTOR | PI3K/Akt/mTOR |
| 1025720-94-8 | 1mM | 512.89 | S1561 | Axl | c-Met | Growth Factor |
| 19171-19-8 | 1mM | 273.24 | S1567 | TNF-alpha | TNF-alpha | TNF-alpha |
| 925701-49-1 | 1mM | 547.67 | S1570 | ATM/ATR | ATM/ATR | ATM/ATR |
| 285983-48-4 | 1mM | 527.66 | S1574 | p38 MAPK | p38 MAPK | p38 MAPK |
| 948557-43-5 | 1mM | 439.53 | S1577 | Tie-2 | Tie-2 | others |
| 58970-76-6 | 1mM | 308.37 | S1591 | / | aminopeptidase B | others |
| 53-03-2 | 1mM | 358.43 | S1622 | / | steroid | steroid |
| 76-25-5 | 1mM | 434.50 | S1628 | / | Steroid | Steroid |
| 147-94-4 | 1mM | 243.22 | S1648 | DNA/RNA Synthesis | DNA/RNA Synthesis | DNA/RNA Synthesis |
| 302-79-4 | 1mM | 300.40 | S1653 | / | / | others |
| 163222-33-1 | 1mM | 409.40 | S1655 | / | / | others |
| 53-16-7 | 1mM | 270.37 | S1665 | Estrogen/progestogen Receptor | Estrogen/progestogen Receptor | Estrogen/progestogen Receptor |
| 125-84-8 | 1mM | 232.28 | S1672 | Aromatase | Aromatase | Aromatase |
| 97-77-8 | 1mM | 296.54 | S1680 | / | / | others |
| 1247-42-3 | 1mM | 372.46 | S1689 | / | steroid | steroid |
| 55-98-1 | 1mM | 246.30 | S1692 | / | / | others |
| 50-23-7 | 1mM | 362.46 | S1696 | / | Steroid | Steroid |
| 50-28-2 | 1mM | 272.38 | S1709 | / | Steroid | Steroid |
| 95058-81-4 | 1mM | 263.20 | S1714 | Autophagy | / | antimetabolite |
| 446-86-6 | 1mM | 277.26 | S1721 | / | DNA/RNA Synthesis | DNA/RNA Synthesis |
| 19767-45-4 | 1mM | 164.18 | S1735 | / | antioxidant | others |
| 89778-27-8 | 1mM | 598.08 | S1776 | Estrogen/progestogen Receptor | Estrogen/progestogen Receptor | Estrogen/progestogen Receptor |
| 320-67-2 | 1mM | 244.20 | S1782 | DNA Methyltransferase | DNA Methyltransferase | DNA Methyltransferase |
| 29767-20-2 | 1mM | 656.65 | S1787 | / | / | DNA damage |
| 79902-63-9 | 1mM | 418.57 | S1792 | / | HMG-CoA Reductase | HMG-CoA Reductase |
| 95635-55-5 | 1mM | 427.54 | S1799 | / | / | others |
| 13010-47-4 | 1mM | 233.70 | S1840 | / | / | DNA damage |
| 5959-95-5 | 1mM | 146.14 | S1893 | / | / | antimetabolite |
| 127-07-1 | 1mM | 76.05 | S1896 | / | DNA/RNA Synthesis | DNA/RNA Synthesis |
| 13311-84-7 | 1mM | 276.21 | S1908 | P450 (e.g. CYP17) | P450 | P450 |
| 93957-55-2 | 1mM | 433.45 | S1909 | HMG-CoA Reductase | HMG-CoA Reductase | HMG-CoA Reductase |
| 54965-24-1 | 1mM | 563.64 | S1972 | Autophagy | Estrogen/progestogen Receptor | Estrogen/progestogen Receptor |
| 366-70-1 | 1mM | 257.76 | S1995 | DNA/RNA Synthesis | DNA/RNA Synthesis | DNA/RNA Synthesis |
| 156-54-7 | 1mM | 110.09 | S1999 | / | HDAC | HDAC |
| 376348-65-1 | 1mM | 513.67 | S2003 | CCR5 | CCR5 | others |
| 869288-64-2 | 1mM | 491.49 | S2013 | FAK | FAK | Tyrosine kinase |
| 6055-19-2 | 1mM | 279.10 | S2057 | / | / | DNA damage |
| 153559-49-0 | 1mM | 348.00 | S2098 | / | retinoid receptor | retinoid receptor |
| 42971-09-5 | 1mM | 350.45 | S2110 | Sodium Channel | Sodium Channel | Sodium Channel |
| 231277-92-2 | 1mM | 581.06 | S2111 | EGFR | EGFR | Growth Factor |
| 698387-09-6 | 1mM | 557.04 | S2150 | EGFR | EGFR | Growth Factor |
| 956697-53-3 | 1mM | 485.50 | S2151 | Smoothened | Smoothened | Smoothened |
| 328543-09-5 | 1mM | 320.39 | S2178 | PARP | PARP | PARP |
| 1072833-77-2 | 1mM | 361.03 | S2180 | Proteasome | Proteasome | Proteasome |
| 1201902-80-8 | 1mM | 517.12 | S2181 | Proteasome | Proteasome | Proteasome |
| 940929-33-9 | 1mM | 553.52 | S2182 | Kinesin | Kinesin | Kinesin |
| 929095-18-1 | 1mM | 543.60 | S2193 | PLK | PLK | serine/threonine kinase |
| 1025065-69-3 | 1mM | 405.42 | S2198 | Pim | Pim | others |
| 1174046-72-0 | 1mM | 468.84 | S2201 | c-Met | VEGFR | Growth Factor |
| 183320-51-6 | 1mM | 415.87 | S2205 | EGFR | EGFR | Growth Factor |
| 1025687-58-4 | 1mM | 624.42 | S2206 | Syk | Syk | Tyrosine kinase |
| 566-48-3 | 1mM | 302.41 | S2208 | Aromatase | Aromatase | Aromatase |
| 208255-80-5 | 1mM | 432.46 | S2215 | Beta Amyloid | Gamma-secretase | Gamma-secretase |
| 136572-09-3 | 1mM | 677.18 | S2217 | Topoisomerase | Topoisomerase | Topoisomerase |
| 1056634-68-4 | 1mM | 414.46 | S2219 | JAK | JAK | Tyrosine kinase |
| 405554-55-4 | 1mM | 453.54 | S2220 | Raf | Raf | serine/threonine kinase |
| 901-47-3 | 1mM | 342.41 | S2225 | APC | E3 Ligase | E3 Ligase |
| 870281-82-6 | 1mM | 415.42 | S2226 | PI3K | PI3K | PI3K/Akt/mTOR |
| 700874-72-2 | 1mM | 369.42 | S2230 | TGF-beta/Smad | TGF-beta/Smad | TGF-beta/Smad |
| 332012-40-5 | 1mM | 409.83 | S2231 | c-Kit | VEGFR | Growth Factor |
| 755038-65-4 | 1mM | 618.81 | S2235 | PLK | PLK | Serine/threonine kinase |
| 914913-88-5 | 1mM | 406.43 | S2238 | mTOR | mTOR | PI3K/Akt/mTOR |
| 856243-80-6 | 1mM | 384.27 | S2243 | Bcr-Abl | deubiquitinase | Bcr-Abl |
| 935881-37-1 | 1mM | 312.36 | S2244 | HDAC | HDAC | HDAC |
| 1080622-86-1 | 1mM | 349.35 | S2245 | ATM/ATR | ATM/ATR | ATM/ATR |
| 944396-07-0 | 1mM | 410.39 | S2247 | PI3K | PI3K | PI3K/Akt/mTOR |
| 1009820-21-6 | 1mM | 349.77 | S2248 | PKC | PKC | PKC |
| 989-51-5 | 1mM | 458.37 | S2250 | / | / | others |
| 59865-13-3 | 1mM | 1202.61 | S2286 | / | / | others |
| 303-45-7 | 1mM | 518.56 | S2303 | / | dehydrogenase | others |
| 60-82-2 | 1mM | 274.27 | S2342 | / | SGLT | SGLT |
| 53003-10-4 | 1mM | 751.00 | S2352 | / | Aromatase | Aromatase |
| 117-39-5 | 1mM | 302.24 | S2391 | PI3K | PI3K | PI3K/Akt/mTOR |
| 303-98-0 | 1mM | 863.34 | S2398 | / | / | others |
| 481-74-3 | 1mM | 254.24 | S2406 | EGFR | mTOR | PI3K/Akt/mTOR |
| 152459-95-5 | 1mM | 493.60 | S2475 | PDGFR | c-kit | Growth Factor |
| 84625-61-6 | 1mM | 705.65 | S2476 | / | / | others |
| 70476-82-3 | 1mM | 517.40 | S2485 | / | / | antimetabolite |
| 24280-93-1 | 1mM | 320.34 | S2487 | / | IMPDH | DNA/RNA Synthesis |
| 122320-73-4 | 1mM | 357.43 | S2556 | PPAR | PPAR | PPAR |
| 71-58-9 | 1mM | 386.52 | S2567 | Estrogen/progestogen Receptor | Estrogen/progestogen Receptor | Estrogen/progestogen Receptor |
| 111025-46-8 | 1mM | 356.44 | S2590 | / | / | others |
| 84371-65-3 | 1mM | 429.59 | S2606 | Estrogen/progestogen Receptor | Estrogen/progestogen Receptor | Estrogen/progestogen Receptor |
| 50264-69-2 | 1mM | 321.16 | S2610 | / | hexokinase | others |
| 1035555-63-5 | 1mM | 504.23 | S2617 | MEK | MEK | MEK |
| 1062368-24-4 | 1mM | 406.48 | S2618 | TGF-beta/Smad | ALK | TGF-beta/Smad |
| 911222-45-2 | 1mM | 436.30 | S2626 | Chk | Chk | serine/threonine kinase |
| 405911-17-3 | 1mM | 618.51 | S2630 | Liver X Receptor | Liver X Receptor | others |
| 1020172-07-9 | 1mM | 553.59 | S2634 | Bcr-Abl | Bcr-Abl | Bcr-Abl |
| 503468-95-9 | 1mM | 413.49 | S2638 | DNA-PK | DNA-PK | DNA-PK |
| 1086062-66-9 | 1mM | 505.50 | S2658 | mTOR | mTOR | PI3K/Akt/mTOR |
| 471905-41-6 | 1mM | 442.90 | S2660 | Beta Amyloid | Gamma-secretase | Gamma-secretase |
| 1196109-52-0 | 1mM | 456.46 | S2666 | FAAH | FAAH | others |
| 871700-17-3 | 1mM | 615.39 | S2673 | MEK | MEK | MEK |
| 131740-09-5 | 1mM | 438.30 | S2679 | CDK | CDK | CDK |
| 936563-96-1 | 1mM | 440.50 | S2680 | BTK | BTK | Tyrosine kinase |
| 1092499-93-8 (free base) | 1mM | 563.47 | S2686 | JAK | JAK | Tyrosine kinase |
| 629664-81-9 | 1mM | 438.47 | S2694 | FXR | FXR | others |
| 844499-71-4 | 1mM | 360.39 | S2697 | AMPK | AMPK | others |
| 1007207-67-1 | 1mM | 377.42 | S2699 | mTOR | mTOR | PI3K/Akt/mTOR |
| 897016-82-9 | 1mM | 431.53 | S2700 | Src | Src | Tyrosine kinase |
| 700874-71-1 | 1mM | 441.52 | S2704 | TGF-beta/Smad | TGF-beta/Smad | TGF-beta/Smad |
| 209984-56-5 | 1mM | 463.48 | S2711 | Gamma-secretase | Gamma-secretase | Gamma-secretase |
| 30562-34-6 | 1mM | 560.64 | S2713 | Autophagy | HSP | HSP |
| 945595-80-2 | 1mM | 503.58 | S2719 | Aurora Kinase | Aurora Kinase | Aurora Kinase |
| 942487-16-3 | 1mM | 474.48 | S2725 | Aurora Kinase | Aurora Kinase | Aurora Kinase |
| 586379-66-0 | 1mM | 477.30 | S2726 | p38 MAPK | p38 MAPK | p38 MAPK |
| 1110813-31-4 | 1mM | 469.94 | S2727 | EGFR | ErbB | Growth Factor |
| 670220-88-9 | 1mM | 443.54 | S2730 | PDGFR | PDGFR | Growth Factor |
| 1124329-14-1 | 1mM | 452.55 | S2731 | Kinesin | Kinesin | Kinesin |
| 936091-26-8 | 1mM | 524.68 | S2736 | JAK | Jak | Tyrosine kinase |
| 315183-21-2 | 1mM | 392.49 | S2738 | Caspase | Caspase | others |
| 878739-06-1 | 1mM | 451.52 | S2746 | Raf | Raf | serine/threonine kinase |
| 1071992-99-8 | 1mM | 561.71 | S2754 | E3 Ligase | E3 Ligase | E3 Ligase |
| 842133-18-0 | 1mM | 444.52 | S2760 | SGLT | SGLT | SGLT |
| 5142-23-4 | 1mM | 149.15 | S2767 | Autophagy | PI3K | PI3K/Akt/mTOR |
| 211513-37-0 | 1mM | 389.59 | S2772 | CETP | CETP | others |
| 31430-18-9 | 1mM | 301.32 | S2775 | Autophagy | Bcr-Abl | Bcr-Abl |
| 278779-30-9 | 1mM | 542.84 | S2782 | FXR | FXR | others |
| 477600-75-2 | 1mM | 312.37 | S2789 | JAK | JAK | Tyrosine kinase |
| 425637-18-9 | 1mM | 438.48 | S2791 | PKC | PKC | PKC |
| 658084-64-1 | 1mM | 391.51 | S2799 | / | NAMPT | others |
| 410536-97-9 | 1mM | 394.47 | S2804 | Sirtuin | Sirtuin | Sirtuin |
| 1257704-57-6 | 1mM | 462.57 | S2806 | JAK | JAK | Tyrosine kinase |
| 1224844-38-5 | 1mM | 309.33 | S2811 | mTOR | mTOR | PI3K/Akt/mTOR |
| 1223001-51-1 | 1mM | 432.40 | S2817 | ATM/ATR | ATM/ATR | ATM/ATR |
| 48208-26-0 | 1mM | 334.33 | S2821 | DNA Methyltransferase | DNA Methyltransferase | DNA Methyltransferase |
| 507475-17-4 | 1mM | 279.29 | S2824 | IκB/IKK | IκB/IKK | IκB/IKK |
| 183321-86-0 | 1mM | 379.41 | S2826 | EGFR | EGFR | Growth Factor |
| 1222998-36-8 | 1mM | 607.62 | S2827 | Autophagy | mTOR | PI3K/Akt/mTOR |
| 717907-75-0 | 1mM | 507.49 | S2890 | FAK | Pyk2 | Tyrosine kinase |
| 941685-37-6 | 1mM | 306.37 | S2902 | JAK | Jak | Tyrosine kinase |
| 19542-67-7 | 1mM | 207.25 | S2913 | E2 conjugating | E2 conjugating | others |
| 252917-06-9 (free base) | 1mM | 501.80 | S2924 | GSK-3 | GSK-3 | GSK-3 |
| 444731-52-6 | 1mM | 437.52 | S3012 | c-Kit | c-kit | Growth Factor |
| 23541-50-6 | 1mM | 563.98 | S3035 | Telomerase | Telomerase | Telomerase |
| 1177-87-3 | 1mM | 434.50 | S3124 | / | Steroid | Steroid |
| 58579-51-4 | 1mM | 292.55 | S3172 | PDE | PDE | PDE |
| 38748-32-2 | 1mM | 360.40 | S3604 | / | / | others |
| 162359-56-0 | 1mM | 343.90 | S5002 | Bcr-Abl | Bcr-Abl | Bcr-Abl |

# Table S2 H3K9_H3K14 regulated genes

| K9ac_regulated_genes_in_GBM | AASS,AATF,ABCA1,ABL1,ABR,ACAA2,ACAD10,ACAD11,ACTR3,ADAR,ADK,ADORA1,AGMAT,AK6,AKAP8,AKAP8L,AKT1S1,ALDH1L1,ANAPC1,ANKFY1,ANKIB1,ANKRD10,ANKRD13C,ANKRD40,ANXA2,AP1G1,AP2B1,AP3M1,AP4M1,APIP,AREL1,ARID1B,ARL1,ARMC8,ARPC1A,ARPC3,ARPC4,ARPC4-TTLL3,ARV1,ASB7,ATF1,ATF5,ATG14,AZI2,BANP,BBIP1,BCCIP,BET1,BFAR,BRAP,BTF3,BTG3,C10orf88,C11orf80,C12orf10,C12orf65,C14orf178,C19orf48,C21orf59,CAMSAP2,CASP3,CASP3,CBLL1,CBX5,CBY1,CCDC34,CCDC6,CCNL2,CCP110,CCT6A,CCT6B,CDAN1,CDK12,CDV3,CEBPZ,CELSR1,CENPO,CEP135,CEP85,CEP89,CHD2,CHD2,CHD4,CHEK2,CHEK2,CIITA,CIZ1,CKS1B,CKS2,CLIC6,CLTC,CLVS2,CNIH3,CNOT2,CNOT7,COA6,COG5,COG8,COMMD1,COMT,COPA,CORO1C,CORO1C,COX11,CPEB2,CPSF2,CPT2,CRAT,CREBRF,CSNK1A1,CSNK1G1,CSNK1G3,CTCF,CTDSP2,CTDSPL2,CTTN,CYB561A3,DAB2,DARS,DAZAP2,DCTN6,DDI2,DDI2,DEDD,DEPDC1,DEPDC5,DESI2,DHRS7,DHX38,DIAPH1,DLGAP1,DNA2,DNAJC10,DPP8,DSE,DUS4L,DVL3,DYRK3,EBF2,EEF1G,EFTUD2,EGLN2,EIF2AK2,EIF3E,EIF4A1,EIF4G1,EIF6,ELP6,EPS15,ERCC6L2,ERH,ETV1,ETV5,EWSR1,FAF2,FAM120B,FAM171B,FAM193B,FAM227A,FAM32A,FANCD2,FBXL19,FBXO22,FBXO28,FBXO5,FBXW4,FBXW8,FCF1,FDPS,FERMT3,FGF1,FGF14,FHIT,FIG4,FIGN,FMNL2,FMNL3,FNBP4,FOXK1,FRS2,FSTL4,FUK,FYTTD1,GABPA,GAK,GARS,GCC1,GDAP2,GFM1,GFM2,GFPT2,GLT8D1,GMPPA,GNAS,GNG12,GNPDA2,GORASP2,GOSR2,GOT1,GPATCH1,GPBP1,GRHPR,GTF3C5,H3F3A,HADHA,HAUS8,HAX1,HBP1,HDAC7,HEATR5B,HEBP1,HIPK2,HMGB2,HMGXB3,HNRNPA1,HNRNPH1,HNRNPLL,HNRNPR,HNRNPU,HOMER1,HORMAD2,HPS4,HS3ST3A1,HSCB,HSP90AA1,HSPA9,HSPB8,IER2,IFIT2,IGF2BP3,IL4I1,ILK,ILKAP,IPO7,IQCB1,IQCG,IQCK,IRAK2,JOSD2,KARS,KAT5,KDM3B,KEAP1,KIAA0753,KIAA1217,KIF18A,KIF18B,KIF2C,KIF9,KIN,KLHL18,KMT2A,KNOP1,KPNA6,KRIT1,KY,LAPTM4A,LARP1,LASP1,LDLRAP1,LIN37,LONP1,LRRC29,LRRC40,LRRC41,LRRC49,LRSAM1,LSR,LTBP2,LYAR,LYRM1,MAP2K2,MAP2K7,MAPKAP1,MBD5,MBOAT7,MCC,MCL1,MCM4,MCM7,MCPH1,MDM4,MED13,MEF2A,MEIS2,METTL15,METTL4,MFSD5,MFSD8,MGLL,MIER1,MLF2,MMP14,MMP16,MMP24,MOB4,MORF4L1,MPHOSPH9,MRFAP1,MRPL12,MRPL32,MSI2,MSL2,MTA1,MXD3,MYC,MYH11,MYH9,MYO9B,MYO9B,MYOF,NAA15,NAP1L4,NAT14,NDC1,NDC80,NDEL1,NDUFAF7,NDUFB1,NDUFB3,NDUFC1,NELFA,NF1,NFIX,NFIX,NFIX,NHLRC3,NIP7,NIPBL,NOC3L,NOL10,NOM1,NOSIP,NPM1,NR2F1,NRG1,NRG2,NRIP1,NRIP3,NRXN2,NSA2,NSRP1,NT5E,NUDT21,NUDT22,NUFIP2,NUP214,NUP62,NUPR1,NVL,NXPE3,OAT,OGFOD1,OGG1,ORC1,OSBP,OSBPL10,P4HTM,PA2G4,PAPOLA,PARL,PARP8,PCDHGA1,PCDHGA10,PCDHGA11,PCDHGA12,PCDHGA4,PCDHGA5,PCDHGA6,PCDHGA7,PCDHGA9,PCDHGB1,PCDHGB2,PCDHGB3,PCDHGB5,PCDHGB7,PCDHGC3,PCF11,PDCD2,PDCD5,PEX2,PEX5,PFDN5,PGBD4,PHACTR1,PHACTR4,PI4K2A,PIK3CA,PIK3R1,PIK3R2,PKIB,PLAG1,PLEKHO1,PLIN3,PNPLA8,POLE3,POLR1A,POLR2D,POLR2M,POP1,PPHLN1,PPIP5K2,PPM1D,PPP2R1A,PPP2R5D,PPP2R5E,PPP3R1,PRDX1,PRDX3,PRIM1,PRIMPOL,PRKDC,PRPF38A,PRR11,PRR12,PRR14L,PSMA2,PSMA4,PSMB2,PSMB4,PSMC2,PSMC4,PSMD1,PSMD14,PSMD3,PSMG1,PSPH,PTCD3,PTGES3,PTRH2,PTRHD1,QSER1,RAB28,RAB2A,RAB32,RAB3GAP1,RABEP2,RAD17,RAD21,RALGDS,RAP2B,RAVER1,RBFOX2,RBL2,RBM12B,RBM17,RBPJ,RCE1,REXO4,RFXAP,RHBDD3,RHBDL2,RHOQ,RILPL2,RNASEH2C,RNASEK,RNF146,RNF167,RNF168,RNF185,RNF4,RPE,RPL17,RPL23A,RPL24,RPL27A,RPL3,RPL31,RPL35,RPL35A,RPL37A,RPL6,RPLP0,RPS13,RPTOR,RRP8,RTCA,RTN3,RTN4,RUNX2,S100PBP,SALL1,SAP30BP,SAR1A,SART3,SASH1,SASS6,SAT2,SATB2,SEC31A,SECISBP2,SENP3,SETD1B,SETD2,SETX,SF3A3,SHBG,SHC1,SKA2,SLC25A25,SLC25A3,SLC35B1,SLC35F6,SLC38A2,SLC38A7,SLFN5,SMARCA5,SMARCAD1,SMARCE1,SMC4,SNUPN,SNW1,SNX24,SNX7,SOCS4,SP3,SPCS1,SPRED2,SPRED2,SPTLC1,SRI,SRRD,SRSF11,SRSF2,SRSF4,SSBP1,SSBP2,SSC5D,SSH1,SSR2,ST5,ST7L,STAM,STAU1,STIL,STRADA,STRN4,STX10,STX16,STX5,STXBP4,SUDS3,SUPT6H,SURF6,SYF2,SYMPK,SYNJ2,SYNJ2BP,SYNJ2BP-COX16,SZRD1,TAB1,TADA3,TAF1C,TAF9,TAX1BP1,TBC1D17,TBC1D22A,TBK1,TDP1,TERF2IP,TGIF1,THAP10,THUMPD1,THUMPD3,TIGD6,TIPIN,TJAP1,TLCD1,TMCC1,TMEM138,TMEM167A,TMEM175,TMEM208,TMEM209,TMEM43,TMX2,TNIP1,TOPBP1,TP53,TPM4,TRA2A,TRAPPC3,TRIM4,TRIO,TRIO,TRIO,TRIO,TRIP12,TRMT13,TSC1,TSEN34,TSTA3,TULP3,TUT1,TXLNA,TXNL4B,TXNRD1,TXNRD2,U2AF1,UBA5,UBAC2,UBE2D2,UBE2L3,UBXN7,UNC5B,UPF2,URM1,USE1,USP1,USP10,UTP6,VIM,VPS29,VPS37A,VPS37B,VPS37C,VPS72,VRK1,WASL,WDHD1,WDR20,WDR3,WDR6,WDR74,WRAP53,WWP2,XKR8,XRCC4,YARS,YIPF4,YKT6,YLPM1,YWHAE,YWHAZ,ZBTB20,ZBTB20,ZBTB34,ZBTB38,ZBTB49,ZC3H13,ZC3H15,ZC3H4,ZC3H7A,ZDHHC18,ZDHHC7,ZFAND2A,ZFP36L1,ZMYM4,ZMYM6,ZNF106,ZNF136,ZNF19,ZNF212,ZNF214,ZNF215,ZNF346,ZNF398,ZNF652,ZNF770,ZNF830,ZNF836,ZW10 |
| --- | --- |
| K14ac_regulated_genes_in_GBM | ADCY5,ALPL,ASIC2,BAZ2A,BCL11B,CCDC170,CD34,CDH4,CMTM8,CNGB3,CNTNAP2,CNTNAP2,CPNE4,CTNNA3,CYP3A7,DGKI,DIO1,DPF3,EEF1A2,ELAVL4,ERG,ESRRG,EYA2,GALNT18,GALNT2,GPC6,GPC6,GRID1,GRIK3,HECTD4,HIVEP3,IL19,INSC,JAKMIP1,KCNQ1,KDM7A,KRAS,MAP2,MEGF11,MYO1D,NCKAP5,NOS1,NRF1,NRF1,PHACTR3,PLCG2,PLCL1,PREP,PTPRT,PTPRT,RBFOX3,RNF157,RPH3A,SLC22A13,SLC6A12,TBC1D1,TMEM241,TRIM66,UBE3C,ZNF704 |

# Table S3 CGGA_ssGSEA

| Patient | K9ac | K14ac | K9K14ac | PRS_type | Histology | Grade | Gender | Age | OS | Censor | Radio_status | Chemo_status | IDH_mutation_status | X1p19q_codeletion_status | group_list | GBMLGG |
| --- | --- | --- | --- | --- | --- | --- | --- | --- | --- | --- | --- | --- | --- | --- | --- | --- |
| CGGA_1002 | 0.143330816 | 0.320414925 | -0.177084109 | Primary | AA | WHO III | Female | 43 | 305 | 1 | 1 | 1 | Wildtype | Non-codel | low | 1 |
| CGGA_1003 | -0.337102728 | 0.03670561 | -0.373808338 | Primary | OA | WHO II | Female | 47 | 3087 | 0 | 0 | 1 | Mutant | Codel | low | 1 |
| CGGA_1010 | -0.048395699 | 0.03243792 | -0.080833619 | Primary | A | WHO II | Male | 45 | 246 | 1 | 1 | 1 | Mutant | NA | high | 1 |
| CGGA_1012 | 0.225331596 | -0.249324004 | 0.4746556 | Recurrent | rO | WHO II | Male | 45 | 3116 | 0 | 1 | 1 | Mutant | Non-codel | high | 1 |
| CGGA_1014 | 0.21058342 | 0.203449066 | 0.007134354 | Primary | A | WHO II | Male | 42 | 263 | 1 | 0 | 1 | Wildtype | Non-codel | high | 1 |
| CGGA_1017 | -0.059344334 | 0.55612031 | -0.615464644 | Primary | GBM | WHO IV | Female | 29 | 768 | 1 | 1 | 0 | Wildtype | Non-codel | low | 0 |
| CGGA_1018 | -0.198757869 | 0.094502725 | -0.293260594 | Recurrent | rO | WHO II | Male | 50 | 2527 | 1 | 1 | 1 | Mutant | Codel | low | 1 |
| CGGA_103 | -0.153079518 | 0.28428594 | -0.437365458 | Primary | AA | WHO III | Male | 56 | 1188 | 1 | 1 | 0 | Mutant | Non-codel | low | 1 |
| CGGA_1030 | -0.131224584 | 0.240071367 | -0.371295951 | Primary | OA | WHO II | Female | 40 | 2934 | 0 | 1 | 1 | Mutant | Non-codel | low | 1 |
| CGGA_1032 | -0.243469756 | 0.22639484 | -0.469864596 | Primary | A | WHO II | Female | 41 | NA | NA | NA | NA | Wildtype | NA | low | 1 |
| CGGA_1033 | -0.255478347 | 0.142981501 | -0.398459848 | Primary | OA | WHO II | Male | 44 | 1322 | 0 | 0 | 1 | NA | Non-codel | low | 1 |
| CGGA_1036 | 0.312064445 | -0.297931508 | 0.609995953 | Primary | GBM | WHO IV | Male | 41 | 1166 | 1 | 1 | 1 | Wildtype | Non-codel | high | 0 |
| CGGA_1037 | -0.389321815 | -0.092061282 | -0.297260533 | Primary | A | WHO II | Male | 44 | 780 | 0 | 1 | 0 | NA | Codel | low | 1 |
| CGGA_1041 | -0.046874853 | 0.518820758 | -0.565695611 | Primary | GBM | WHO IV | Male | 58 | 2863 | 0 | 1 | 1 | Wildtype | Non-codel | low | 0 |
| CGGA_1048 | -0.455210528 | 0.026595539 | -0.481806067 | Primary | OA | WHO II | Female | 27 | 703 | 0 | 0 | 1 | Wildtype | Non-codel | low | 1 |
| CGGA_1051 | -0.301642768 | 0.239953253 | -0.541596021 | Primary | A | WHO II | Male | 47 | 838 | 1 | NA | NA | Wildtype | Codel | low | 1 |
| CGGA_1055 | -0.035626575 | -0.128100394 | 0.092473819 | Primary | AA | WHO III | Male | 43 | 1611 | 1 | 1 | 1 | Mutant | Non-codel | high | 1 |
| CGGA_1057 | -0.412632552 | -0.155964837 | -0.256667715 | Primary | OA | WHO II | Female | 57 | 1549 | 1 | 1 | 1 | Wildtype | Non-codel | low | 1 |
| CGGA_1058 | -0.434051454 | -0.016165525 | -0.417885929 | Recurrent | rA | WHO II | Male | 34 | 995 | 1 | 1 | 1 | Mutant | Non-codel | low | 1 |
| CGGA_106 | -0.43383882 | 0.003231333 | -0.437070153 | Recurrent | rA | WHO II | Male | 30 | 4374 | 0 | 0 | 1 | Wildtype | Non-codel | low | 1 |
| CGGA_1063 | -0.357964832 | 0.15246743 | -0.510432262 | Primary | A | WHO II | Male | 29 | NA | NA | NA | NA | Mutant | NA | low | 1 |
| CGGA_1065 | -0.483622691 | -0.192619715 | -0.291002976 | Primary | OA | WHO II | Female | 48 | 2682 | 0 | 1 | 1 | Mutant | Codel | low | 1 |
| CGGA_1066 | 0.197080829 | 0.195275459 | 0.00180537 | Primary | A | WHO II | Female | 34 | 723 | 1 | 1 | 0 | Mutant | Non-codel | high | 1 |
| CGGA_1069 | 0.053231551 | -0.090078258 | 0.143309809 | Recurrent | rA | WHO II | Male | 47 | NA | NA | NA | NA | Mutant | Non-codel | high | 1 |
| CGGA_107 | 0.481638419 | -0.019124381 | 0.5007628 | Recurrent | rAA | WHO III | Female | 38 | 2156 | 1 | 0 | 0 | Wildtype | Non-codel | high | 1 |
| CGGA_1075 | 0.040329697 | -0.319910006 | 0.360239703 | Primary | GBM | WHO IV | Male | 72 | 720 | 0 | 1 | 1 | Wildtype | Non-codel | high | 0 |
| CGGA_108 | 0.295387229 | 0.155878419 | 0.13950881 | Recurrent | rAA | WHO III | Male | 44 | 366 | 1 | 1 | 1 | Wildtype | Non-codel | high | 1 |
| CGGA_1082 | -0.45956005 | -0.321874568 | -0.137685482 | Primary | A | WHO II | Female | 55 | NA | NA | NA | NA | Mutant | Non-codel | low | 1 |
| CGGA_1086 | -0.176115817 | 0.095609577 | -0.271725394 | Primary | GBM | WHO IV | Female | 65 | 1977 | 1 | 1 | 1 | Wildtype | NA | low | 0 |
| CGGA_1087 | -0.152905757 | 0.041365468 | -0.194271225 | Primary | OA | WHO II | Female | 36 | 701 | 0 | 0 | 1 | Mutant | Codel | low | 1 |
| CGGA_1097 | -0.074791266 | -0.247642707 | 0.172851441 | Primary | OA | WHO II | Male | 30 | 1378 | 1 | 1 | 1 | Wildtype | Non-codel | high | 1 |
| CGGA_1100 | -0.290250132 | 0.249633277 | -0.539883409 | Primary | O | WHO II | Female | 61 | 2596 | 0 | 1 | 1 | Mutant | NA | low | 1 |
| CGGA_1101 | -0.255046768 | 0.24925409 | -0.504300858 | Primary | OA | WHO II | Female | 35 | 214 | 1 | 0 | 0 | Wildtype | NA | low | 1 |
| CGGA_1103 | 0.608214475 | 0.142125103 | 0.466089372 | Primary | GBM | WHO IV | Female | 35 | 585 | 1 | 1 | 1 | Mutant | Non-codel | high | 0 |
| CGGA_1106 | 0.143568148 | -0.275686787 | 0.419254935 | Primary | GBM | WHO IV | Male | 37 | 420 | 1 | 1 | 1 | Wildtype | Non-codel | high | 0 |
| CGGA_1108 | -0.174399876 | -0.206572364 | 0.032172488 | Primary | AOA | WHO III | Female | 39 | 870 | 1 | 1 | 1 | Wildtype | Non-codel | high | 1 |
| CGGA_1111 | 0.498161325 | -0.14986253 | 0.648023855 | Primary | AOA | WHO III | Male | 45 | 2582 | 0 | 1 | 1 | Mutant | Non-codel | high | 1 |
| CGGA_112 | -0.260594233 | -0.034313781 | -0.226280452 | Primary | OA | WHO II | Male | 17 | 1534 | 1 | 1 | 1 | Mutant | Non-codel | low | 1 |
| CGGA_1120 | 0.00415336 | 0.305548184 | -0.301394824 | Primary | AOA | WHO III | Female | 58 | 422 | 1 | 1 | 1 | Mutant | NA | low | 1 |
| CGGA_1121 | -0.241554805 | -0.368290661 | 0.126735856 | Recurrent | rAOA | WHO III | Female | 29 | 2560 | 0 | 1 | 1 | Mutant | Non-codel | high | 1 |
| CGGA_1126 | -0.011195491 | -0.391242554 | 0.380047063 | Primary | AOA | WHO III | Male | 44 | 784 | 1 | 1 | 1 | Mutant | Non-codel | high | 1 |
| CGGA_1127 | -0.584380898 | -0.199751763 | -0.384629135 | Primary | O | WHO II | Male | 33 | 2547 | 0 | 1 | 1 | NA | Codel | low | 1 |
| CGGA_1130 | 0.134906711 | 0.017633103 | 0.117273608 | Recurrent | rGBM | WHO IV | Female | 23 | NA | NA | 1 | 0 | Mutant | Non-codel | high | 0 |
| CGGA_1131 | 0.538301163 | 0.024792194 | 0.513508969 | Primary | AOA | WHO III | Female | 46 | 2533 | 0 | 1 | 1 | Mutant | Non-codel | high | 1 |
| CGGA_1132 | -0.507771582 | -0.044648002 | -0.46312358 | Recurrent | rAOA | WHO III | Female | 33 | 2533 | 0 | 1 | 1 | Mutant | Codel | low | 1 |
| CGGA_1134 | 0.227160417 | -0.231343501 | 0.458503918 | Primary | GBM | WHO IV | Female | 56 | 59 | 1 | 0 | 1 | Wildtype | Non-codel | high | 0 |
| CGGA_1135 | 0.068734745 | -0.324515422 | 0.393250167 | Primary | GBM | WHO IV | Male | 40 | 1172 | 1 | 1 | 1 | Wildtype | Non-codel | high | 0 |
| CGGA_1137 | 0.078373221 | 0.330953547 | -0.252580326 | Primary | AOA | WHO III | Male | 58 | 2519 | 0 | 1 | 1 | Wildtype | NA | low | 1 |
| CGGA_1138 | 0.134176583 | -0.44795123 | 0.582127813 | Primary | GBM | WHO IV | Male | 54 | 411 | 1 | 1 | 1 | Wildtype | Non-codel | high | 0 |
| CGGA_1141 | -0.213658959 | 0.275718484 | -0.489377443 | Primary | AOA | WHO III | Male | 65 | 432 | 1 | NA | NA | Wildtype | Non-codel | low | 1 |
| CGGA_1142 | 0.119678046 | -0.486777707 | 0.606455753 | Primary | GBM | WHO IV | Male | 60 | 1005 | 1 | 1 | 1 | Wildtype | Non-codel | high | 0 |
| CGGA_1144 | -0.174160494 | 0.400481321 | -0.574641815 | Primary | AA | WHO III | Female | 39 | 2505 | 0 | 1 | 1 | Wildtype | Non-codel | low | 1 |
| CGGA_1147 | -0.440899133 | 0.067287571 | -0.508186704 | Primary | A | WHO II | Male | 36 | 2498 | 0 | 0 | 1 | Mutant | Non-codel | low | 1 |
| CGGA_1148 | -0.884424324 | -0.457347512 | -0.427076812 | Primary | A | WHO II | Male | 37 | 596 | 1 | 1 | 1 | Mutant | Non-codel | low | 1 |
| CGGA_1152 | -0.366341741 | -0.143420043 | -0.222921698 | Primary | A | WHO II | Male | 13 | 2484 | 0 | 1 | 1 | Wildtype | Non-codel | low | 1 |
| CGGA_1154 | 0.140499914 | -0.069618737 | 0.210118651 | Recurrent | rAOA | WHO III | Female | 33 | 383 | 1 | NA | NA | Mutant | Non-codel | high | 1 |
| CGGA_1155 | -0.165777525 | -0.033020408 | -0.132757117 | Primary | AA | WHO III | Male | 43 | 1373 | 1 | 1 | 1 | Mutant | Codel | low | 1 |
| CGGA_1156 | -0.427890259 | -0.174578062 | -0.253312197 | Primary | OA | WHO II | Female | 41 | 2477 | 0 | 1 | 0 | Mutant | Non-codel | low | 1 |
| CGGA_1157 | -0.425860762 | -0.149464861 | -0.276395901 | Primary | A | WHO II | Male | 64 | 741 | 1 | 1 | 1 | Wildtype | Non-codel | low | 1 |
| CGGA_1158 | -0.667796512 | -0.221745504 | -0.446051008 | Primary | OA | WHO II | Male | 39 | 2471 | 0 | 1 | 1 | Mutant | Codel | low | 1 |
| CGGA_1159 | -0.232694457 | 0.281244724 | -0.513939181 | Primary | OA | WHO II | Male | 51 | 2464 | 0 | 0 | 1 | Mutant | Non-codel | low | 1 |
| CGGA_1161 | -0.192663551 | 0.14562396 | -0.338287511 | Primary | AOA | WHO III | Male | 36 | 2463 | 0 | 1 | 0 | Mutant | Non-codel | low | 1 |
| CGGA_1162 | -0.501790372 | -0.172223716 | -0.329566656 | Primary | AOA | WHO III | Male | 39 | 2457 | 0 | 1 | 1 | NA | Codel | low | 1 |
| CGGA_1164 | 0.049890951 | 0.114446893 | -0.064555942 | Recurrent | rGBM | WHO IV | Female | 38 | 1022 | 1 | 0 | 1 | Mutant | Codel | high | 0 |
| CGGA_1169 | -0.367010453 | 0.110424968 | -0.477435421 | Primary | AOA | WHO III | Male | 30 | 1765 | 1 | 1 | 1 | Mutant | Non-codel | low | 1 |
| CGGA_1172 | 0.147117867 | -0.337478885 | 0.484596752 | Primary | GBM | WHO IV | Female | 36 | 2414 | 0 | 1 | 1 | Mutant | Non-codel | high | 0 |
| CGGA_1178 | 0.060207756 | 0.186523675 | -0.126315919 | Recurrent | rAOA | WHO III | Male | 41 | 422 | 1 | 1 | 1 | Mutant | Non-codel | low | 1 |
| CGGA_1181 | -0.287916168 | -0.375727581 | 0.087811413 | Primary | OA | WHO II | Male | 36 | 318 | 0 | 1 | 1 | Mutant | Non-codel | high | 1 |
| CGGA_1183 | -0.355272988 | -0.417887231 | 0.062614243 | Primary | AA | WHO III | Female | 25 | 408 | 1 | 1 | 1 | Wildtype | Non-codel | high | 1 |
| CGGA_1184 | -0.200363781 | 0.260406177 | -0.460769958 | Recurrent | rAOA | WHO III | Male | 46 | 156 | 1 | 1 | 1 | Mutant | Non-codel | low | 1 |
| CGGA_1185 | 0.005542431 | 0.222443301 | -0.21690087 | Recurrent | rAOA | WHO III | Female | 50 | 731 | 1 | 1 | 1 | Wildtype | Non-codel | low | 1 |
| CGGA_1191 | -0.379087376 | -0.033913202 | -0.345174174 | Primary | AOA | WHO III | Male | 53 | 2365 | 0 | 0 | 0 | Mutant | Codel | low | 1 |
| CGGA_1192 | -0.706685423 | -0.128776481 | -0.577908942 | Primary | OA | WHO II | Female | 43 | 2363 | 0 | 1 | 1 | Mutant | Non-codel | low | 1 |
| CGGA_1195 | -0.794487755 | -0.414630925 | -0.37985683 | Primary | AOA | WHO III | Male | 43 | NA | NA | 0 | 0 | Mutant | Non-codel | low | 1 |
| CGGA_1198 | -0.176742838 | 0.202224701 | -0.378967539 | Primary | O | WHO II | Male | 30 | 2349 | 0 | 0 | 1 | Mutant | NA | low | 1 |
| CGGA_120 | 0.255181918 | -0.189859604 | 0.445041522 | Recurrent | rGBM | WHO IV | Male | 42 | 288 | 1 | 1 | 1 | Wildtype | Non-codel | high | 0 |
| CGGA_1204 | 0.029302137 | 0.26624822 | -0.236946083 | Primary | O | WHO II | Male | 53 | 2338 | 0 | 0 | 1 | Mutant | Codel | low | 1 |
| CGGA_1205 | -0.222887121 | 0.195666036 | -0.418553157 | Primary | AOA | WHO III | Female | 59 | 929 | 1 | 1 | 1 | Wildtype | NA | low | 1 |
| CGGA_1207 | -0.142102541 | 0.265930967 | -0.408033508 | Primary | AOA | WHO III | Female | 49 | 2331 | 0 | 0 | 1 | NA | Codel | low | 1 |
| CGGA_1208 | 0.129777495 | -0.32763182 | 0.457409315 | Recurrent | rGBM | WHO IV | Male | 38 | 161 | 1 | 1 | 1 | Wildtype | Non-codel | high | 0 |
| CGGA_1211 | -0.415559773 | 0.133466872 | -0.549026645 | Primary | OA | WHO II | Male | 40 | 2324 | 0 | 0 | 0 | Mutant | Codel | low | 1 |
| CGGA_1212 | -0.620924861 | -0.061476044 | -0.559448817 | Primary | AOA | WHO III | Male | 31 | 2292 | 1 | 1 | 1 | Mutant | Non-codel | low | 1 |
| CGGA_1223 | 0.349672466 | -0.165996586 | 0.515669052 | Primary | AOA | WHO III | Male | 42 | 682 | 1 | 1 | 1 | Wildtype | Non-codel | high | 1 |
| CGGA_1226 | -0.530517796 | 0.014555556 | -0.545073352 | Primary | A | WHO II | Female | 33 | 670 | 1 | 1 | 1 | Wildtype | Non-codel | low | 1 |
| CGGA_1228 | -0.166860649 | 0.449147991 | -0.61600864 | Primary | OA | WHO II | Male | 34 | 2296 | 0 | 0 | 1 | Wildtype | Non-codel | low | 1 |
| CGGA_1232 | -0.646727908 | -0.300235705 | -0.346492203 | Primary | AOA | WHO III | Male | 39 | 2067 | 1 | 1 | 1 | Mutant | Non-codel | low | 1 |
| CGGA_1235 | -0.519924736 | 0.089420663 | -0.609345399 | Primary | AO | WHO III | Female | 32 | 2287 | 0 | 0 | 1 | Mutant | Non-codel | low | 1 |
| CGGA_1236 | -0.471786248 | 0.126651095 | -0.598437343 | Primary | GBM | WHO IV | Female | 47 | 191 | 1 | 0 | 0 | Wildtype | Non-codel | low | 0 |
| CGGA_1238 | -0.291306737 | 0.301341273 | -0.59264801 | Primary | OA | WHO II | Female | 11 | 2282 | 0 | 0 | 0 | Wildtype | Non-codel | low | 1 |
| CGGA_1239 | -0.300341943 | 0.137002429 | -0.437344372 | Primary | O | WHO II | Female | 44 | 2280 | 0 | 1 | 1 | Mutant | Codel | low | 1 |
| CGGA_1248 | 0.201832579 | 0.31250863 | -0.110676051 | Recurrent | rGBM | WHO IV | Male | 54 | 138 | 1 | 1 | 1 | Wildtype | Non-codel | low | 0 |
| CGGA_1255 | -0.330882706 | -0.38140873 | 0.050526024 | Recurrent | rGBM | WHO IV | Male | 49 | 132 | 1 | 1 | 1 | Wildtype | Non-codel | high | 0 |
| CGGA_1256 | -0.364404926 | -0.220503386 | -0.14390154 | Primary | GBM | WHO IV | Female | 52 | 2233 | 0 | 1 | 1 | NA | Non-codel | low | 0 |
| CGGA_1257 | -0.102883583 | 0.394787286 | -0.497670869 | Recurrent | rGBM | WHO IV | Male | 59 | 192 | 1 | 1 | 1 | Wildtype | Non-codel | low | 0 |
| CGGA_1260 | -0.516923107 | -0.247417061 | -0.269506046 | Recurrent | rGBM | WHO IV | Male | 32 | 177 | 1 | 1 | 1 | Wildtype | Non-codel | low | 0 |
| CGGA_1262 | -0.25559606 | 0.150155332 | -0.405751392 | Recurrent | rGBM | WHO IV | Female | 69 | 166 | 1 | 1 | 0 | Wildtype | NA | low | 0 |
| CGGA_1264 | -0.311084465 | 0.221699082 | -0.532783547 | Primary | AO | WHO III | Female | 37 | 2217 | 0 | 0 | 1 | Mutant | Codel | low | 1 |
| CGGA_1269 | -0.148221397 | 0.185468374 | -0.333689771 | Primary | AO | WHO III | Female | 35 | 2214 | 0 | 0 | 1 | Mutant | Codel | low | 1 |
| CGGA_1273 | -0.268325376 | 0.193543557 | -0.461868933 | Recurrent | rAOA | WHO III | Male | 64 | 1912 | 0 | 1 | 1 | Mutant | Non-codel | low | 1 |
| CGGA_1282 | -0.441674726 | -0.297955576 | -0.14371915 | Primary | GBM | WHO IV | Female | 33 | 1116 | 1 | 1 | 1 | Wildtype | Non-codel | low | 0 |
| CGGA_1286 | -0.3430602 | -0.054134689 | -0.288925511 | Primary | O | WHO II | Female | 52 | 2194 | 0 | 1 | 1 | Mutant | Codel | low | 1 |
| CGGA_1291 | -0.643536959 | -0.244506832 | -0.399030127 | Primary | AO | WHO III | Male | 41 | 2190 | 0 | 1 | 1 | NA | Codel | low | 1 |
| CGGA_1295 | -0.050286455 | -0.413637351 | 0.363350896 | Recurrent | rAOA | WHO III | Male | 40 | 310 | 1 | 1 | 1 | Mutant | Non-codel | high | 1 |
| CGGA_1300 | -0.443950143 | -0.106418655 | -0.337531488 | Primary | AOA | WHO III | Male | 35 | 1428 | 1 | 1 | 1 | Mutant | Non-codel | low | 1 |
| CGGA_1303 | -0.093026654 | 0.448632444 | -0.541659098 | Primary | AOA | WHO III | Female | 45 | 2168 | 0 | 1 | 1 | Mutant | NA | low | 1 |
| CGGA_1305 | -0.595666122 | -0.39408882 | -0.201577302 | Primary | AOA | WHO III | Male | 43 | 1989 | 0 | 1 | 1 | Mutant | Non-codel | low | 1 |
| CGGA_1309 | -0.296958818 | -0.28813282 | -0.008825998 | Primary | AO | WHO III | Male | 65 | 964 | 0 | 1 | 1 | NA | Codel | high | 1 |
| CGGA_1311 | 0.26698628 | 0.147365074 | 0.119621206 | Recurrent | rAOA | WHO III | Female | 29 | 1080 | 1 | 1 | 1 | Mutant | Codel | high | 1 |
| CGGA_1317 | -0.229437936 | 0.313560194 | -0.54299813 | Primary | OA | WHO II | Male | 45 | 2152 | 0 | 0 | 1 | Mutant | NA | low | 1 |
| CGGA_1318 | -0.703588382 | -0.233469903 | -0.470118479 | Primary | OA | WHO II | Male | 39 | 1497 | 0 | 1 | 0 | Mutant | Non-codel | low | 1 |
| CGGA_1319 | -0.518591321 | -0.287447814 | -0.231143507 | Primary | AO | WHO III | Female | 42 | 2149 | 0 | 1 | 1 | Wildtype | Non-codel | low | 1 |
| CGGA_1321 | 0.170043752 | -0.209793204 | 0.379836956 | Primary | AOA | WHO III | Male | 46 | 702 | 1 | 1 | 1 | Mutant | Non-codel | high | 1 |
| CGGA_1325 | -0.104660904 | 0.279986403 | -0.384647307 | Recurrent | rGBM | WHO IV | Male | 20 | NA | NA | 0 | NA | Wildtype | NA | low | 0 |
| CGGA_1326 | 0.263258925 | -0.221184159 | 0.484443084 | Primary | GBM | WHO IV | Male | 45 | 322 | 1 | 1 | 0 | Mutant | Non-codel | high | 0 |
| CGGA_1334 | -0.347193757 | 0.291416373 | -0.63861013 | Primary | OA | WHO II | Female | 33 | 146 | 1 | 1 | 1 | Wildtype | Non-codel | low | 1 |
| CGGA_1335 | -0.098594478 | 0.145554889 | -0.244149367 | Primary | O | WHO II | Female | 43 | 1947 | 0 | 0 | 1 | Mutant | Codel | low | 1 |
| CGGA_1337 | -0.390721612 | 0.08958004 | -0.480301652 | Recurrent | rGBM | WHO IV | Male | 67 | 2126 | 0 | 1 | 1 | NA | Non-codel | low | 0 |
| CGGA_1339 | -0.298749958 | 0.164495068 | -0.463245026 | Primary | O | WHO II | Male | 41 | 2120 | 0 | 0 | 1 | Mutant | Codel | low | 1 |
| CGGA_1345 | -0.456442025 | -0.125784872 | -0.330657153 | Primary | OA | WHO II | Male | 56 | 2113 | 0 | 1 | 0 | Mutant | Codel | low | 1 |
| CGGA_135 | 0.456566946 | 0.120245052 | 0.336321894 | Primary | AA | WHO III | Female | 41 | 1924 | 1 | 1 | 0 | Wildtype | Non-codel | high | 1 |
| CGGA_1350 | -0.244134316 | 0.260609571 | -0.504743887 | Primary | O | WHO II | Male | 36 | 2107 | 0 | 0 | 1 | Mutant | NA | low | 1 |
| CGGA_1353 | 0.111851878 | -0.313561516 | 0.425413394 | Primary | GBM | WHO IV | Male | 65 | 1022 | 1 | 1 | 1 | Wildtype | Non-codel | high | 0 |
| CGGA_1354 | 0.579921039 | -0.126162925 | 0.706083964 | Primary | GBM | WHO IV | Female | 40 | 530 | 1 | 1 | 1 | Wildtype | Non-codel | high | 0 |
| CGGA_1356 | -0.094136339 | 0.408328535 | -0.502464874 | Recurrent | rAOA | WHO III | Male | 52 | 948 | 1 | 0 | 1 | Mutant | Non-codel | low | 1 |
| CGGA_1359 | -0.001250867 | 0.017012663 | -0.01826353 | Recurrent | rAOA | WHO III | Male | 21 | 343 | 1 | 1 | 1 | Wildtype | Non-codel | high | 1 |
| CGGA_1361 | -0.04216898 | 0.401200769 | -0.443369749 | Primary | O | WHO II | Male | 40 | 2092 | 0 | 1 | 1 | Mutant | Non-codel | low | 1 |
| CGGA_1362 | -0.030305 | 0.351848467 | -0.382153467 | Primary | AA | WHO III | Female | 64 | 2092 | 0 | 1 | 1 | Wildtype | Non-codel | low | 1 |
| CGGA_1365 | 0.086180518 | 0.047052634 | 0.039127884 | Primary | GBM | WHO IV | Male | 55 | 253 | 1 | 1 | 1 | Wildtype | NA | high | 0 |
| CGGA_1368 | -0.237226735 | 0.304440906 | -0.541667641 | Primary | O | WHO II | Male | 49 | 2085 | 0 | 1 | 0 | Mutant | NA | low | 1 |
| CGGA_1369 | -0.074547633 | -0.222230063 | 0.14768243 | Primary | AO | WHO III | Female | 37 | 360 | 0 | 1 | 1 | Mutant | Non-codel | high | 1 |
| CGGA_1371 | -0.208878023 | -0.483541658 | 0.274663635 | Primary | GBM | WHO IV | Male | 68 | 2079 | 0 | 1 | 1 | Wildtype | Non-codel | high | 0 |
| CGGA_1377 | -0.432357873 | -0.140288351 | -0.292069522 | Primary | AOA | WHO III | Male | 28 | 2068 | 0 | 1 | 1 | Mutant | Non-codel | low | 1 |
| CGGA_1378 | 0.066753653 | 0.035089371 | 0.031664282 | Primary | GBM | WHO IV | Male | 47 | 585 | 1 | 1 | 1 | Wildtype | NA | high | 0 |
| CGGA_1380 | 0.166543343 | 0.345638246 | -0.179094903 | Primary | GBM | WHO IV | Male | 46 | 291 | 1 | 1 | 1 | Wildtype | Non-codel | low | 0 |
| CGGA_1382 | -0.198477468 | -0.172516717 | -0.025960751 | Primary | GBM | WHO IV | Male | 57 | 284 | 1 | 1 | 1 | Wildtype | Non-codel | high | 0 |
| CGGA_1383 | -0.154171633 | -0.368933113 | 0.21476148 | Recurrent | rAA | WHO III | Female | 47 | 884 | 0 | 1 | NA | Mutant | Codel | high | 1 |
| CGGA_1386 | -0.381154356 | -0.290796587 | -0.090357769 | Primary | AO | WHO III | Male | 34 | 2058 | 0 | 1 | 1 | NA | Codel | high | 1 |
| CGGA_1387 | 0.602152867 | 0.031266342 | 0.570886525 | Recurrent | rGBM | WHO IV | Male | 41 | 90 | 1 | 1 | 1 | Wildtype | Non-codel | high | 0 |
| CGGA_1389 | -0.435359692 | -0.132384485 | -0.302975207 | Primary | AOA | WHO III | Female | 67 | 1370 | 1 | 1 | 0 | Mutant | Codel | low | 1 |
| CGGA_139 | -0.088840813 | 0.438647667 | -0.52748848 | Primary | GBM | WHO IV | Male | 59 | 694 | 1 | 1 | 1 | Mutant | Non-codel | low | 0 |
| CGGA_1390 | -0.134420728 | 0.264499704 | -0.398920432 | Recurrent | rAOA | WHO III | Male | 50 | 2050 | 0 | 1 | 1 | Mutant | Non-codel | low | 1 |
| CGGA_1391 | -0.03567526 | -0.448229591 | 0.412554331 | Primary | GBM | WHO IV | Male | 62 | 426 | 1 | 1 | 1 | Wildtype | Non-codel | high | 0 |
| CGGA_1392 | 0.038617016 | -0.423557103 | 0.462174119 | Primary | GBM | WHO IV | Male | 62 | 473 | 1 | 1 | 1 | Wildtype | Non-codel | high | 0 |
| CGGA_1398 | 0.060990981 | 0.051035574 | 0.009955407 | Recurrent | rAOA | WHO III | Male | 43 | 554 | 1 | 1 | 1 | Mutant | Non-codel | high | 1 |
| CGGA_1400 | -0.570544595 | -0.281280687 | -0.289263908 | Recurrent | rAO | WHO III | Female | 53 | 2033 | 0 | 1 | 1 | NA | Codel | low | 1 |
| CGGA_1401 | -0.538029237 | 0.008795736 | -0.546824973 | Primary | AA | WHO III | Male | 39 | 1680 | 1 | 1 | 1 | Mutant | Non-codel | low | 1 |
| CGGA_1402 | 0.324591408 | -0.26254123 | 0.587132638 | Primary | GBM | WHO IV | Male | 30 | 2030 | 0 | 1 | 1 | Wildtype | Non-codel | high | 0 |
| CGGA_1403 | 0.399613917 | 0.068654481 | 0.330959436 | Primary | GBM | WHO IV | Female | 43 | 679 | 1 | 1 | 1 | Wildtype | Non-codel | high | 0 |
| CGGA_1404 | 0.240024432 | 0.135399319 | 0.104625113 | Primary | AOA | WHO III | Female | 56 | 2029 | 0 | 1 | 0 | Mutant | Codel | high | 1 |
| CGGA_1407 | -0.370669453 | -0.102827398 | -0.267842055 | Primary | AO | WHO III | Female | 29 | 2016 | 0 | 0 | 1 | Mutant | Codel | low | 1 |
| CGGA_1410 | 0.411250145 | 0.04659156 | 0.364658585 | Primary | GBM | WHO IV | Female | 27 | 825 | 1 | 1 | 1 | Wildtype | Non-codel | high | 0 |
| CGGA_1413 | -0.048224815 | 0.535249907 | -0.583474722 | Primary | AO | WHO III | Male | 35 | 2008 | 0 | 1 | 1 | Mutant | Codel | low | 1 |
| CGGA_1415 | -0.081591836 | -0.216619686 | 0.13502785 | Recurrent | rGBM | WHO IV | Female | 30 | 2007 | 0 | 1 | 1 | Wildtype | Non-codel | high | 0 |
| CGGA_1416 | 0.292436642 | -0.109238921 | 0.401675563 | Primary | AO | WHO III | Male | 42 | NA | NA | 0 | 0 | Mutant | Codel | high | 1 |
| CGGA_1417 | -0.019586791 | 0.390427909 | -0.4100147 | Primary | AOA | WHO III | Male | 56 | 2001 | 0 | 1 | 1 | Mutant | Codel | low | 1 |
| CGGA_1418 | -0.011186386 | 0.085784165 | -0.096970551 | Primary | GBM | WHO IV | Female | 73 | 287 | 1 | 1 | 1 | Wildtype | NA | high | 0 |
| CGGA_1419 | -0.429123484 | 0.127430391 | -0.556553875 | Recurrent | rGBM | WHO IV | Male | 56 | 249 | 1 | 1 | 1 | Wildtype | Non-codel | low | 0 |
| CGGA_1420 | -0.331278556 | 0.133268683 | -0.464547239 | Primary | GBM | WHO IV | Male | 60 | 364 | 1 | 1 | 1 | Wildtype | Non-codel | low | 0 |
| CGGA_1421 | -0.207900759 | 0.154945866 | -0.362846625 | Primary | AO | WHO III | Male | 35 | 1987 | 0 | 1 | 1 | NA | Non-codel | low | 1 |
| CGGA_1422 | -0.312848174 | -0.158692969 | -0.154155205 | Primary | GBM | WHO IV | Male | 76 | 204 | 1 | 1 | 1 | Wildtype | NA | low | 0 |
| CGGA_1424 | -0.323913388 | 0.124310448 | -0.448223836 | Primary | AO | WHO III | Female | 41 | 1983 | 0 | 1 | 1 | Mutant | NA | low | 1 |
| CGGA_1425 | 0.197026685 | -0.187677712 | 0.384704397 | Primary | GBM | WHO IV | Female | 22 | 640 | 1 | 1 | 1 | Wildtype | Codel | high | 0 |
| CGGA_1426 | -0.105190096 | 0.084276089 | -0.189466185 | Primary | GBM | WHO IV | Female | 50 | 147 | 1 | 1 | 1 | Wildtype | Non-codel | low | 0 |
| CGGA_1427 | -0.5473348 | 0.035387958 | -0.582722758 | Primary | OA | WHO II | Female | 30 | NA | NA | 1 | 0 | NA | Non-codel | low | 1 |
| CGGA_1429 | 0.439673695 | 0.269090288 | 0.170583407 | Recurrent | rGBM | WHO IV | Female | 40 | 1976 | 0 | 1 | 1 | Mutant | Codel | high | 0 |
| CGGA_1430 | 0.5458018 | -0.05008743 | 0.59588923 | Recurrent | rGBM | WHO IV | Female | 28 | 176 | 1 | 1 | 1 | Wildtype | Non-codel | high | 0 |
| CGGA_1431 | -0.522828861 | -0.181664523 | -0.341164338 | Primary | AA | WHO III | Male | 48 | 1975 | 0 | 1 | 1 | Mutant | Non-codel | low | 1 |
| CGGA_1433 | 0.305438389 | -0.24160374 | 0.547042129 | Primary | GBM | WHO IV | Female | 72 | 487 | 1 | 1 | 1 | Wildtype | Non-codel | high | 0 |
| CGGA_1434 | -0.04855542 | -0.046257292 | -0.002298128 | Recurrent | rAA | WHO III | Male | 61 | 81 | 1 | 1 | 1 | Wildtype | Non-codel | high | 1 |
| CGGA_1435 | -0.17328343 | -0.266796431 | 0.093513001 | Primary | AOA | WHO III | Female | 46 | 324 | 0 | 1 | 0 | Mutant | Non-codel | high | 1 |
| CGGA_1437 | -0.233774266 | 0.32481807 | -0.558592336 | Recurrent | rAOA | WHO III | Male | 48 | 1037 | 1 | 1 | 1 | Wildtype | Non-codel | low | 1 |
| CGGA_1440 | -0.160137612 | 0.373882753 | -0.534020365 | Primary | O | WHO II | Female | 29 | 1961 | 0 | 0 | 1 | Mutant | Codel | low | 1 |
| CGGA_1441 | 0.170811844 | -0.213909963 | 0.384721807 | Primary | GBM | WHO IV | Male | 70 | 1340 | 1 | 1 | 1 | Wildtype | Non-codel | high | 0 |
| CGGA_1443 | -0.283104278 | -0.219210976 | -0.063893302 | Recurrent | rOA | WHO II | Male | 41 | 1956 | 0 | 1 | NA | Wildtype | Non-codel | high | 1 |
| CGGA_1444 | 0.487180575 | -0.276264886 | 0.763445461 | Primary | GBM | WHO IV | Female | 68 | 476 | 1 | 1 | 1 | Wildtype | Non-codel | high | 0 |
| CGGA_1445 | 0.230372242 | 0.063994495 | 0.166377747 | Recurrent | rAOA | WHO III | Male | 37 | 1955 | 0 | 1 | 1 | Wildtype | NA | high | 1 |
| CGGA_1446 | -0.274696512 | 0.182703832 | -0.457400344 | Primary | OA | WHO II | Male | 29 | 1955 | 0 | 0 | 1 | Mutant | NA | low | 1 |
| CGGA_1447 | -0.022695789 | 0.316770921 | -0.33946671 | Recurrent | rAOA | WHO III | Male | 48 | 308 | 0 | 1 | 1 | Wildtype | NA | low | 1 |
| CGGA_1451 | 0.161718286 | -0.451568155 | 0.613286441 | Primary | GBM | WHO IV | Female | 45 | 438 | 1 | 1 | 1 | Wildtype | Non-codel | high | 0 |
| CGGA_1452 | 0.183242024 | -0.441208886 | 0.62445091 | Primary | GBM | WHO IV | Male | 53 | 468 | 1 | 1 | 1 | Wildtype | Non-codel | high | 0 |
| CGGA_1454 | -0.153941138 | 0.328822491 | -0.482763629 | Primary | OA | WHO II | Female | 42 | 1946 | 0 | 1 | 0 | Wildtype | Non-codel | low | 1 |
| CGGA_1455 | -0.221297207 | 0.222682305 | -0.443979512 | Primary | O | WHO II | Male | 40 | 1946 | 0 | 0 | 0 | Mutant | NA | low | 1 |
| CGGA_1457 | 0.223491539 | -0.30894123 | 0.532432769 | Primary | GBM | WHO IV | Male | 60 | 312 | 1 | 1 | 1 | Wildtype | Non-codel | high | 0 |
| CGGA_1458 | -0.020895518 | 0.017016276 | -0.037911794 | Recurrent | rAOA | WHO III | Female | 54 | 127 | 1 | 1 | 1 | Mutant | Codel | high | 1 |
| CGGA_1459 | 0.392744996 | -0.14505369 | 0.537798686 | Recurrent | rAOA | WHO III | Male | 55 | 522 | 0 | 1 | 1 | Wildtype | Non-codel | high | 1 |
| CGGA_1461 | 0.413692696 | -0.184138061 | 0.597830757 | Primary | GBM | WHO IV | Female | 60 | 226 | 1 | 1 | 1 | Wildtype | Non-codel | high | 0 |
| CGGA_1462 | 0.582347661 | -0.007218269 | 0.58956593 | Primary | GBM | WHO IV | Male | 49 | 188 | 1 | 1 | 1 | Wildtype | Non-codel | high | 0 |
| CGGA_1463 | 0.028965128 | 0.375822674 | -0.346857546 | Primary | O | WHO II | Female | 69 | 1630 | 0 | 1 | 0 | Mutant | Codel | low | 1 |
| CGGA_1467 | -0.184382208 | 0.073276183 | -0.257658391 | Primary | GBM | WHO IV | Male | 58 | 866 | 1 | 1 | 0 | Mutant | NA | low | 0 |
| CGGA_1469 | -0.305627206 | 0.11270002 | -0.418327226 | Primary | AOA | WHO III | Male | 46 | 1922 | 0 | 1 | 1 | Mutant | NA | low | 1 |
| CGGA_1471 | -0.879328246 | -0.423235514 | -0.456092732 | Primary | OA | WHO II | Male | 45 | 1919 | 0 | 1 | 1 | Mutant | Non-codel | low | 1 |
| CGGA_1472 | -0.482734303 | -0.046397767 | -0.436336536 | Primary | GBM | WHO IV | Male | 34 | 819 | 0 | 0 | 0 | NA | Non-codel | low | 0 |
| CGGA_1473 | -0.412145998 | 0.152413159 | -0.564559157 | Recurrent | rAA | WHO III | Female | 28 | 813 | 0 | 1 | 1 | NA | Non-codel | low | 1 |
| CGGA_1474 | -0.188041944 | 0.253488335 | -0.441530279 | Primary | AO | WHO III | Female | 50 | 333 | 0 | 1 | 1 | Mutant | NA | low | 1 |
| CGGA_1476 | -0.054768815 | -0.330476881 | 0.275708066 | Primary | GBM | WHO IV | Female | 53 | 299 | 1 | 0 | 0 | Wildtype | Non-codel | high | 0 |
| CGGA_1477 | -0.639650568 | -0.270230273 | -0.369420295 | Primary | OA | WHO II | Male | 43 | 1194 | 1 | 1 | 1 | NA | Non-codel | low | 1 |
| CGGA_1478 | 0.002109654 | -0.328943717 | 0.331053371 | Primary | GBM | WHO IV | Female | 72 | 542 | 1 | 1 | 1 | Wildtype | Non-codel | high | 0 |
| CGGA_1480 | -0.28348374 | -0.408260894 | 0.124777154 | Recurrent | rGBM | WHO IV | Male | 41 | NA | NA | 1 | 1 | Wildtype | Non-codel | high | 0 |
| CGGA_1481 | -0.41074339 | -0.156637259 | -0.254106131 | Primary | GBM | WHO IV | Male | 55 | 131 | 1 | 0 | 0 | Wildtype | NA | low | 0 |
| CGGA_1482 | -0.36173586 | -0.313701622 | -0.048034238 | Primary | AO | WHO III | Male | 38 | 1888 | 0 | 1 | 1 | NA | Codel | high | 1 |
| CGGA_1486 | -0.378671092 | -0.15452371 | -0.224147382 | Primary | GBM | WHO IV | Male | 45 | 184 | 1 | 1 | 1 | Wildtype | NA | low | 0 |
| CGGA_1487 | 0.754936811 | 0.173845027 | 0.581091784 | Recurrent | rAA | WHO III | Female | 49 | 520 | 1 | 1 | 1 | Wildtype | Non-codel | high | 1 |
| CGGA_1488 | -0.075677724 | 0.438652397 | -0.514330121 | Primary | OA | WHO II | Male | 31 | 1882 | 0 | 0 | 0 | Mutant | Non-codel | low | 1 |
| CGGA_1491 | 0.37213476 | -0.318333555 | 0.690468315 | Primary | GBM | WHO IV | Male | 29 | 511 | 1 | 1 | 1 | Mutant | Non-codel | high | 0 |
| CGGA_1492 | 0.189691373 | 0.094431185 | 0.095260188 | Recurrent | rGBM | WHO IV | Female | 47 | 268 | 1 | 1 | 1 | Wildtype | Non-codel | high | 0 |
| CGGA_1494 | -0.443181348 | -0.037182725 | -0.405998623 | Primary | GBM | WHO IV | Male | 21 | 269 | 1 | 1 | 1 | Wildtype | NA | low | 0 |
| CGGA_1496 | 0.026208991 | 0.145428549 | -0.119219558 | Recurrent | rGBM | WHO IV | Female | 42 | 330 | 1 | 1 | 1 | Mutant | Codel | low | 0 |
| CGGA_1497 | 0.253942422 | -0.260026892 | 0.513969314 | Primary | AOA | WHO III | Female | 24 | 1178 | 1 | 1 | 1 | Mutant | Non-codel | high | 1 |
| CGGA_1498 | -0.263451686 | 0.103747261 | -0.367198947 | Primary | GBM | WHO IV | Male | 69 | NA | NA | NA | NA | Wildtype | NA | low | 0 |
| CGGA_1500 | 0.350950098 | 0.358911091 | -0.007960993 | Primary | GBM | WHO IV | Female | 45 | 108 | 1 | 1 | 1 | Wildtype | Non-codel | high | 0 |
| CGGA_1501 | 0.185698132 | -0.054926861 | 0.240624993 | Primary | GBM | WHO IV | Male | 58 | 222 | 1 | 1 | 1 | NA | Non-codel | high | 0 |
| CGGA_1502 | 0.112441276 | -0.125786997 | 0.238228273 | Primary | OA | WHO II | Female | 27 | 1861 | 0 | 0 | 0 | Mutant | Codel | high | 1 |
| CGGA_1503 | -0.200007095 | 0.155567413 | -0.355574508 | Primary | GBM | WHO IV | Male | 47 | 692 | 0 | 1 | 1 | Wildtype | NA | low | 0 |
| CGGA_1504 | -0.598643777 | -0.305226144 | -0.293417633 | Primary | AOA | WHO III | Female | 44 | 1854 | 0 | 1 | 1 | Mutant | Non-codel | low | 1 |
| CGGA_1505 | 0.200329925 | 0.187810443 | 0.012519482 | Recurrent | rGBM | WHO IV | Male | 30 | 172 | 1 | 1 | 1 | Mutant | Non-codel | high | 0 |
| CGGA_1507 | -0.428680445 | -0.367137276 | -0.061543169 | Recurrent | rGBM | WHO IV | Female | 41 | 715 | 1 | 1 | 1 | Wildtype | Non-codel | high | 0 |
| CGGA_1508 | -0.556851037 | -0.244224647 | -0.31262639 | Primary | AOA | WHO III | Male | 39 | 1049 | 1 | 0 | 0 | Mutant | Non-codel | low | 1 |
| CGGA_1510 | 0.000173809 | -0.362166611 | 0.36234042 | Recurrent | rAOA | WHO III | Male | 40 | 465 | 1 | 1 | 1 | Mutant | Non-codel | high | 1 |
| CGGA_1513 | -0.244536076 | -0.235742158 | -0.008793918 | Recurrent | rAOA | WHO III | Female | 36 | 1827 | 0 | 1 | 1 | Mutant | Codel | high | 1 |
| CGGA_1514 | 0.079773563 | 0.337742811 | -0.257969248 | Primary | OA | WHO II | Female | 57 | 76 | 1 | 0 | 0 | Wildtype | NA | low | 1 |
| CGGA_1516 | -0.394589034 | 0.029623049 | -0.424212083 | Primary | OA | WHO II | Male | 46 | 1826 | 0 | 1 | 1 | Mutant | Codel | low | 1 |
| CGGA_1517 | -0.193846194 | 0.211823975 | -0.405670169 | Primary | O | WHO II | Male | 42 | 1826 | 0 | 1 | 0 | Mutant | Codel | low | 1 |
| CGGA_1518 | 0.299017506 | 0.418740116 | -0.11972261 | Primary | AOA | WHO III | Female | 19 | 411 | 1 | 1 | 1 | Wildtype | Non-codel | low | 1 |
| CGGA_1520 | -0.406218446 | 0.032613025 | -0.438831471 | Recurrent | rGBM | WHO IV | Female | 67 | 297 | 1 | 1 | 1 | Wildtype | Non-codel | low | 0 |
| CGGA_1521 | -0.129994517 | -0.05289608 | -0.077098437 | Primary | GBM | WHO IV | Female | 63 | 205 | 1 | 1 | 1 | Wildtype | Non-codel | high | 0 |
| CGGA_1523 | -0.251222817 | 0.307036565 | -0.558259382 | Primary | OA | WHO II | Male | 50 | 1804 | 0 | 1 | 1 | Mutant | Non-codel | low | 1 |
| CGGA_1524 | 0.381436744 | 0.085934168 | 0.295502576 | Recurrent | rAOA | WHO III | Male | 36 | 1226 | 0 | 1 | 1 | Mutant | Codel | high | 1 |
| CGGA_1525 | -0.573816266 | -0.250852333 | -0.322963933 | Primary | AOA | WHO III | Male | 41 | 1686 | 1 | 1 | 1 | NA | Non-codel | low | 1 |
| CGGA_1526 | 0.192474462 | 0.367293898 | -0.174819436 | Primary | AOA | WHO III | Male | 39 | 1798 | 0 | 1 | 1 | Mutant | Codel | low | 1 |
| CGGA_1527 | -0.393960556 | -0.205518588 | -0.188441968 | Primary | AOA | WHO III | Male | 47 | 1714 | 1 | 1 | 1 | Mutant | Non-codel | low | 1 |
| CGGA_1528 | -0.523620905 | -0.107779829 | -0.415841076 | Primary | O | WHO II | Female | 27 | 1793 | 0 | 1 | 0 | Mutant | Codel | low | 1 |
| CGGA_1529 | 0.696572929 | 0.169231841 | 0.527341088 | Primary | GBM | WHO IV | Male | 63 | NA | NA | NA | NA | Wildtype | Non-codel | high | 0 |
| CGGA_1530 | -0.55895822 | -0.181845452 | -0.377112768 | Primary | AOA | WHO III | Male | 41 | 1793 | 0 | 1 | 1 | NA | Non-codel | low | 1 |
| CGGA_1531 | -0.59544511 | -0.092922979 | -0.502522131 | Primary | AOA | WHO III | Male | 36 | NA | NA | NA | NA | Mutant | Non-codel | low | 1 |
| CGGA_1533 | -0.22720508 | -0.19813605 | -0.02906903 | Recurrent | rAOA | WHO III | Female | 61 | 1217 | 1 | 1 | 1 | Wildtype | Non-codel | high | 1 |
| CGGA_1534 | -0.232748365 | -0.245393815 | 0.01264545 | Primary | GBM | WHO IV | Female | 58 | 41 | 1 | 0 | 0 | Mutant | Non-codel | high | 0 |
| CGGA_1535 | -0.486585703 | -0.283372927 | -0.203212776 | Recurrent | rGBM | WHO IV | Male | 43 | NA | NA | 1 | 1 | Mutant | Non-codel | low | 0 |
| CGGA_1536 | -0.408564889 | 0.0775458 | -0.486110689 | Primary | OA | WHO II | Male | 53 | 840 | 1 | 0 | 0 | Mutant | Non-codel | low | 1 |
| CGGA_1537 | 0.264549763 | -0.270423547 | 0.53497331 | Primary | GBM | WHO IV | Male | 73 | 200 | 0 | 0 | 0 | Wildtype | Non-codel | high | 0 |
| CGGA_1538 | 0.053008746 | -0.412005192 | 0.465013938 | Recurrent | rGBM | WHO IV | Female | 57 | 247 | 1 | 1 | 1 | Wildtype | Non-codel | high | 0 |
| CGGA_1539 | -0.408761043 | 0.234449019 | -0.643210062 | Primary | GBM | WHO IV | Male | 61 | 1758 | 0 | 1 | 1 | Mutant | Non-codel | low | 0 |
| CGGA_1541 | 0.520451505 | 0.036056406 | 0.484395099 | Recurrent | rGBM | WHO IV | Female | 50 | 81 | 1 | 1 | 1 | Wildtype | Non-codel | high | 0 |
| CGGA_1542 | -0.411448131 | 0.024089768 | -0.435537899 | Primary | GBM | WHO IV | Male | 26 | 184 | 0 | 1 | 1 | Mutant | NA | low | 0 |
| CGGA_1543 | 0.029434881 | -0.317125245 | 0.346560126 | Primary | GBM | WHO IV | Male | 57 | 723 | 1 | 1 | 0 | Mutant | Non-codel | high | 0 |
| CGGA_1544 | -0.13716058 | -0.235633331 | 0.098472751 | Primary | AOA | WHO III | Female | 38 | 208 | 1 | 0 | 0 | Mutant | Non-codel | high | 1 |
| CGGA_1546 | 0.099597452 | -0.307856094 | 0.407453546 | Primary | GBM | WHO IV | Male | 56 | 223 | 1 | 0 | 1 | Wildtype | Non-codel | high | 0 |
| CGGA_1548 | 0.301222061 | -0.166377019 | 0.46759908 | Primary | GBM | WHO IV | Male | 53 | 1054 | 1 | 1 | 1 | Wildtype | Non-codel | high | 0 |
| CGGA_1551 | -0.175904571 | -0.218234457 | 0.042329886 | Primary | GBM | WHO IV | Female | 50 | 347 | 1 | 1 | 1 | NA | Non-codel | high | 0 |
| CGGA_1552 | -0.372581868 | -0.091356192 | -0.281225676 | Primary | AOA | WHO III | Male | 39 | 1728 | 0 | 1 | 1 | Mutant | Codel | low | 1 |
| CGGA_1553 | 0.278929916 | 0.449909959 | -0.170980043 | Recurrent | rAOA | WHO III | Male | 30 | 540 | 1 | 1 | 1 | Mutant | Non-codel | low | 1 |
| CGGA_1554 | 0.202184284 | 0.204060511 | -0.001876227 | Recurrent | rAOA | WHO III | Male | 34 | NA | NA | 1 | 1 | Mutant | Codel | high | 1 |
| CGGA_1557 | -0.15434836 | -0.071684049 | -0.082664311 | Primary | AOA | WHO III | Male | 47 | 1723 | 0 | 1 | 1 | Mutant | Codel | high | 1 |
| CGGA_1558 | 0.24032276 | -0.417468236 | 0.657790996 | Recurrent | rGBM | WHO IV | Male | 71 | 130 | 1 | 1 | 1 | Wildtype | Non-codel | high | 0 |
| CGGA_1559 | 0.265247304 | -0.221581953 | 0.486829257 | Primary | GBM | WHO IV | Male | 63 | 603 | 1 | 0 | 1 | Mutant | Codel | high | 0 |
| CGGA_1560 | 0.220019911 | -0.426064545 | 0.646084456 | Primary | GBM | WHO IV | Female | 35 | 459 | 1 | 1 | 1 | Mutant | Non-codel | high | 0 |
| CGGA_1562 | -0.421454327 | 0.064422765 | -0.485877092 | Primary | AO | WHO III | Male | 51 | 1721 | 0 | 1 | 0 | Mutant | Codel | low | 1 |
| CGGA_1563 | -0.859498374 | -0.423593153 | -0.435905221 | Primary | A | WHO II | Female | 32 | 1719 | 0 | 1 | 0 | Wildtype | Non-codel | low | 1 |
| CGGA_1564 | -0.015014013 | -0.374099527 | 0.359085514 | Primary | GBM | WHO IV | Male | 48 | 190 | 1 | 1 | 1 | Wildtype | Non-codel | high | 0 |
| CGGA_1565 | -0.630717117 | -0.252061284 | -0.378655833 | Primary | AOA | WHO III | Female | 23 | 1417 | 0 | 0 | 0 | Mutant | Non-codel | low | 1 |
| CGGA_1566 | 0.163097122 | -0.279983698 | 0.44308082 | Primary | AOA | WHO III | Female | 43 | 1716 | 0 | 1 | 1 | Mutant | Codel | high | 1 |
| CGGA_1567 | 0.030388646 | 0.113762176 | -0.08337353 | Primary | AO | WHO III | Female | 48 | 840 | 1 | 1 | 1 | Mutant | Non-codel | high | 1 |
| CGGA_1568 | -0.488973056 | -0.112571328 | -0.376401728 | Primary | AA | WHO III | Female | 26 | 345 | 0 | 0 | 0 | NA | Non-codel | low | 1 |
| CGGA_1569 | -0.467805433 | -0.215036111 | -0.252769322 | Primary | AOA | WHO III | Female | 27 | 1406 | 0 | 0 | 0 | Wildtype | Codel | low | 1 |
| CGGA_157 | -0.268191088 | -0.267004959 | -0.001186129 | Recurrent | rA | WHO II | Male | 34 | 914 | 1 | 1 | 1 | NA | Non-codel | high | 1 |
| CGGA_1571 | -0.465217554 | -0.16095663 | -0.304260924 | Primary | GBM | WHO IV | Female | 43 | 412 | 0 | 1 | 1 | Mutant | Non-codel | low | 0 |
| CGGA_1572 | 0.01880795 | -0.334659467 | 0.353467417 | Recurrent | rGBM | WHO IV | Female | 60 | 160 | 1 | 1 | 1 | Wildtype | Non-codel | high | 0 |
| CGGA_1575 | 0.329351824 | -0.306304793 | 0.635656617 | Recurrent | rAOA | WHO III | Male | 37 | 129 | 1 | 1 | 0 | Mutant | Non-codel | high | 1 |
| CGGA_1579 | -0.303982234 | 0.077862922 | -0.381845156 | Primary | OA | WHO II | Male | 37 | 1512 | 0 | 0 | 0 | Wildtype | Non-codel | low | 1 |
| CGGA_1580 | -0.35133515 | 0.099541821 | -0.450876971 | Primary | AA | WHO III | Female | 34 | 1581 | 1 | 1 | 1 | Mutant | Non-codel | low | 1 |
| CGGA_1583 | -0.407750234 | 0.16208235 | -0.569832584 | Primary | OA | WHO II | Female | 51 | 1606 | 1 | 1 | 1 | NA | Non-codel | low | 1 |
| CGGA_1586 | 0.057511777 | -0.312436384 | 0.369948161 | Primary | GBM | WHO IV | Female | 55 | 232 | 1 | 1 | 1 | Wildtype | Non-codel | high | 0 |
| CGGA_1587 | 0.318712078 | -0.252634404 | 0.571346482 | Primary | AA | WHO III | Female | 30 | 750 | 1 | 0 | 0 | Mutant | Non-codel | high | 1 |
| CGGA_1588 | -0.312058933 | -0.24937179 | -0.062687143 | Primary | AOA | WHO III | Female | 24 | 1679 | 0 | 0 | 0 | Wildtype | Non-codel | high | 1 |
| CGGA_1589 | -0.092356946 | 0.444830267 | -0.537187213 | Recurrent | rA | WHO II | Male | 49 | 342 | 1 | 1 | 1 | Wildtype | Non-codel | low | 1 |
| CGGA_1591 | -0.156067764 | 0.377138035 | -0.533205799 | Recurrent | rAOA | WHO III | Male | 54 | 1133 | 1 | 1 | 1 | Mutant | Non-codel | low | 1 |
| CGGA_1592 | 0.261654661 | 0.332963547 | -0.071308886 | Recurrent | rAOA | WHO III | Male | 37 | 1588 | 1 | 1 | 1 | Mutant | Codel | high | 1 |
| CGGA_1594 | -0.647995998 | -0.251142883 | -0.396853115 | Recurrent | rA | WHO II | Male | 25 | 480 | 0 | 0 | 1 | NA | Non-codel | low | 1 |
| CGGA_1595 | -0.041416147 | -0.430230071 | 0.388813924 | Primary | GBM | WHO IV | Female | 34 | 1659 | 0 | 1 | 1 | NA | Non-codel | high | 0 |
| CGGA_1596 | 0.092157835 | -0.343484843 | 0.435642678 | Primary | GBM | WHO IV | Male | 63 | 205 | 1 | 1 | 1 | Wildtype | Non-codel | high | 0 |
| CGGA_1597 | 0.407044791 | -0.19149125 | 0.598536041 | Primary | GBM | WHO IV | Male | 58 | 174 | 1 | 1 | 1 | Wildtype | Non-codel | high | 0 |
| CGGA_1598 | 0.484150801 | -0.163581345 | 0.647732146 | Primary | AOA | WHO III | Male | 31 | 564 | 1 | 1 | 1 | Wildtype | Non-codel | high | 1 |
| CGGA_1601 | 0.503941798 | -0.188546867 | 0.692488665 | Primary | GBM | WHO IV | Male | 66 | 710 | 1 | 1 | 1 | Wildtype | Non-codel | high | 0 |
| CGGA_1603 | 0.130882971 | -0.501950291 | 0.632833262 | Recurrent | rGBM | WHO IV | Male | 18 | 356 | 1 | 1 | 1 | Wildtype | Non-codel | high | 0 |
| CGGA_1604 | 0.50568417 | -0.152524557 | 0.658208727 | Recurrent | rGBM | WHO IV | Male | 46 | 740 | 1 | 1 | 1 | Mutant | Non-codel | high | 0 |
| CGGA_1605 | -0.264363175 | 0.230218275 | -0.49458145 | Recurrent | rGBM | WHO IV | Male | 34 | 321 | 1 | 1 | 1 | Wildtype | Non-codel | low | 0 |
| CGGA_1606 | 0.540471947 | -0.076448845 | 0.616920792 | Primary | AOA | WHO III | Female | 28 | 471 | 1 | 1 | 1 | Wildtype | Non-codel | high | 1 |
| CGGA_1607 | 0.409649451 | -0.082000095 | 0.491649546 | Recurrent | rOA | WHO II | Female | 35 | 1333 | 1 | 1 | 1 | Mutant | Codel | high | 1 |
| CGGA_1608 | 0.536188114 | 0.033108334 | 0.50307978 | Recurrent | rAA | WHO III | Male | 38 | 1629 | 0 | 1 | 0 | Mutant | Non-codel | high | 1 |
| CGGA_1610 | -0.293626731 | -0.233765228 | -0.059861503 | Recurrent | rAOA | WHO III | Male | 39 | 1058 | 1 | 1 | 1 | NA | Codel | high | 1 |
| CGGA_1611 | -0.113056726 | -0.397329583 | 0.284272857 | Recurrent | rGBM | WHO IV | Female | 30 | 1158 | 1 | 1 | 1 | Mutant | Non-codel | high | 0 |
| CGGA_1612 | 0.344164862 | -0.153335038 | 0.4974999 | Primary | GBM | WHO IV | Male | 68 | 718 | 1 | 1 | 1 | Wildtype | Non-codel | high | 0 |
| CGGA_1613 | 0.221385842 | -0.359855369 | 0.581241211 | Primary | GBM | WHO IV | Male | 53 | 250 | 0 | 1 | 1 | Wildtype | Non-codel | high | 0 |
| CGGA_1614 | -0.6342431 | -0.252542837 | -0.381700263 | Recurrent | rAA | WHO III | Male | 51 | 1079 | 1 | 1 | 1 | Mutant | Non-codel | low | 1 |
| CGGA_1615 | -0.285752888 | -0.1289341 | -0.156818788 | Recurrent | rGBM | WHO IV | Male | 48 | 586 | 1 | 1 | 1 | Wildtype | Codel | low | 0 |
| CGGA_1617 | -0.325426139 | -0.076499042 | -0.248927097 | Primary | O | WHO II | Female | 35 | 1611 | 0 | 1 | 0 | NA | Codel | low | 1 |
| CGGA_1618 | -0.24578722 | -0.247650611 | 0.001863391 | Primary | AOA | WHO III | Male | 31 | 1615 | 0 | 1 | 1 | Mutant | Non-codel | high | 1 |
| CGGA_1619 | -0.247906234 | -0.284537786 | 0.036631552 | Primary | AA | WHO III | Male | 44 | 766 | 1 | 1 | 1 | Mutant | Non-codel | high | 1 |
| CGGA_1620 | -0.347331133 | 0.236892791 | -0.584223924 | Primary | AOA | WHO III | Male | 51 | 1427 | 0 | 1 | 1 | Mutant | Codel | low | 1 |
| CGGA_1621 | -0.427808924 | -0.364978923 | -0.062830001 | Primary | OA | WHO II | Female | 48 | 1607 | 0 | 0 | 0 | Mutant | Non-codel | high | 1 |
| CGGA_1623 | -0.466094126 | 0.170405806 | -0.636499932 | Primary | AOA | WHO III | Female | 16 | 1603 | 0 | 1 | 0 | Wildtype | Non-codel | low | 1 |
| CGGA_1624 | 0.255242078 | -0.224266345 | 0.479508423 | Recurrent | rGBM | WHO IV | Female | 27 | 87 | 1 | 1 | 1 | Mutant | Non-codel | high | 0 |
| CGGA_1626 | -0.057432842 | -0.374023857 | 0.316591015 | Primary | GBM | WHO IV | Male | 66 | 696 | 1 | 1 | 1 | Wildtype | Non-codel | high | 0 |
| CGGA_1627 | -0.264446144 | 0.250062028 | -0.514508172 | Primary | AOA | WHO III | Male | 37 | 1597 | 0 | 1 | 1 | Mutant | Non-codel | low | 1 |
| CGGA_1630 | -0.330290397 | -0.108880398 | -0.221409999 | Primary | O | WHO II | Male | 26 | 1407 | 0 | 0 | 0 | NA | Codel | low | 1 |
| CGGA_1631 | 0.141405242 | -0.260073899 | 0.401479141 | Recurrent | rGBM | WHO IV | Female | 58 | 221 | 1 | 1 | 0 | Wildtype | Non-codel | high | 0 |
| CGGA_1634 | 0.332446235 | -0.164202017 | 0.496648252 | Primary | GBM | WHO IV | Female | 26 | 366 | 1 | 0 | 0 | Wildtype | Non-codel | high | 0 |
| CGGA_1635 | -0.077071799 | 0.256047109 | -0.333118908 | Primary | GBM | WHO IV | Female | 43 | 332 | 1 | 1 | 1 | Wildtype | NA | low | 0 |
| CGGA_1640 | -0.527309377 | -0.160758454 | -0.366550923 | Primary | AOA | WHO III | Male | 55 | 1563 | 0 | 1 | 1 | NA | Non-codel | low | 1 |
| CGGA_1641 | 0.751274625 | 0.118549744 | 0.632724881 | Recurrent | rGBM | WHO IV | Female | 57 | 291 | 1 | 1 | 1 | Wildtype | Non-codel | high | 0 |
| CGGA_1642 | -0.540061617 | -0.327422303 | -0.212639314 | Primary | A | WHO II | Male | 48 | 1562 | 0 | 1 | 0 | Mutant | Non-codel | low | 1 |
| CGGA_1643 | 0.481521801 | -0.002913056 | 0.484434857 | Primary | GBM | WHO IV | Female | 50 | 170 | 0 | NA | NA | Wildtype | Non-codel | high | 0 |
| CGGA_1644 | 0.084333042 | -0.406626405 | 0.490959447 | Primary | GBM | WHO IV | Male | 48 | 173 | 1 | 1 | 1 | Wildtype | Non-codel | high | 0 |
| CGGA_1645 | -0.231628321 | -0.015625497 | -0.216002824 | Primary | AA | WHO III | Male | 59 | 1534 | 0 | 1 | 1 | Mutant | Non-codel | low | 1 |
| CGGA_1647 | -0.353042687 | -0.252083842 | -0.100958845 | Primary | O | WHO II | Male | 40 | 1525 | 0 | 1 | 1 | NA | Codel | high | 1 |
| CGGA_1648 | -0.438406194 | -0.232128896 | -0.206277298 | Primary | AOA | WHO III | Female | 45 | 1521 | 0 | 1 | 1 | NA | Codel | low | 1 |
| CGGA_1649 | -0.318323611 | -0.204466224 | -0.113857387 | Primary | OA | WHO II | Male | 36 | 1047 | 0 | 1 | 1 | Mutant | Codel | low | 1 |
| CGGA_165 | -0.277471827 | -0.281812926 | 0.004341099 | Primary | A | WHO II | Male | 34 | 183 | 0 | 1 | 0 | NA | Non-codel | high | 1 |
| CGGA_1650 | -0.300903311 | 0.195595328 | -0.496498639 | Primary | GBM | WHO IV | Male | 36 | 1283 | 1 | 1 | 1 | Mutant | Non-codel | low | 0 |
| CGGA_1651 | -0.145618218 | 0.279873616 | -0.425491834 | Recurrent | rA | WHO II | Female | 44 | 842 | 1 | 1 | 1 | Mutant | Non-codel | low | 1 |
| CGGA_1653 | -0.460240527 | -0.242140214 | -0.218100313 | Primary | OA | WHO II | Female | 47 | 1513 | 0 | 1 | 0 | NA | Non-codel | low | 1 |
| CGGA_1654 | 0.188663418 | -0.318478418 | 0.507141836 | Primary | GBM | WHO IV | Male | 61 | NA | NA | NA | NA | Wildtype | Non-codel | high | 0 |
| CGGA_1655 | -0.463139186 | -0.287777812 | -0.175361374 | Primary | OA | WHO II | Male | 48 | 1209 | 0 | 1 | 0 | Mutant | Non-codel | low | 1 |
| CGGA_1656 | -0.535077505 | -0.428783154 | -0.106294351 | Recurrent | rGBM | WHO IV | Male | 56 | 149 | 1 | 1 | 1 | Wildtype | Non-codel | low | 0 |
| CGGA_1657 | -0.136727279 | 0.244556257 | -0.381283536 | Primary | AOA | WHO III | Female | 20 | 1511 | 0 | 0 | 0 | NA | Non-codel | low | 1 |
| CGGA_1658 | -0.004034907 | -0.166979917 | 0.16294501 | Recurrent | rGBM | WHO IV | Female | 64 | 1507 | 0 | 1 | 1 | Wildtype | Non-codel | high | 0 |
| CGGA_1659 | 0.100647529 | -0.179321608 | 0.279969137 | Recurrent | rGBM | WHO IV | Male | 24 | 387 | 1 | 1 | 1 | Wildtype | Non-codel | high | 0 |
| CGGA_1660 | -0.216487925 | -0.213384255 | -0.00310367 | Primary | OA | WHO II | Male | 41 | 433 | 0 | 1 | 1 | Wildtype | Codel | high | 1 |
| CGGA_1661 | -0.254384483 | -0.285590614 | 0.031206131 | Primary | AA | WHO III | Female | 42 | 1505 | 0 | 1 | 1 | NA | Non-codel | high | 1 |
| CGGA_1662 | -0.308760147 | 0.053262571 | -0.362022718 | Primary | OA | WHO II | Female | 55 | 1493 | 0 | 1 | 0 | Mutant | Codel | low | 1 |
| CGGA_1663 | -0.110837779 | -0.244801119 | 0.13396334 | Recurrent | rGBM | WHO IV | Male | 61 | 290 | 1 | 1 | 1 | Wildtype | Non-codel | high | 0 |
| CGGA_1664 | -0.139416455 | 0.264288627 | -0.403705082 | Primary | O | WHO II | Female | 40 | 1491 | 0 | 1 | 0 | Mutant | Non-codel | low | 1 |
| CGGA_1666 | -0.248463693 | -0.015521668 | -0.232942025 | Primary | GBM | WHO IV | Male | 60 | 249 | 1 | 1 | 1 | Wildtype | NA | low | 0 |
| CGGA_1667 | -0.154787284 | 0.352163464 | -0.506950748 | Primary | AO | WHO III | Female | 40 | 1476 | 0 | 1 | 1 | Mutant | Codel | low | 1 |
| CGGA_1669 | 0.376311674 | -0.008101699 | 0.384413373 | Primary | AOA | WHO III | Female | 44 | 139 | 1 | 0 | 0 | Wildtype | Non-codel | high | 1 |
| CGGA_1670 | 0.550361974 | -0.03192664 | 0.582288614 | Recurrent | rAA | WHO III | Female | 30 | 1141 | 0 | 1 | 1 | Wildtype | Non-codel | high | 1 |
| CGGA_1671 | 0.318539617 | -0.388385177 | 0.706924794 | Primary | AOA | WHO III | Female | 26 | 1464 | 0 | 1 | 1 | Mutant | Non-codel | high | 1 |
| CGGA_1672 | -0.281970732 | -0.08041817 | -0.201552562 | Primary | AOA | WHO III | Female | 49 | NA | NA | NA | NA | NA | Non-codel | low | 1 |
| CGGA_1673 | -0.370277642 | 0.222374822 | -0.592652464 | Primary | OA | WHO II | Female | 23 | 1457 | 0 | 0 | 0 | Mutant | Non-codel | low | 1 |
| CGGA_1675 | -0.258328071 | -0.365040862 | 0.106712791 | Primary | AA | WHO III | Male | 38 | 980 | 0 | 1 | 1 | Mutant | Non-codel | high | 1 |
| CGGA_1677 | -0.632393879 | -0.246763286 | -0.385630593 | Primary | A | WHO II | Female | 39 | NA | NA | NA | NA | NA | Non-codel | low | 1 |
| CGGA_1678 | -0.309088916 | -0.028105749 | -0.280983167 | Primary | GBM | WHO IV | Male | 51 | 657 | 1 | 1 | 1 | Wildtype | NA | low | 0 |
| CGGA_1679 | -0.276480203 | 0.0044329 | -0.280913103 | Primary | AOA | WHO III | Female | 52 | 1442 | 0 | 1 | 1 | Mutant | Codel | low | 1 |
| CGGA_1680 | -0.648715659 | -0.267551806 | -0.381163853 | Primary | AA | WHO III | Male | 45 | 596 | 1 | 1 | 1 | Mutant | Non-codel | low | 1 |
| CGGA_1681 | -0.24264491 | -0.267314634 | 0.024669724 | Primary | GBM | WHO IV | Female | 58 | 346 | 1 | 1 | 1 | Wildtype | Codel | high | 0 |
| CGGA_1682 | 0.309311133 | 0.164490522 | 0.144820611 | Recurrent | rGBM | WHO IV | Male | 67 | 345 | 1 | 1 | 1 | Wildtype | Non-codel | high | 0 |
| CGGA_1684 | 0.451549518 | -0.038052862 | 0.48960238 | Primary | GBM | WHO IV | Male | 51 | NA | NA | NA | NA | Wildtype | Non-codel | high | 0 |
| CGGA_1685 | 0.652511204 | 0.205596224 | 0.44691498 | Recurrent | rAA | WHO III | Male | 33 | 225 | 1 | 1 | 1 | Mutant | Non-codel | high | 1 |
| CGGA_1686 | 0.207222335 | -0.197561861 | 0.404784196 | Primary | AA | WHO III | Male | 27 | 352 | 0 | 0 | 1 | Wildtype | Non-codel | high | 1 |
| CGGA_1687 | 0.295909266 | -0.269401432 | 0.565310698 | Primary | GBM | WHO IV | Male | 14 | 1414 | 0 | 1 | 1 | Wildtype | Non-codel | high | 0 |
| CGGA_1688 | -0.329370109 | 0.102470646 | -0.431840755 | Recurrent | rAA | WHO III | Female | 51 | 51 | 1 | 1 | 1 | Wildtype | Non-codel | low | 1 |
| CGGA_1689 | -0.820141403 | -0.323157896 | -0.496983507 | Primary | A | WHO II | Male | 45 | 1414 | 0 | 1 | 0 | Mutant | Non-codel | low | 1 |
| CGGA_1690 | 0.716603092 | 0.004488372 | 0.71211472 | Primary | GBM | WHO IV | Male | 60 | 592 | 1 | 1 | 1 | Wildtype | Non-codel | high | 0 |
| CGGA_1693 | -0.071479274 | -0.022638193 | -0.048841081 | Primary | AO | WHO III | Male | 61 | 517 | 1 | 1 | 1 | NA | Non-codel | high | 1 |
| CGGA_1694 | -0.020831691 | -0.438374971 | 0.41754328 | Primary | GBM | WHO IV | Male | 55 | 624 | 1 | 1 | 1 | Wildtype | Non-codel | high | 0 |
| CGGA_1695 | -0.48639556 | -0.360040508 | -0.126355052 | Recurrent | rAA | WHO III | Male | 45 | 927 | 0 | 1 | 1 | Mutant | Non-codel | low | 1 |
| CGGA_1696 | -0.314907345 | 0.035955732 | -0.350863077 | Primary | AA | WHO III | Male | 72 | 1205 | 0 | 1 | 1 | Wildtype | NA | low | 1 |
| CGGA_1697 | 0.448212525 | 0.146370053 | 0.301842472 | Recurrent | rGBM | WHO IV | Female | 47 | 360 | 0 | NA | NA | Wildtype | Non-codel | high | 0 |
| CGGA_1698 | 0.268042473 | -0.329660835 | 0.597703308 | Primary | GBM | WHO IV | Female | 55 | 388 | 1 | 0 | 0 | Wildtype | Non-codel | high | 0 |
| CGGA_1699 | -0.412021435 | 0.013491963 | -0.425513398 | Primary | GBM | WHO IV | Female | 41 | 1384 | 0 | 1 | 1 | Mutant | NA | low | 0 |
| CGGA_1700 | -0.016140275 | -0.0588166 | 0.042676325 | Primary | AOA | WHO III | Male | 49 | 1372 | 0 | 1 | 1 | Mutant | Codel | high | 1 |
| CGGA_1701 | -0.556544312 | -0.310077426 | -0.246466886 | Primary | AOA | WHO III | Male | 44 | 1072 | 0 | 1 | 1 | Mutant | Codel | low | 1 |
| CGGA_1702 | 0.43706887 | -0.101256383 | 0.538325253 | Recurrent | rGBM | WHO IV | Female | 29 | 168 | 1 | 1 | 1 | Wildtype | Non-codel | high | 0 |
| CGGA_1703 | -0.360975556 | -0.160374038 | -0.200601518 | Primary | OA | WHO II | Female | 30 | 1366 | 0 | 1 | 1 | Mutant | Non-codel | low | 1 |
| CGGA_1704 | -0.694533607 | -0.349852357 | -0.34468125 | Primary | A | WHO II | Female | 32 | 1360 | 0 | 1 | 0 | Wildtype | Non-codel | low | 1 |
| CGGA_1706 | 0.123072417 | -0.367914442 | 0.490986859 | Primary | GBM | WHO IV | Male | 60 | 1364 | 0 | 1 | 1 | Wildtype | Non-codel | high | 0 |
| CGGA_1708 | -0.200202498 | -0.478849604 | 0.278647106 | Primary | GBM | WHO IV | Female | 54 | 1122 | 1 | 1 | 1 | Wildtype | Non-codel | high | 0 |
| CGGA_1709 | 0.190266134 | -0.451578861 | 0.641844995 | Primary | GBM | WHO IV | Male | 47 | 415 | 1 | 1 | 1 | Wildtype | Non-codel | high | 0 |
| CGGA_1713 | 0.767519388 | 0.100180419 | 0.667338969 | Primary | GBM | WHO IV | Male | 62 | 332 | 1 | 1 | 1 | Wildtype | Non-codel | high | 0 |
| CGGA_1714 | 0.038942585 | -0.150782296 | 0.189724881 | Primary | AOA | WHO III | Female | 37 | 1330 | 0 | 1 | 1 | NA | Codel | high | 1 |
| CGGA_1715 | -0.466950841 | 0.107660979 | -0.57461182 | Primary | O | WHO II | Female | 12 | 872 | 0 | 0 | 0 | Wildtype | Non-codel | low | 1 |
| CGGA_1716 | -0.113616692 | 0.070832182 | -0.184448874 | Recurrent | rAA | WHO III | Male | 38 | 1332 | 0 | 1 | 1 | Mutant | Non-codel | low | 1 |
| CGGA_1718 | -0.594996385 | -0.320098877 | -0.274897508 | Primary | AA | WHO III | Female | 37 | 835 | 0 | 1 | 1 | Mutant | Non-codel | low | 1 |
| CGGA_1720 | -0.200904645 | 0.07559223 | -0.276496875 | Primary | OA | WHO II | Male | 35 | 1290 | 0 | 1 | 1 | Mutant | Non-codel | low | 1 |
| CGGA_1721 | -0.522164247 | -0.206099701 | -0.316064546 | Primary | AO | WHO III | Female | 46 | 1290 | 0 | 1 | 1 | NA | Codel | low | 1 |
| CGGA_1722 | -0.17314409 | 0.005508464 | -0.178652554 | Primary | GBM | WHO IV | Female | 60 | 349 | 1 | 1 | 1 | Wildtype | NA | low | 0 |
| CGGA_1723 | -0.396240961 | -0.088656044 | -0.307584917 | Primary | AOA | WHO III | Female | 39 | 1282 | 0 | 1 | 1 | Mutant | Codel | low | 1 |
| CGGA_1725 | 0.11713914 | -0.019509592 | 0.136648732 | Primary | AOA | WHO III | Female | 33 | 1282 | 0 | 1 | 1 | Mutant | Codel | high | 1 |
| CGGA_1727 | -0.656188399 | -0.303692025 | -0.352496374 | Primary | GBM | WHO IV | Male | 48 | 1276 | 0 | 1 | 1 | Mutant | Non-codel | low | 0 |
| CGGA_1728 | 0.27439103 | -0.130670296 | 0.405061326 | Primary | GBM | WHO IV | Male | 45 | 917 | 1 | 1 | 1 | Mutant | Non-codel | high | 0 |
| CGGA_1729 | 0.331934145 | 0.035270601 | 0.296663544 | Recurrent | rGBM | WHO IV | Male | 52 | 244 | 1 | 1 | 1 | Wildtype | Non-codel | high | 0 |
| CGGA_1731 | -0.002743205 | -0.205803105 | 0.2030599 | Primary | AOA | WHO III | Female | 45 | 972 | 0 | 1 | 1 | Mutant | Non-codel | high | 1 |
| CGGA_1735 | 0.185834677 | 0.32843807 | -0.142603393 | Primary | GBM | WHO IV | Male | 54 | 813 | 1 | 1 | 1 | Wildtype | Non-codel | low | 0 |
| CGGA_1736 | 0.257545781 | -0.081900122 | 0.339445903 | Primary | GBM | WHO IV | Female | 57 | 938 | 1 | 1 | 1 | Wildtype | Non-codel | high | 0 |
| CGGA_1737 | -0.114735489 | -0.149136515 | 0.034401026 | Primary | AO | WHO III | Male | 33 | 1248 | 0 | 1 | 1 | Mutant | Codel | high | 1 |
| CGGA_1738 | 0.463345451 | -0.023915752 | 0.487261203 | Primary | AA | WHO III | Male | 49 | 469 | 1 | 1 | 1 | Wildtype | Non-codel | high | 1 |
| CGGA_1739 | 0.501800295 | 0.159844114 | 0.341956181 | Recurrent | rAOA | WHO III | Male | 45 | 515 | 1 | 1 | 1 | Mutant | Non-codel | high | 1 |
| CGGA_1740 | 0.968306523 | 0.375663132 | 0.592643391 | Primary | GBM | WHO IV | Female | 50 | 363 | 1 | 0 | 0 | Wildtype | Non-codel | high | 0 |
| CGGA_1743 | -0.167122965 | 0.373989341 | -0.541112306 | Primary | AOA | WHO III | Female | 47 | 1232 | 0 | 0 | 0 | Mutant | Non-codel | low | 1 |
| CGGA_1744 | -0.165015006 | -0.366394484 | 0.201379478 | Primary | GBM | WHO IV | Male | 51 | 1232 | 0 | 1 | 1 | NA | Non-codel | high | 0 |
| CGGA_1745 | -0.176756876 | 0.261317065 | -0.438073941 | Primary | AOA | WHO III | Female | 45 | 1225 | 0 | 1 | 1 | Mutant | NA | low | 1 |
| CGGA_1747 | -0.554065037 | -0.337926679 | -0.216138358 | Primary | AOA | WHO III | Female | 27 | 293 | 1 | 1 | 1 | NA | Non-codel | low | 1 |
| CGGA_1749 | 0.699066352 | 0.173904552 | 0.5251618 | Primary | GBM | WHO IV | Female | 44 | 401 | 1 | 1 | 1 | Wildtype | Non-codel | high | 0 |
| CGGA_1750 | 0.010960779 | 0.265489119 | -0.25452834 | Primary | GBM | WHO IV | Female | 52 | 250 | 1 | 1 | 1 | Wildtype | Non-codel | low | 0 |
| CGGA_1758 | 0.409237499 | -0.078804309 | 0.488041808 | Primary | GBM | WHO IV | Female | 45 | 414 | 1 | 1 | 1 | Wildtype | Non-codel | high | 0 |
| CGGA_1760 | -0.340283381 | -0.280930044 | -0.059353337 | Recurrent | rA | WHO II | Male | 51 | 459 | 1 | 1 | 1 | Wildtype | Non-codel | high | 1 |
| CGGA_1764 | 0.490649623 | -0.120707617 | 0.61135724 | Primary | GBM | WHO IV | Male | 33 | 710 | 1 | 1 | 1 | Mutant | Non-codel | high | 0 |
| CGGA_1767 | -0.131693061 | -0.15494526 | 0.023252199 | Primary | GBM | WHO IV | Male | 63 | 309 | 0 | 0 | 0 | Wildtype | NA | high | 0 |
| CGGA_1769 | -0.025781754 | 0.493970319 | -0.519752073 | Primary | GBM | WHO IV | Female | 49 | 1151 | 0 | 1 | 1 | Wildtype | Non-codel | low | 0 |
| CGGA_1770 | 0.504846127 | -0.17980538 | 0.684651507 | Recurrent | rGBM | WHO IV | Female | 43 | 298 | 1 | 0 | 1 | Mutant | Non-codel | high | 0 |
| CGGA_1771 | 0.037652715 | 0.441258457 | -0.403605742 | Recurrent | rAOA | WHO III | Male | 38 | 1068 | 1 | 1 | 1 | Mutant | Non-codel | low | 1 |
| CGGA_1773 | 0.552770151 | -0.208986048 | 0.761756199 | Recurrent | rGBM | WHO IV | Female | 46 | 104 | 1 | 1 | 1 | Wildtype | Non-codel | high | 0 |
| CGGA_1776 | 0.122256677 | 0.42423629 | -0.301979613 | Recurrent | rGBM | WHO IV | Male | 23 | 259 | 1 | 1 | 1 | Wildtype | Non-codel | low | 0 |
| CGGA_1780 | -0.350670532 | -0.211599597 | -0.139070935 | Primary | GBM | WHO IV | Female | 57 | 1123 | 0 | 1 | 1 | Wildtype | NA | low | 0 |
| CGGA_1785 | 0.480630425 | -0.308379847 | 0.789010272 | Recurrent | rGBM | WHO IV | Male | 34 | 329 | 1 | 1 | 1 | Mutant | Non-codel | high | 0 |
| CGGA_1786 | -0.235193335 | -0.267434478 | 0.032241143 | Recurrent | rAO | WHO III | Female | 54 | 1128 | 0 | 1 | 1 | Mutant | Codel | high | 1 |
| CGGA_1791 | -0.156291533 | 0.243473183 | -0.399764716 | Primary | AA | WHO III | Male | 55 | 855 | 1 | 1 | 1 | Wildtype | NA | low | 1 |
| CGGA_1807 | 0.832323052 | 0.123478872 | 0.70884418 | Primary | GBM | WHO IV | Female | 65 | 247 | 1 | NA | NA | Wildtype | Non-codel | high | 0 |
| CGGA_1809 | 0.512645085 | 0.114407011 | 0.398238074 | Primary | AO | WHO III | Male | 27 | 343 | 1 | 1 | 1 | Wildtype | Non-codel | high | 1 |
| CGGA_1811 | -0.422341689 | -0.251520121 | -0.170821568 | Recurrent | rGBM | WHO IV | Male | 64 | 304 | 1 | 1 | 1 | Wildtype | Non-codel | low | 0 |
| CGGA_1812 | -0.051282532 | -0.211776862 | 0.16049433 | Primary | GBM | WHO IV | Male | 65 | NA | NA | NA | NA | Wildtype | Non-codel | high | 0 |
| CGGA_1814 | 0.32876398 | -0.046206656 | 0.374970636 | Recurrent | rGBM | WHO IV | Female | 46 | 221 | 1 | 1 | 1 | Wildtype | Non-codel | high | 0 |
| CGGA_1815 | 0.409348242 | 0.096605838 | 0.312742404 | Recurrent | rGBM | WHO IV | Female | 45 | 185 | 1 | 1 | 1 | Wildtype | Non-codel | high | 0 |
| CGGA_1817 | -0.756155933 | -0.3949223 | -0.361233633 | Primary | GBM | WHO IV | Female | 72 | 399 | 1 | 1 | 1 | Wildtype | Non-codel | low | 0 |
| CGGA_1819 | 0.038205269 | -0.215273119 | 0.253478388 | Primary | GBM | WHO IV | Male | 55 | 1008 | 0 | 1 | 1 | Wildtype | Non-codel | high | 0 |
| CGGA_1820 | 0.402462936 | -0.230241683 | 0.632704619 | Recurrent | rGBM | WHO IV | Female | 52 | 77 | 1 | 1 | 1 | Wildtype | Non-codel | high | 0 |
| CGGA_1826 | -0.101555773 | -0.201726696 | 0.100170923 | Primary | GBM | WHO IV | Female | 70 | 44 | 1 | 0 | 0 | Wildtype | Non-codel | high | 0 |
| CGGA_1829 | -0.279664497 | 0.245573853 | -0.52523835 | Primary | OA | WHO II | Male | 23 | 801 | 0 | 0 | 0 | Wildtype | Non-codel | low | 1 |
| CGGA_1833 | 0.591420935 | -0.053648745 | 0.64506968 | Primary | GBM | WHO IV | Female | 60 | 494 | 1 | 1 | 1 | Wildtype | Non-codel | high | 0 |
| CGGA_1840 | 0.823376346 | 0.445519412 | 0.377856934 | Primary | GBM | WHO IV | Female | 58 | 964 | 0 | 1 | 1 | Wildtype | Non-codel | high | 0 |
| CGGA_1850 | -0.249286783 | -0.167770008 | -0.081516775 | Primary | OA | WHO II | Male | 29 | 750 | 0 | 0 | 1 | Mutant | Non-codel | high | 1 |
| CGGA_1854 | -0.100747034 | -0.283802903 | 0.183055869 | Primary | AA | WHO III | Male | 36 | 934 | 0 | 1 | 1 | Mutant | Non-codel | high | 1 |
| CGGA_1857 | 0.283104591 | -0.235900855 | 0.519005446 | Recurrent | rGBM | WHO IV | Male | 46 | 157 | 1 | 1 | 1 | Wildtype | Non-codel | high | 0 |
| CGGA_1862 | 0.378256378 | -0.228372111 | 0.606628489 | Primary | AA | WHO III | Male | 40 | 110 | 1 | 1 | 1 | Wildtype | Non-codel | high | 1 |
| CGGA_1863 | 0.288507805 | -0.065354393 | 0.353862198 | Recurrent | rAOA | WHO III | Female | 50 | 902 | 0 | 1 | 1 | Mutant | Codel | high | 1 |
| CGGA_1865 | -0.570476489 | -0.264317399 | -0.30615909 | Recurrent | rGBM | WHO IV | Female | 45 | 89 | 1 | 0 | 0 | Wildtype | Non-codel | low | 0 |
| CGGA_1866 | 0.240728031 | -0.374999109 | 0.61572714 | Primary | GBM | WHO IV | Male | 68 | 127 | 1 | 1 | 1 | Wildtype | Non-codel | high | 0 |
| CGGA_1870 | 0.259109889 | -0.121731327 | 0.380841216 | Primary | GBM | WHO IV | Male | 62 | 864 | 0 | 1 | 1 | Mutant | Codel | high | 0 |
| CGGA_1875 | -0.145480435 | -0.236625779 | 0.091145344 | Primary | OA | WHO II | Female | 22 | 866 | 0 | 1 | 1 | Wildtype | Non-codel | high | 1 |
| CGGA_1877 | -0.184565837 | -0.032592918 | -0.151972919 | Recurrent | rAOA | WHO III | Male | 35 | NA | NA | NA | NA | Mutant | Codel | low | 1 |
| CGGA_1880 | -0.33439442 | -0.480461124 | 0.146066704 | Recurrent | rAA | WHO III | Female | 37 | 855 | 0 | 0 | 0 | Mutant | Non-codel | high | 1 |
| CGGA_1882 | -0.25854483 | -0.342755325 | 0.084210495 | Primary | AOA | WHO III | Male | 33 | 842 | 0 | 0 | 0 | Mutant | Codel | high | 1 |
| CGGA_1886 | 0.279191357 | -0.336020716 | 0.615212073 | Recurrent | rGBM | WHO IV | Male | 31 | 338 | 1 | 1 | 1 | Wildtype | Non-codel | high | 0 |
| CGGA_1899 | -0.52726167 | -0.216067979 | -0.311193691 | Recurrent | rGBM | WHO IV | Male | 45 | 176 | 1 | 1 | 1 | Wildtype | Non-codel | low | 0 |
| CGGA_1901 | -0.030621471 | -0.359028552 | 0.328407081 | Primary | GBM | WHO IV | Male | 60 | 540 | 1 | 1 | 1 | Wildtype | Non-codel | high | 0 |
| CGGA_1902 | -0.123858126 | -0.180084376 | 0.05622625 | Primary | OA | WHO II | Male | 33 | 790 | 0 | 0 | 0 | Mutant | Non-codel | high | 1 |
| CGGA_1903 | -0.684591309 | -0.26588631 | -0.418704999 | Primary | AOA | WHO III | Female | 24 | 785 | 0 | 1 | 1 | NA | Codel | low | 1 |
| CGGA_1906 | -0.244790246 | -0.063331714 | -0.181458532 | Recurrent | rAA | WHO III | Male | 57 | 779 | 0 | 1 | 1 | Mutant | Non-codel | low | 1 |
| CGGA_1908 | 0.600707212 | -0.055952517 | 0.656659729 | Recurrent | rGBM | WHO IV | Female | 42 | 298 | 1 | 1 | 1 | Wildtype | Non-codel | high | 0 |
| CGGA_1911 | 0.300848504 | -0.359150827 | 0.659999331 | Recurrent | rGBM | WHO IV | Male | 15 | 762 | 0 | 1 | 1 | Wildtype | Non-codel | high | 0 |
| CGGA_1912 | -0.232888074 | -0.218705165 | -0.014182909 | Recurrent | rGBM | WHO IV | Male | 48 | NA | NA | 1 | 1 | NA | Non-codel | high | 0 |
| CGGA_1916 | 0.42706976 | -0.001116069 | 0.428185829 | Primary | GBM | WHO IV | Female | 33 | 0 | 0 | 0 | 0 | Mutant | Non-codel | high | 0 |
| CGGA_1939 | -0.597283906 | -0.285462566 | -0.31182134 | Recurrent | rA | WHO II | Male | 31 | NA | NA | 1 | 1 | NA | Non-codel | low | 1 |
| CGGA_194 | -0.216115989 | 0.228264322 | -0.444380311 | Recurrent | rAO | WHO III | Female | 43 | 2304 | 1 | 1 | 0 | Mutant | Codel | low | 1 |
| CGGA_1946 | 0.588414856 | -0.075158345 | 0.663573201 | Recurrent | rGBM | WHO IV | Female | 51 | 142 | 1 | 1 | 1 | Wildtype | Non-codel | high | 0 |
| CGGA_1953 | 0.13256374 | -0.413448825 | 0.546012565 | Recurrent | rGBM | WHO IV | Male | 47 | 73 | 1 | 1 | 1 | Wildtype | Non-codel | high | 0 |
| CGGA_1955 | -0.216862855 | -0.057416204 | -0.159446651 | Recurrent | rGBM | WHO IV | Male | 30 | NA | NA | 1 | 1 | NA | Non-codel | low | 0 |
| CGGA_1972 | 0.289792021 | -0.265423733 | 0.555215754 | Recurrent | rGBM | WHO IV | Male | 47 | 606 | 0 | NA | NA | Wildtype | Non-codel | high | 0 |
| CGGA_1976 | 0.133847427 | -0.414470329 | 0.548317756 | Recurrent | rGBM | WHO IV | Female | 53 | NA | NA | 1 | 1 | Wildtype | Non-codel | high | 0 |
| CGGA_1985 | 0.066015888 | -0.484102178 | 0.550118066 | Recurrent | rGBM | WHO IV | Male | 41 | 380 | 1 | NA | NA | Mutant | Non-codel | high | 0 |
| CGGA_1994 | -0.036892606 | 0.062949762 | -0.099842368 | Recurrent | rAO | WHO III | Female | 32 | 552 | NA | NA | NA | Mutant | Non-codel | high | 1 |
| CGGA_2002 | -0.035684167 | 0.18853379 | -0.224217957 | Recurrent | rA | WHO II | Male | 40 | 524 | 0 | 1 | 1 | Mutant | Non-codel | low | 1 |
| CGGA_2003 | 0.135806604 | 0.346693994 | -0.21088739 | Recurrent | rGBM | WHO IV | Male | 40 | 517 | 0 | 1 | 1 | Mutant | Non-codel | low | 0 |
| CGGA_2006 | 0.815967376 | 0.307989715 | 0.507977661 | Recurrent | rAA | WHO III | Male | 43 | 516 | 0 | 1 | 1 | Mutant | Non-codel | high | 1 |
| CGGA_2008 | 0.465242752 | 0.147583941 | 0.317658811 | Recurrent | rGBM | WHO IV | Male | 56 | 44 | 1 | NA | NA | Wildtype | Non-codel | high | 0 |
| CGGA_2013 | 0.203444007 | -0.153273781 | 0.356717788 | Recurrent | rA | WHO II | Male | 41 | 139 | 1 | 1 | NA | Mutant | Codel | high | 1 |
| CGGA_2024 | 0.050350941 | 0.382857149 | -0.332506208 | Recurrent | rGBM | WHO IV | Female | 19 | 328 | 1 | 1 | NA | Wildtype | Non-codel | low | 0 |
| CGGA_2038 | 0.892655229 | 0.293874654 | 0.598780575 | Recurrent | rGBM | WHO IV | Male | 44 | NA | NA | NA | NA | Mutant | Non-codel | high | 0 |
| CGGA_2039 | -0.020803178 | 0.153440288 | -0.174243466 | Recurrent | rGBM | WHO IV | Male | 28 | NA | NA | NA | NA | Wildtype | Non-codel | low | 0 |
| CGGA_2046 | 0.820820303 | 0.20405586 | 0.616764443 | Recurrent | rAA | WHO III | Male | 46 | NA | NA | 1 | 1 | Mutant | Non-codel | high | 1 |
| CGGA_2047 | 0.434261554 | 0.016443635 | 0.417817919 | Recurrent | rGBM | WHO IV | Female | 64 | NA | NA | NA | 1 | Wildtype | Non-codel | high | 0 |
| CGGA_2053 | 0.080820745 | 0.403064288 | -0.322243543 | Recurrent | rGBM | WHO IV | Male | 55 | NA | NA | 0 | 1 | Wildtype | Codel | low | 0 |
| CGGA_2056 | 0.455232769 | -0.268347651 | 0.72358042 | Recurrent | rGBM | WHO IV | Female | 41 | NA | NA | 1 | 1 | Mutant | Non-codel | high | 0 |
| CGGA_2062 | 0.744589577 | 0.06785026 | 0.676739317 | Recurrent | rGBM | WHO IV | Female | 63 | NA | NA | 1 | 1 | Mutant | Non-codel | high | 0 |
| CGGA_2075 | 0.450614586 | -0.081979258 | 0.532593844 | Recurrent | rGBM | WHO IV | Male | 63 | NA | NA | 1 | 1 | Wildtype | Non-codel | high | 0 |
| CGGA_2078 | 0.808259239 | 0.196178765 | 0.612080474 | Recurrent | rGBM | WHO IV | Female | 61 | NA | NA | 1 | 1 | Wildtype | Non-codel | high | 0 |
| CGGA_2079 | 0.552707948 | 0.031534277 | 0.521173671 | Recurrent | rAO | WHO III | Male | 45 | NA | NA | 1 | 1 | Mutant | Codel | high | 1 |
| CGGA_2081 | 0.376605122 | 0.395131967 | -0.018526845 | Recurrent | rAO | WHO III | Female | 39 | NA | NA | 1 | 0 | Mutant | Codel | high | 1 |
| CGGA_2082 | 0.832133546 | 0.132581193 | 0.699552353 | Recurrent | rGBM | WHO IV | Male | 41 | NA | NA | 1 | 1 | Wildtype | Non-codel | high | 0 |
| CGGA_2088 | 0.650135789 | 0.188312062 | 0.461823727 | Recurrent | rGBM | WHO IV | Female | 30 | NA | NA | 1 | 1 | Wildtype | Non-codel | high | 0 |
| CGGA_2106 | 0.698309482 | 0.291332984 | 0.406976498 | Recurrent | rGBM | WHO IV | Female | 39 | NA | NA | NA | NA | Mutant | Codel | high | 0 |
| CGGA_2115 | 0.856714351 | 0.23443046 | 0.622283891 | Recurrent | rGBM | WHO IV | Female | 31 | NA | NA | 1 | 1 | Wildtype | Non-codel | high | 0 |
| CGGA_2121 | -0.055868269 | 0.036456814 | -0.092325083 | Recurrent | rA | WHO II | Female | 41 | NA | NA | 1 | 1 | Mutant | Non-codel | high | 1 |
| CGGA_2129 | 0.735530839 | 0.131475368 | 0.604055471 | Recurrent | rAA | WHO III | Male | 48 | NA | NA | 0 | 0 | Mutant | Non-codel | high | 1 |
| CGGA_265 | -0.412955008 | -0.232879081 | -0.180075927 | Recurrent | rA | WHO II | Male | 43 | 3959 | 0 | 1 | 1 | Mutant | Non-codel | low | 1 |
| CGGA_279 | 0.361239477 | -0.287557705 | 0.648797182 | Recurrent | rAOA | WHO III | Female | 49 | 1314 | 1 | 1 | 1 | Mutant | Non-codel | high | 1 |
| CGGA_28 | -0.584064657 | -0.23423383 | -0.349830827 | Primary | A | WHO II | Male | 25 | 1103 | 1 | 1 | 0 | Mutant | Codel | low | 1 |
| CGGA_288 | 0.443477019 | 0.005815622 | 0.437661397 | Recurrent | rO | WHO II | Male | 49 | 1057 | 1 | 1 | 0 | Mutant | Codel | high | 1 |
| CGGA_290 | 0.038587743 | 0.43250017 | -0.393912427 | Primary | OA | WHO II | Female | 37 | 4075 | 0 | 1 | 0 | Mutant | Codel | low | 1 |
| CGGA_320 | 0.048468369 | 0.449471276 | -0.401002907 | Primary | OA | WHO II | Male | 31 | 3005 | 1 | 1 | 1 | Mutant | Codel | low | 1 |
| CGGA_336 | -0.142374491 | -0.216731931 | 0.07435744 | Recurrent | rAOA | WHO III | Female | 57 | 2549 | 1 | 1 | 1 | Mutant | Codel | high | 1 |
| CGGA_358 | -0.193282482 | -0.124573902 | -0.06870858 | Recurrent | rAOA | WHO III | Female | 30 | 122 | 1 | 0 | 0 | Mutant | Non-codel | high | 1 |
| CGGA_362 | -0.557269798 | -0.156148278 | -0.40112152 | Recurrent | rA | WHO II | Female | 39 | 1680 | 0 | 0 | NA | Mutant | Codel | low | 1 |
| CGGA_369 | -0.215288319 | 0.244683788 | -0.459972107 | Recurrent | rA | WHO II | Male | 33 | 3957 | 0 | 1 | 0 | Mutant | Non-codel | low | 1 |
| CGGA_406 | 0.772309261 | 0.11186101 | 0.660448251 | Recurrent | rAOA | WHO III | Female | 24 | 90 | 1 | 0 | 0 | Mutant | Non-codel | high | 1 |
| CGGA_42 | -0.410791707 | 0.050120289 | -0.460911996 | Primary | A | WHO II | Male | 38 | 2832 | 1 | 1 | 1 | Mutant | Codel | low | 1 |
| CGGA_420 | -0.129497222 | -0.009163404 | -0.120333818 | Recurrent | rAO | WHO III | Female | 39 | 3514 | 0 | 1 | 1 | Mutant | Codel | low | 1 |
| CGGA_457 | 0.444620309 | -0.211190332 | 0.655810641 | Recurrent | rAOA | WHO III | Male | 32 | 75 | 1 | 0 | 0 | Mutant | Non-codel | high | 1 |
| CGGA_474 | 0.049355962 | -0.372438327 | 0.421794289 | Primary | AO | WHO III | Male | NA | 2029 | 0 | 1 | 1 | Wildtype | Non-codel | high | 1 |
| CGGA_482 | 0.113090966 | 0.330155882 | -0.217064916 | Primary | OA | WHO II | Male | 45 | 2511 | 1 | 1 | 1 | Mutant | Codel | low | 1 |
| CGGA_487 | 0.208809962 | -0.024581065 | 0.233391027 | Recurrent | rGBM | WHO IV | Male | 46 | 156 | 1 | 1 | 1 | Wildtype | Non-codel | high | 0 |
| CGGA_492 | 0.659776101 | 0.147667781 | 0.51210832 | Recurrent | rAOA | WHO III | Male | 27 | 652 | 0 | 1 | 1 | Wildtype | Non-codel | high | 1 |
| CGGA_503 | -0.092803811 | 0.521331997 | -0.614135808 | Primary | OA | WHO II | Male | 51 | 3777 | 0 | 1 | 1 | Mutant | Non-codel | low | 1 |
| CGGA_507 | -0.10214053 | 0.441956296 | -0.544096826 | Recurrent | rAA | WHO III | Female | 53 | 328 | 1 | 0 | 1 | Mutant | Non-codel | low | 1 |
| CGGA_509 | 0.102347351 | -0.410301457 | 0.512648808 | Primary | GBM | WHO IV | Male | 38 | 623 | 1 | 1 | 1 | Wildtype | Non-codel | high | 0 |
| CGGA_521 | 0.093107398 | 0.046835103 | 0.046272295 | Primary | AA | WHO III | Male | 32 | 514 | 1 | 1 | 1 | Wildtype | Non-codel | high | 1 |
| CGGA_530 | 0.181414886 | -0.189615879 | 0.371030765 | Recurrent | rGBM | WHO IV | Female | 41 | 506 | 1 | 0 | 1 | Wildtype | Non-codel | high | 0 |
| CGGA_554 | 0.377187928 | -0.15407191 | 0.531259838 | Recurrent | rAOA | WHO III | Female | 46 | 134 | 1 | 1 | 0 | NA | Non-codel | high | 1 |
| CGGA_568 | 0.164573846 | -0.054378097 | 0.218951943 | Recurrent | rGBM | WHO IV | Male | 14 | 285 | 1 | 0 | 1 | Wildtype | Non-codel | high | 0 |
| CGGA_583 | -0.395994021 | 0.02754704 | -0.423541061 | Primary | A | WHO II | Female | 36 | 3107 | 1 | 1 | 0 | Mutant | NA | low | 1 |
| CGGA_619 | -0.313425107 | -0.208494178 | -0.104930929 | Recurrent | rA | WHO II | Male | 40 | 550 | 1 | 1 | 0 | Wildtype | Non-codel | low | 1 |
| CGGA_621 | 0.31559397 | -0.299119047 | 0.614713017 | Recurrent | rAOA | WHO III | Male | 40 | 1347 | 0 | 1 | 1 | Mutant | Non-codel | high | 1 |
| CGGA_625 | 0.127731237 | -0.124948856 | 0.252680093 | Recurrent | rAOA | WHO III | Female | 33 | 2098 | 1 | 0 | 0 | Mutant | Codel | high | 1 |
| CGGA_634 | -0.041772563 | 0.438746363 | -0.480518926 | Primary | AOA | WHO III | Female | 35 | 2633 | 1 | 1 | 1 | Mutant | Non-codel | low | 1 |
| CGGA_652 | 0.830949474 | 0.314946937 | 0.516002537 | Recurrent | rAA | WHO III | Male | 42 | 476 | 1 | 1 | 1 | Mutant | Non-codel | high | 1 |
| CGGA_663 | -0.108756119 | 0.489448692 | -0.598204811 | Primary | A | WHO II | Female | 29 | 3479 | 0 | 1 | 0 | Mutant | Non-codel | low | 1 |
| CGGA_703 | -0.157730094 | -0.246951576 | 0.089221482 | Recurrent | rAO | WHO III | Female | 31 | 1355 | 1 | 1 | 1 | Mutant | Non-codel | high | 1 |
| CGGA_705 | -0.509712256 | -0.217507592 | -0.292204664 | Primary | AA | WHO III | Male | 41 | 1507 | 1 | 1 | 0 | Mutant | Non-codel | low | 1 |
| CGGA_707 | -0.208841332 | 0.437011129 | -0.645852461 | Primary | OA | WHO II | Female | 39 | 3299 | 1 | 1 | 1 | Mutant | Codel | low | 1 |
| CGGA_720 | 0.197874984 | -0.321256192 | 0.519131176 | Recurrent | rA | WHO II | Female | 46 | 1258 | 1 | 1 | 0 | Mutant | Non-codel | high | 1 |
| CGGA_721 | -0.219428316 | 0.325354877 | -0.544783193 | Primary | OA | WHO II | Male | 45 | 2552 | 1 | 1 | 1 | Mutant | Non-codel | low | 1 |
| CGGA_724 | -0.249444356 | 0.209586539 | -0.459030895 | Recurrent | rOA | WHO II | Male | 32 | 3406 | 0 | 0 | 0 | Mutant | Non-codel | low | 1 |
| CGGA_730 | -0.275213286 | 0.105491589 | -0.380704875 | Recurrent | rOA | WHO II | Female | 51 | 2118 | 1 | 1 | 0 | Mutant | Codel | low | 1 |
| CGGA_763 | -0.079961275 | 0.548948062 | -0.628909337 | Recurrent | rAOA | WHO III | Male | 52 | 319 | 0 | 1 | 0 | Wildtype | Non-codel | low | 1 |
| CGGA_777 | 0.352389124 | 0.127375416 | 0.225013708 | Recurrent | rGBM | WHO IV | Male | 46 | 146 | 1 | 1 | 0 | Wildtype | Non-codel | high | 0 |
| CGGA_780 | -0.042221296 | 0.307134399 | -0.349355695 | Recurrent | rAO | WHO III | Female | 64 | 923 | 1 | 0 | 1 | Wildtype | Non-codel | low | 1 |
| CGGA_799 | -0.457573551 | -0.031318278 | -0.426255273 | Recurrent | rAOA | WHO III | Male | 53 | 1868 | 1 | 1 | 1 | Mutant | Codel | low | 1 |
| CGGA_809 | -0.622116839 | -0.207086349 | -0.41503049 | Primary | A | WHO II | Male | 26 | 1252 | 1 | 1 | 1 | Mutant | Non-codel | low | 1 |
| CGGA_810 | 0.4325321 | 0.199179232 | 0.233352868 | Recurrent | rAO | WHO III | Male | 20 | 551 | 1 | 1 | 1 | Wildtype | Non-codel | high | 1 |
| CGGA_825 | -0.096464987 | -0.397591213 | 0.301126226 | Recurrent | rAA | WHO III | Male | 36 | 836 | 1 | 1 | 1 | Mutant | Non-codel | high | 1 |
| CGGA_831 | 0.271742276 | -0.176127699 | 0.447869975 | Primary | GBM | WHO IV | Female | 55 | 546 | 1 | 1 | 1 | Wildtype | Non-codel | high | 0 |
| CGGA_846 | -0.22216363 | 0.192788518 | -0.414952148 | Recurrent | rA | WHO II | Female | 39 | 2991 | 0 | 0 | 1 | Wildtype | Non-codel | low | 1 |
| CGGA_852 | -0.182152107 | -0.1760684 | -0.006083707 | Primary | AA | WHO III | Male | 40 | 169 | 1 | 1 | 0 | Wildtype | Non-codel | high | 1 |
| CGGA_861 | -0.04809371 | 0.459371714 | -0.507465424 | Primary | AOA | WHO III | Male | 29 | 893 | 1 | 0 | 0 | Wildtype | Non-codel | low | 1 |
| CGGA_862 | -0.205417582 | 0.404343938 | -0.60976152 | Recurrent | rA | WHO II | Male | 30 | 1046 | 0 | 0 | NA | Mutant | Non-codel | low | 1 |
| CGGA_863 | -0.252471587 | 0.290707395 | -0.543178982 | Primary | A | WHO II | Female | 42 | 2073 | 1 | 1 | 0 | Wildtype | Non-codel | low | 1 |
| CGGA_867 | 0.31172677 | -0.296749239 | 0.608476009 | Recurrent | rAA | WHO III | Female | 40 | 367 | 1 | 0 | 0 | Mutant | Non-codel | high | 1 |
| CGGA_869 | 0.221064377 | -0.353577698 | 0.574642075 | Recurrent | rGBM | WHO IV | Female | 36 | 537 | 0 | NA | NA | Wildtype | Non-codel | high | 0 |
| CGGA_881 | 0.090779246 | -0.034967089 | 0.125746335 | Recurrent | rAOA | WHO III | Female | 23 | 252 | 1 | 1 | 1 | Wildtype | Non-codel | high | 1 |
| CGGA_882 | 0.066999749 | 0.589433672 | -0.522433923 | Recurrent | rAOA | WHO III | Male | 30 | 1008 | 0 | 1 | 0 | Mutant | Non-codel | low | 1 |
| CGGA_883 | 0.127950829 | 0.299420508 | -0.171469679 | Recurrent | rA | WHO II | Female | 39 | 1016 | 0 | 0 | NA | Mutant | Non-codel | low | 1 |
| CGGA_887 | -0.162148351 | 0.476812279 | -0.63896063 | Recurrent | rAO | WHO III | Male | 25 | 1011 | 0 | 0 | NA | Wildtype | Non-codel | low | 1 |
| CGGA_888 | 0.176780335 | 0.163969294 | 0.012811041 | Recurrent | rAO | WHO III | Male | 29 | 669 | 1 | 1 | 1 | Mutant | Codel | high | 1 |
| CGGA_889 | 0.100005267 | 0.475449968 | -0.375444701 | Primary | A | WHO II | Male | 23 | 3213 | 0 | 1 | 1 | Mutant | Non-codel | low | 1 |
| CGGA_890 | -0.560850917 | -0.00811385 | -0.552737067 | Primary | AA | WHO III | Male | 39 | 2982 | 1 | 1 | 1 | Mutant | Non-codel | low | 1 |
| CGGA_901 | -0.313386121 | 0.087896574 | -0.401282695 | Primary | AOA | WHO III | Male | 40 | 928 | 1 | NA | 1 | Mutant | Non-codel | low | 1 |
| CGGA_903 | 0.026720344 | 0.25934546 | -0.232625116 | Primary | AOA | WHO III | Female | 27 | 1455 | 1 | 1 | 0 | Mutant | Non-codel | low | 1 |
| CGGA_D19 | 0.538088489 | 0.251544467 | 0.286544022 | Recurrent | rO | WHO II | Female | 64 | 297 | 1 | 1 | 1 | Mutant | Codel | high | 1 |
| CGGA_D28 | -0.677280826 | -0.273189121 | -0.404091705 | Primary | AA | WHO III | Male | 25 | 860 | 1 | 1 | 0 | Mutant | Non-codel | low | 1 |
| CGGA_D49 | 0.231197099 | 0.209834943 | 0.021362156 | Recurrent | NA | NA | Female | 32 | NA | NA | NA | NA | Mutant | Codel | high | NA |
| CGGA_D50 | -0.062249927 | 0.309709496 | -0.371959423 | Recurrent | rOA | WHO II | Male | 30 | 782 | 1 | 0 | 0 | Mutant | Codel | low | 1 |
| CGGA_D53 | 0.335352802 | -0.226714265 | 0.562067067 | Recurrent | rGBM | WHO IV | Male | 30 | NA | NA | NA | NA | Wildtype | Non-codel | high | 0 |
| CGGA_J50 | -0.29146712 | 0.024006613 | -0.315473733 | Recurrent | rOA | WHO II | Male | 27 | 1693 | 1 | 1 | 1 | Mutant | NA | low | 1 |
| CGGA_J73 | 0.018630782 | 0.284955276 | -0.266324494 | Primary | AO | WHO III | Female | 34 | 1134 | 1 | 1 | 1 | Mutant | NA | low | 1 |
| CGGA_P100 | 0.318242566 | -0.365389868 | 0.683632434 | Primary | GBM | WHO IV | Male | 67 | 268 | 1 | NA | NA | Wildtype | Non-codel | high | 0 |
| CGGA_P102 | 0.501644373 | -0.135453042 | 0.637097415 | Primary | GBM | WHO IV | Male | 30 | 989 | 0 | 1 | 1 | Mutant | Non-codel | high | 0 |
| CGGA_P103 | -0.082130003 | 0.470824087 | -0.55295409 | Primary | OA | WHO II | Male | 34 | 983 | 0 | 1 | 0 | Mutant | Codel | low | 1 |
| CGGA_P104 | -0.260876094 | -0.123197116 | -0.137678978 | Recurrent | rAOA | WHO III | Female | 36 | 167 | 1 | 1 | 1 | Mutant | NA | low | 1 |
| CGGA_P106 | 0.725085022 | 0.248891722 | 0.4761933 | Recurrent | rGBM | WHO IV | Male | 24 | 71 | 1 | 1 | 1 | Wildtype | Non-codel | high | 0 |
| CGGA_P107 | -0.490188419 | -0.064556788 | -0.425631631 | Primary | OA | WHO II | Male | 26 | NA | NA | NA | NA | Mutant | Non-codel | low | 1 |
| CGGA_P108 | 0.018116756 | 0.401205962 | -0.383089206 | Primary | AOA | WHO III | Female | 30 | 976 | 0 | 1 | 1 | Wildtype | Non-codel | low | 1 |
| CGGA_P109 | 0.094820438 | -0.423617929 | 0.518438367 | Recurrent | rGBM | WHO IV | Male | 35 | 406 | 1 | 1 | 1 | Mutant | Non-codel | high | 0 |
| CGGA_P11 | -0.453642279 | -0.445179433 | -0.008462846 | Primary | A | WHO II | Female | 38 | 433 | 0 | NA | NA | Mutant | Non-codel | high | 1 |
| CGGA_P110 | 0.736815672 | 0.313736614 | 0.423079058 | Primary | AO | WHO III | Female | 56 | 800 | 1 | 1 | 1 | Wildtype | Non-codel | high | 1 |
| CGGA_P111 | -0.262038659 | 0.17000659 | -0.432045249 | Primary | OA | WHO II | Female | 44 | 971 | 0 | 1 | 0 | Mutant | Codel | low | 1 |
| CGGA_P112 | 0.022452571 | 0.515549289 | -0.493096718 | Primary | GBM | WHO IV | Male | 65 | 834 | 1 | 1 | 1 | Wildtype | Non-codel | low | 0 |
| CGGA_P113 | -0.071738324 | 0.547885984 | -0.619624308 | Primary | OA | WHO II | Male | 32 | 1032 | 0 | 1 | 0 | Wildtype | Non-codel | low | 1 |
| CGGA_P114 | -0.241966608 | 0.165858158 | -0.407824766 | Primary | OA | WHO II | Male | 32 | NA | NA | 1 | NA | Mutant | Codel | low | 1 |
| CGGA_P115 | -0.256311845 | 0.172663424 | -0.428975269 | Primary | OA | WHO II | Male | 25 | 959 | 0 | 1 | 0 | Wildtype | NA | low | 1 |
| CGGA_P116 | -0.13211319 | -0.254658026 | 0.122544836 | Primary | GBM | WHO IV | Male | 58 | 305 | 1 | 1 | NA | Wildtype | NA | high | 0 |
| CGGA_P121 | -0.284548361 | 0.036511196 | -0.321059557 | Primary | OA | WHO II | Male | 39 | 941 | 0 | 1 | 0 | Wildtype | Non-codel | low | 1 |
| CGGA_P122 | 0.166232534 | 0.263943161 | -0.097710627 | Recurrent | rOA | WHO II | Male | 43 | 971 | 0 | 1 | 1 | Mutant | Codel | high | 1 |
| CGGA_P128 | -0.159411442 | 0.475903483 | -0.635314925 | Primary | OA | WHO II | Male | 41 | 919 | 0 | 1 | 0 | Mutant | Non-codel | low | 1 |
| CGGA_P13 | -0.150896538 | 0.394353698 | -0.545250236 | Primary | OA | WHO II | Female | 40 | 1211 | 0 | 0 | 0 | Mutant | Codel | low | 1 |
| CGGA_P131 | -0.405711099 | 0.118388711 | -0.52409981 | Primary | AOA | WHO III | Female | 45 | 929 | 0 | 1 | 1 | Mutant | Codel | low | 1 |
| CGGA_P132 | -0.467826928 | 0.07767132 | -0.545498248 | Primary | OA | WHO II | Male | 42 | 936 | 0 | 1 | 0 | Mutant | Codel | low | 1 |
| CGGA_P136 | -0.556146997 | -0.086119346 | -0.470027651 | Primary | GBM | WHO IV | Female | 41 | 308 | 0 | 1 | 1 | Wildtype | NA | low | 0 |
| CGGA_P137 | -0.402228788 | -0.027190352 | -0.375038436 | Primary | A | WHO II | Male | 42 | 860 | 0 | 1 | 1 | Mutant | Non-codel | low | 1 |
| CGGA_P142 | -0.396356024 | 0.159761453 | -0.556117477 | Primary | AOA | WHO III | Male | 45 | 891 | 0 | 1 | 1 | Mutant | Codel | low | 1 |
| CGGA_P143 | -0.083305937 | -0.245522681 | 0.162216744 | Primary | GBM | WHO IV | Female | 66 | 261 | 1 | 1 | 1 | Wildtype | Non-codel | high | 0 |
| CGGA_P144 | -0.277093643 | 0.367080732 | -0.644174375 | Primary | AOA | WHO III | Male | 44 | 892 | 0 | 1 | 1 | Mutant | Non-codel | low | 1 |
| CGGA_P145 | -0.459775286 | 0.12718299 | -0.586958276 | Primary | OA | WHO II | Male | 53 | 308 | 1 | 1 | 0 | Mutant | Non-codel | low | 1 |
| CGGA_P146 | 0.10473882 | 0.343600092 | -0.238861272 | Recurrent | rA | WHO II | Male | 31 | 344 | 0 | 1 | 1 | Mutant | Non-codel | low | 1 |
| CGGA_P147 | 0.073423228 | 0.261685492 | -0.188262264 | Primary | OA | WHO II | Male | 52 | 863 | 1 | 1 | 0 | Mutant | Non-codel | low | 1 |
| CGGA_P15 | -0.634751167 | -0.352101452 | -0.282649715 | Primary | GBM | WHO IV | Male | 49 | 723 | 1 | 1 | 1 | NA | Non-codel | low | 0 |
| CGGA_P150 | -0.480670824 | -0.011531381 | -0.469139443 | Primary | OA | WHO II | Male | 34 | 349 | 0 | 1 | 0 | Mutant | Non-codel | low | 1 |
| CGGA_P151 | -0.225870536 | 0.159298004 | -0.38516854 | Primary | OA | WHO II | Female | 46 | 915 | 0 | 1 | 1 | Mutant | Non-codel | low | 1 |
| CGGA_P153 | -0.273628557 | 0.206761422 | -0.480389979 | Primary | A | WHO II | Male | 45 | 818 | 0 | 0 | 0 | Mutant | NA | low | 1 |
| CGGA_P154 | -0.350496087 | 0.136961476 | -0.487457563 | Primary | GBM | WHO IV | Male | 50 | NA | NA | NA | NA | Mutant | NA | low | 0 |
| CGGA_P155 | 0.147647324 | 0.491873307 | -0.344225983 | Primary | A | WHO II | Male | 50 | 817 | 0 | 1 | 0 | Wildtype | Non-codel | low | 1 |
| CGGA_P156 | -0.085371734 | 0.260306127 | -0.345677861 | Primary | O | WHO II | Male | 30 | 822 | 0 | 0 | 0 | Mutant | Codel | low | 1 |
| CGGA_P157 | 0.265534048 | 0.152577435 | 0.112956613 | Primary | AOA | WHO III | Female | 55 | 470 | 1 | 1 | 1 | Wildtype | Non-codel | high | 1 |
| CGGA_P158 | -0.246640152 | 0.133555026 | -0.380195178 | Primary | OA | WHO II | Male | 27 | 844 | 0 | 1 | 0 | Mutant | NA | low | 1 |
| CGGA_P159 | -0.529268466 | -0.082705335 | -0.446563131 | Primary | AOA | WHO III | Male | 59 | 211 | 1 | 0 | 0 | Wildtype | Non-codel | low | 1 |
| CGGA_P16 | 0.146006283 | 0.185927707 | -0.039921424 | Primary | GBM | WHO IV | Male | 38 | 447 | 0 | NA | NA | Mutant | Codel | high | 0 |
| CGGA_P160 | 0.231041914 | -0.064775301 | 0.295817215 | Primary | GBM | WHO IV | Female | 72 | 219 | 1 | 1 | 1 | Wildtype | Non-codel | high | 0 |
| CGGA_P163 | -0.063661114 | 0.037875308 | -0.101536422 | Recurrent | rAO | WHO III | Female | 45 | 880 | 0 | 1 | 1 | Mutant | Non-codel | high | 1 |
| CGGA_P164 | -0.504755163 | -0.201085443 | -0.30366972 | Primary | GBM | WHO IV | Male | 27 | 886 | 0 | 1 | 1 | Wildtype | NA | low | 0 |
| CGGA_P165 | -0.295554791 | 0.122433842 | -0.417988633 | Primary | AOA | WHO III | Female | 51 | 598 | 1 | 1 | 1 | Mutant | NA | low | 1 |
| CGGA_P17 | -0.157458189 | 0.350030885 | -0.507489074 | Primary | AOA | WHO III | Male | 30 | 1203 | 0 | 1 | 1 | Mutant | Codel | low | 1 |
| CGGA_P172 | -0.218178777 | 0.213856492 | -0.432035269 | Primary | AA | WHO III | Male | 33 | 803 | 0 | 1 | 1 | Mutant | NA | low | 1 |
| CGGA_P173 | -0.527426035 | 0.093971971 | -0.621398006 | Primary | OA | WHO II | Male | 31 | 782 | 0 | 1 | 0 | Mutant | Non-codel | low | 1 |
| CGGA_P174 | -0.092915221 | 0.401455154 | -0.494370375 | Primary | OA | WHO II | Male | 31 | 816 | 0 | 1 | 1 | Mutant | Codel | low | 1 |
| CGGA_P175 | -0.054512584 | 0.174897846 | -0.22941043 | Recurrent | rGBM | WHO IV | Male | 45 | 183 | 1 | 1 | 1 | Wildtype | NA | low | 0 |
| CGGA_P176 | -0.20204826 | 0.381836092 | -0.583884352 | Primary | OA | WHO II | Female | 32 | 804 | 0 | 1 | 0 | Mutant | Codel | low | 1 |
| CGGA_P177 | -0.431034184 | -0.001041991 | -0.429992193 | Primary | OA | WHO II | Male | 43 | 776 | 0 | 1 | 0 | Wildtype | Non-codel | low | 1 |
| CGGA_P178 | -0.14082096 | 0.182586706 | -0.323407666 | Primary | GBM | WHO IV | Female | 52 | 776 | 0 | 1 | 1 | Wildtype | NA | low | 0 |
| CGGA_P179 | -0.075658011 | 0.497780872 | -0.573438883 | Primary | OA | WHO II | Female | 53 | 778 | 0 | 1 | 1 | Mutant | Non-codel | low | 1 |
| CGGA_P18 | -0.461713555 | 0.026120662 | -0.487834217 | Primary | AA | WHO III | Male | 40 | 387 | 1 | 0 | 1 | Mutant | Non-codel | low | 1 |
| CGGA_P180 | -0.312834216 | -0.106032057 | -0.206802159 | Primary | GBM | WHO IV | Male | 47 | 260 | 1 | 1 | 1 | Mutant | NA | low | 0 |
| CGGA_P181 | -0.072072354 | 0.127534302 | -0.199606656 | Primary | AA | WHO III | Female | 21 | NA | NA | NA | NA | Wildtype | Non-codel | low | 1 |
| CGGA_P182 | 0.43790674 | 0.09282162 | 0.34508512 | Recurrent | rGBM | WHO IV | Female | 58 | 846 | 0 | 1 | 1 | Wildtype | Non-codel | high | 0 |
| CGGA_P183 | -0.316737525 | 0.106315056 | -0.423052581 | Primary | AO | WHO III | Male | 58 | 807 | 0 | 1 | 1 | Mutant | Codel | low | 1 |
| CGGA_P185 | 0.72399298 | 0.268082845 | 0.455910135 | Recurrent | rAA | WHO III | Female | 41 | 502 | 1 | 1 | 1 | Mutant | Non-codel | high | 1 |
| CGGA_P19 | -0.366216359 | 0.178449381 | -0.54466574 | Primary | OA | WHO II | Male | 39 | 1197 | 0 | 1 | 0 | Mutant | Non-codel | low | 1 |
| CGGA_P199 | 0.149269589 | -0.331848197 | 0.481117786 | Recurrent | rGBM | WHO IV | Male | 66 | 203 | 1 | 0 | 0 | Wildtype | Non-codel | high | 0 |
| CGGA_P20 | -0.330643002 | 0.1679769 | -0.498619902 | Primary | A | WHO II | Female | 58 | 1196 | 0 | 1 | 0 | Mutant | Non-codel | low | 1 |
| CGGA_P205 | -0.098747995 | 0.094857281 | -0.193605276 | Primary | GBM | WHO IV | Male | 66 | 302 | 1 | 1 | 1 | Wildtype | NA | low | 0 |
| CGGA_P21 | 0.131875376 | -0.331266355 | 0.463141731 | Primary | A | WHO II | Male | 25 | 1195 | 0 | 1 | 1 | Mutant | Non-codel | high | 1 |
| CGGA_P22 | 0.171783352 | -0.357560295 | 0.529343647 | Primary | GBM | WHO IV | Male | 62 | 406 | 1 | 1 | 1 | Wildtype | Non-codel | high | 0 |
| CGGA_P23 | -0.437540122 | -0.076364055 | -0.361176067 | Primary | AA | WHO III | Female | 62 | 392 | 1 | 1 | 1 | Wildtype | NA | low | 1 |
| CGGA_P25 | -0.294021395 | -0.102285352 | -0.191736043 | Primary | GBM | WHO IV | Male | 64 | 147 | 1 | 1 | 1 | Wildtype | NA | low | 0 |
| CGGA_P265 | -0.203565341 | 0.007150327 | -0.210715668 | Primary | AOA | WHO III | Male | 36 | 733 | 0 | 1 | 1 | Wildtype | NA | low | 1 |
| CGGA_P266 | 0.113654139 | 0.155474954 | -0.041820815 | Primary | AOA | WHO III | Female | 28 | 253 | 1 | 1 | 1 | Wildtype | NA | high | 1 |
| CGGA_P269 | 0.123259035 | -0.333293441 | 0.456552476 | Recurrent | rAO | WHO III | Female | 47 | 511 | 1 | 1 | 1 | Wildtype | Codel | high | 1 |
| CGGA_P27 | -0.02612607 | 0.33050288 | -0.35662895 | Primary | OA | WHO II | Female | 37 | 413 | 0 | 1 | 0 | Mutant | Non-codel | low | 1 |
| CGGA_P270 | -0.241759886 | 0.146737876 | -0.388497762 | Recurrent | rA | WHO II | Male | 33 | 322 | 1 | 1 | 1 | Mutant | Non-codel | low | 1 |
| CGGA_P271 | -0.431123088 | 0.007427017 | -0.438550105 | Primary | A | WHO II | Male | 42 | 710 | 0 | 1 | 0 | Mutant | NA | low | 1 |
| CGGA_P279 | 0.318971078 | -0.095375958 | 0.414347036 | Recurrent | rAO | WHO III | Female | 47 | 668 | 0 | 1 | 1 | Mutant | Codel | high | 1 |
| CGGA_P28 | 0.435888271 | 0.094609429 | 0.341278842 | Primary | GBM | WHO IV | Male | 61 | 107 | 1 | 0 | 0 | Wildtype | Non-codel | high | 0 |
| CGGA_P280 | -0.183420919 | 0.270984577 | -0.454405496 | Recurrent | rGBM | WHO IV | Female | 52 | 675 | 0 | 1 | 1 | Wildtype | Non-codel | low | 0 |
| CGGA_P283 | 0.366298214 | -0.270431174 | 0.636729388 | Recurrent | rGBM | WHO IV | Male | 38 | 664 | 0 | 1 | 1 | Wildtype | Non-codel | high | 0 |
| CGGA_P286 | -0.086029024 | -0.269686598 | 0.183657574 | Recurrent | rOA | WHO II | Female | 57 | 658 | 0 | NA | NA | Mutant | Codel | high | 1 |
| CGGA_P29 | 0.017635144 | 0.218966967 | -0.201331823 | Recurrent | rAOA | WHO III | Female | 22 | 103 | 1 | 0 | 1 | Mutant | NA | low | 1 |
| CGGA_P295 | -0.312890561 | -0.1891778 | -0.123712761 | Recurrent | rGBM | WHO IV | Male | 46 | 273 | 1 | 1 | 1 | Wildtype | Non-codel | low | 0 |
| CGGA_P298 | 0.297106852 | -0.161355713 | 0.458462565 | Recurrent | rAO | WHO III | Female | 50 | 634 | 0 | 1 | 0 | Mutant | Codel | high | 1 |
| CGGA_P3 | -0.163231908 | -0.008116411 | -0.155115497 | Recurrent | rAOA | WHO III | Male | 32 | 1399 | 0 | 1 | 0 | Mutant | NA | low | 1 |
| CGGA_P30 | 0.269259022 | -0.30984403 | 0.579103052 | Primary | A | WHO II | Male | 48 | 404 | 1 | 1 | 1 | Wildtype | Non-codel | high | 1 |
| CGGA_P306 | 0.239283076 | 0.123556235 | 0.115726841 | Recurrent | rO | WHO II | Male | 49 | 592 | 0 | 1 | NA | Mutant | Codel | high | 1 |
| CGGA_P308 | 0.022521829 | 0.186402828 | -0.163880999 | Recurrent | rAA | WHO III | Male | 38 | 563 | 1 | 1 | 1 | Mutant | Non-codel | low | 1 |
| CGGA_P31 | -0.254268144 | 0.239817504 | -0.494085648 | Primary | A | WHO II | Male | 42 | 1205 | 0 | 0 | 1 | Wildtype | Non-codel | low | 1 |
| CGGA_P310 | -0.47552626 | -0.282556346 | -0.192969914 | Recurrent | rAA | WHO III | Female | 47 | 224 | 1 | NA | NA | NA | Codel | low | 1 |
| CGGA_P311 | -0.520963347 | -0.566933599 | 0.045970252 | Recurrent | rA | WHO II | Male | 36 | 581 | 0 | NA | NA | Mutant | Non-codel | high | 1 |
| CGGA_P314 | 0.113963364 | -0.184978749 | 0.298942113 | Recurrent | rAO | WHO III | Female | 34 | 572 | 0 | 0 | 0 | Mutant | Codel | high | 1 |
| CGGA_P315 | -0.240739931 | -0.194185495 | -0.046554436 | Recurrent | rOA | WHO II | Female | 47 | 451 | 1 | 1 | 0 | Mutant | Non-codel | high | 1 |
| CGGA_P316 | -0.444504004 | -0.138253722 | -0.306250282 | Recurrent | rA | WHO II | Male | 44 | 567 | 0 | 1 | 0 | NA | Non-codel | low | 1 |
| CGGA_P319 | 0.759293969 | 0.049234332 | 0.710059637 | Recurrent | rAOA | WHO III | Male | 37 | 565 | 0 | 1 | NA | Mutant | Non-codel | high | 1 |
| CGGA_P326 | -0.353595358 | -0.270328209 | -0.083267149 | Recurrent | rAA | WHO III | Female | 48 | 551 | 0 | 0 | 0 | Mutant | Non-codel | high | 1 |
| CGGA_P328 | -0.067206733 | -0.210207737 | 0.143001004 | Recurrent | rAA | WHO III | Female | 54 | 546 | 0 | 1 | NA | Mutant | Non-codel | high | 1 |
| CGGA_P335 | 0.398808746 | -0.212887855 | 0.611696601 | Recurrent | rGBM | WHO IV | Male | 42 | 537 | 0 | NA | NA | Mutant | Codel | high | 0 |
| CGGA_P337 | 0.610861679 | 0.381788791 | 0.229072888 | Recurrent | rAO | WHO III | Female | 61 | 532 | 0 | NA | NA | Mutant | Codel | high | 1 |
| CGGA_P338 | 0.128990225 | 0.449759028 | -0.320768803 | Recurrent | rO | WHO II | Male | 51 | 532 | 0 | 1 | NA | Wildtype | Non-codel | low | 1 |
| CGGA_P346 | -0.028732436 | -0.518215575 | 0.489483139 | Recurrent | rOA | WHO II | Male | 42 | 522 | 0 | NA | NA | Mutant | Non-codel | high | 1 |
| CGGA_P356 | 0.3291504 | -0.118654794 | 0.447805194 | Recurrent | rOA | WHO II | Female | 22 | 506 | 0 | NA | NA | Mutant | Non-codel | high | 1 |
| CGGA_P358 | -0.334975495 | 0.069493873 | -0.404469368 | Recurrent | rOA | WHO II | Male | 44 | 504 | 0 | NA | 1 | Mutant | Non-codel | low | 1 |
| CGGA_P364 | 0.315536306 | -0.395597702 | 0.711134008 | Recurrent | rAA | WHO III | Female | 41 | 495 | 1 | 1 | NA | Mutant | Non-codel | high | 1 |
| CGGA_P385 | -0.203410076 | 0.350261594 | -0.55367167 | Recurrent | rGBM | WHO IV | Male | 22 | 449 | 0 | NA | NA | Wildtype | Non-codel | low | 0 |
| CGGA_P388 | -0.598443316 | -0.373681421 | -0.224761895 | Recurrent | rAA | WHO III | Female | 37 | 293 | 1 | NA | NA | Mutant | Non-codel | low | 1 |
| CGGA_P392 | 0.60122796 | 0.495681827 | 0.105546133 | Recurrent | rA | WHO II | Male | 35 | 437 | 0 | 0 | 1 | Mutant | Codel | high | 1 |
| CGGA_P399 | -0.029675906 | 0.078030473 | -0.107706379 | Recurrent | rOA | WHO II | Female | 41 | 428 | 0 | NA | NA | Mutant | Codel | low | 1 |
| CGGA_P401 | 0.55607248 | 0.026579162 | 0.529493318 | Recurrent | rGBM | WHO IV | Male | 31 | 543 | 0 | NA | NA | Mutant | Non-codel | high | 0 |
| CGGA_P411 | 0.809133161 | 0.142331767 | 0.666801394 | Recurrent | rGBM | WHO IV | Female | 47 | 411 | 0 | 1 | 1 | Mutant | Non-codel | high | 0 |
| CGGA_P415 | -0.482315771 | -0.229485966 | -0.252829805 | Recurrent | rGBM | WHO IV | Female | 56 | 195 | 1 | NA | NA | Wildtype | Non-codel | low | 0 |
| CGGA_P416 | 0.663407249 | 0.402650682 | 0.260756567 | Recurrent | rAO | WHO III | Male | 29 | 408 | 0 | NA | NA | Mutant | Codel | high | 1 |
| CGGA_P421 | 0.574245037 | -0.141675241 | 0.715920278 | Recurrent | rAA | WHO III | Male | 26 | 308 | 1 | NA | NA | Mutant | Non-codel | high | 1 |
| CGGA_P422 | 0.207045859 | 0.380760232 | -0.173714373 | Primary | OA | WHO II | Female | 41 | 430 | 0 | NA | NA | Mutant | Codel | low | 1 |
| CGGA_P437 | 0.735785153 | 0.349627459 | 0.386157694 | Primary | AO | WHO III | Female | 47 | 356 | 0 | 1 | NA | Mutant | Codel | high | 1 |
| CGGA_P438 | 0.762045534 | 0.538347599 | 0.223697935 | Recurrent | rAO | WHO III | Male | 41 | 310 | 0 | NA | NA | Mutant | Codel | high | 1 |
| CGGA_P439 | 0.427763442 | 0.041444101 | 0.386319341 | Recurrent | rA | WHO II | Male | 45 | 309 | 0 | 1 | 1 | Mutant | Non-codel | high | 1 |
| CGGA_P446 | 0.583040087 | 0.200728294 | 0.382311793 | Recurrent | rAO | WHO III | Female | 47 | NA | NA | 1 | 1 | Mutant | Codel | high | 1 |
| CGGA_P461 | 1.090209633 | 0.528503801 | 0.561705832 | Recurrent | rAOA | WHO III | Male | 28 | NA | NA | NA | NA | Mutant | Non-codel | high | 1 |
| CGGA_P468 | 0.612298421 | 0.103911874 | 0.508386547 | Primary | AO | WHO III | Female | 42 | NA | NA | NA | NA | Mutant | Codel | high | 1 |
| CGGA_P483 | 1.03512091 | 0.406529723 | 0.628591187 | Recurrent | rAOA | WHO III | Male | 41 | NA | NA | NA | NA | Wildtype | Non-codel | high | 1 |
| CGGA_P492 | 0.337121725 | 0.438021132 | -0.100899407 | Recurrent | rOA | WHO II | Female | 44 | NA | NA | NA | NA | Mutant | Codel | high | 1 |
| CGGA_P499 | 0.141247899 | 0.108788973 | 0.032458926 | Recurrent | rGBM | WHO IV | Female | 69 | NA | NA | NA | NA | Wildtype | Non-codel | high | 0 |
| CGGA_P5 | -0.410666123 | -0.006389408 | -0.404276715 | Recurrent | rAA | WHO III | Female | 45 | 378 | 1 | 1 | 1 | Mutant | Non-codel | low | 1 |
| CGGA_P500 | 0.047026385 | 0.268297861 | -0.221271476 | Recurrent | rO | WHO II | Female | 34 | NA | NA | 1 | 1 | Mutant | Codel | low | 1 |
| CGGA_P501 | 0.381994953 | 0.09893201 | 0.283062943 | Recurrent | rOA | WHO II | Male | 27 | NA | NA | NA | NA | Mutant | Non-codel | high | 1 |
| CGGA_P505 | 0.026433406 | 0.161940613 | -0.135507207 | Recurrent | rOA | WHO II | Male | 34 | NA | NA | NA | NA | Mutant | Non-codel | low | 1 |
| CGGA_P508 | 0.716097272 | 0.267965813 | 0.448131459 | Recurrent | rAOA | WHO III | Male | 40 | NA | NA | NA | NA | Mutant | Non-codel | high | 1 |
| CGGA_P510 | 0.41692117 | 0.202490862 | 0.214430308 | Recurrent | rAO | WHO III | Male | 42 | NA | NA | NA | NA | Mutant | Non-codel | high | 1 |
| CGGA_P512 | 0.475908711 | -0.179755101 | 0.655663812 | Recurrent | rGBM | WHO IV | Male | 23 | NA | NA | NA | NA | Wildtype | Non-codel | high | 0 |
| CGGA_P520 | 0.384745036 | 0.032339283 | 0.352405753 | Recurrent | rAO | WHO III | Female | 44 | NA | NA | NA | NA | Mutant | Codel | high | 1 |
| CGGA_P568 | 0.82748904 | 0.217297646 | 0.610191394 | Recurrent | rAOA | WHO III | Female | 23 | NA | NA | 0 | 0 | Mutant | Codel | high | 1 |
| CGGA_P585 | 0.801941853 | 0.182805342 | 0.619136511 | Recurrent | rGBM | WHO IV | Male | 50 | NA | NA | NA | NA | Wildtype | Non-codel | high | 0 |
| CGGA_P594 | 0.491231504 | 0.007370642 | 0.483860862 | Recurrent | rAOA | WHO III | Male | 40 | NA | NA | NA | NA | Wildtype | Non-codel | high | 1 |
| CGGA_P596 | 0.231691603 | -0.341964171 | 0.573655774 | Recurrent | rGBM | WHO IV | Female | 60 | NA | NA | NA | NA | Mutant | Codel | high | 0 |
| CGGA_P604 | 0.867086183 | 0.292226741 | 0.574859442 | Recurrent | rAOA | WHO III | Female | 15 | NA | NA | 0 | 0 | Wildtype | Non-codel | high | 1 |
| CGGA_P609 | 0.564768291 | -0.04943363 | 0.614201921 | Primary | GBM | WHO IV | Female | 19 | NA | NA | 0 | 0 | Wildtype | Non-codel | high | 0 |
| CGGA_P610 | -0.061457675 | 0.043087149 | -0.104544824 | Recurrent | rGBM | WHO IV | Male | 62 | NA | NA | 0 | 0 | Wildtype | NA | high | 0 |
| CGGA_P615 | 0.171747113 | 0.511124788 | -0.339377675 | Recurrent | rOA | WHO II | Male | 40 | NA | NA | 0 | 0 | Mutant | Codel | low | 1 |
| CGGA_P619 | 0.191253906 | 0.034590407 | 0.156663499 | Recurrent | rGBM | WHO IV | Female | 66 | NA | NA | NA | NA | Wildtype | Non-codel | high | 0 |
| CGGA_P623 | 0.13986705 | -0.138923337 | 0.278790387 | Primary | AOA | WHO III | Male | 52 | NA | NA | NA | NA | Mutant | Non-codel | high | 1 |
| CGGA_P625 | 0.749937976 | 0.164604194 | 0.585333782 | Recurrent | rGBM | WHO IV | Female | 46 | NA | NA | NA | NA | Mutant | Non-codel | high | 0 |
| CGGA_P633 | 0.755394674 | 0.377893649 | 0.377501025 | Recurrent | rAOA | WHO III | Female | 40 | NA | NA | 1 | 1 | Mutant | Non-codel | high | 1 |
| CGGA_P7 | -0.021731338 | -0.138824288 | 0.11709295 | Recurrent | rGBM | WHO IV | Male | 45 | 110 | 1 | 1 | 0 | Wildtype | Non-codel | high | 0 |
| CGGA_P83 | -0.179027751 | -0.370080956 | 0.191053205 | Primary | A | WHO II | Female | 28 | 1030 | 0 | 1 | 1 | Mutant | Non-codel | high | 1 |
| CGGA_P84 | -0.549465685 | 0.018754823 | -0.568220508 | Primary | A | WHO II | Female | 40 | 1026 | 0 | 1 | 0 | Mutant | Non-codel | low | 1 |
| CGGA_P86 | -0.067509748 | 0.260827689 | -0.328337437 | Primary | O | WHO II | Female | 34 | 1026 | 0 | 1 | 0 | Mutant | Codel | low | 1 |
| CGGA_P87 | -0.179218553 | -0.201736555 | 0.022518002 | Recurrent | rGBM | WHO IV | Male | 32 | 240 | 1 | 1 | 1 | Mutant | Non-codel | high | 0 |
| CGGA_P89 | -0.203920927 | -0.09492827 | -0.108992657 | Recurrent | rGBM | WHO IV | Male | 42 | 294 | 1 | 1 | 1 | Wildtype | Non-codel | low | 0 |
| CGGA_P93 | -0.293401195 | 0.243262763 | -0.536663958 | Primary | A | WHO II | Female | 45 | 1010 | 0 | 0 | 0 | Mutant | Non-codel | low | 1 |
| CGGA_P98 | -0.247698008 | 0.18540368 | -0.433101688 | Recurrent | rAOA | WHO III | Male | 44 | 317 | 1 | 1 | 1 | Mutant | Non-codel | low | 1 |
| CGGA_P99 | 0.043940534 | -0.304764497 | 0.348705031 | Recurrent | rGBM | WHO IV | Male | 52 | 679 | 0 | 1 | 1 | Mutant | Non-codel | high | 0 |
