# Supplementary material for: Differential regulation of H3K9/H3K14 acetylation by small molecules drives neuron-fate-induction of glioma cell
Source: Cell Death Dis. 2023 Feb 20;14(2):142. doi: 10.1038/s41419-023-05611-8 (PMC9941105; doi:10.1038/s41419-023-05611-8)
Supplement: Supplementary file 8 — Original Data File [file 41419_2023_5611_MOESM8_ESM.docx]

**Fig 1F**

45

75

**
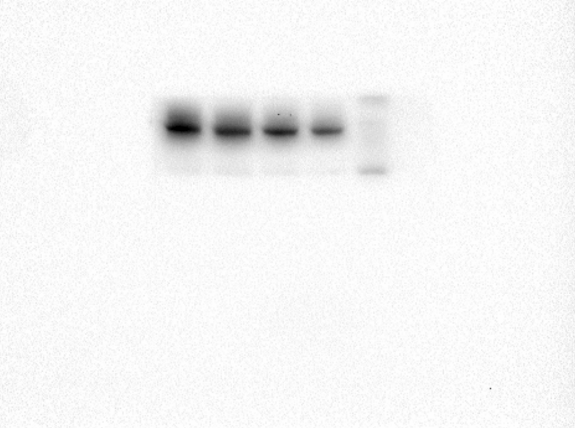

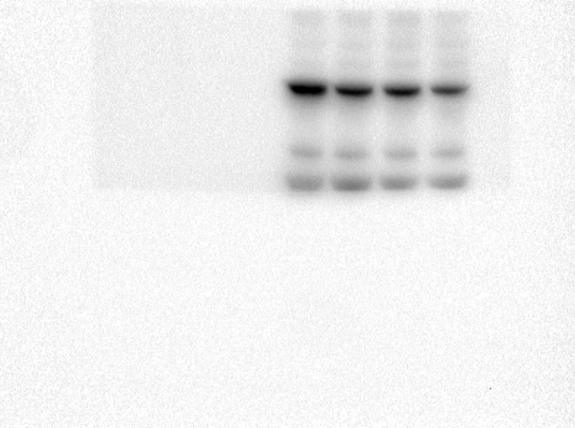
**

75

100

**
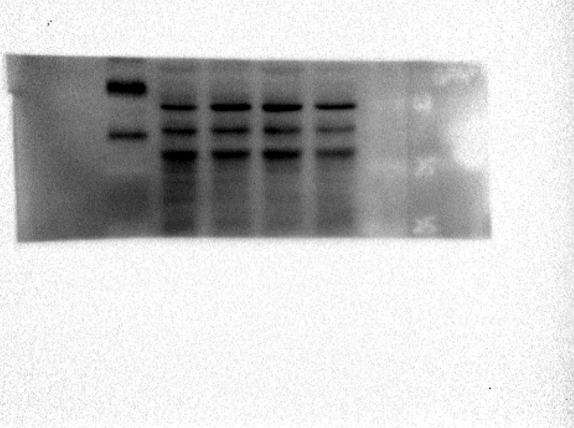

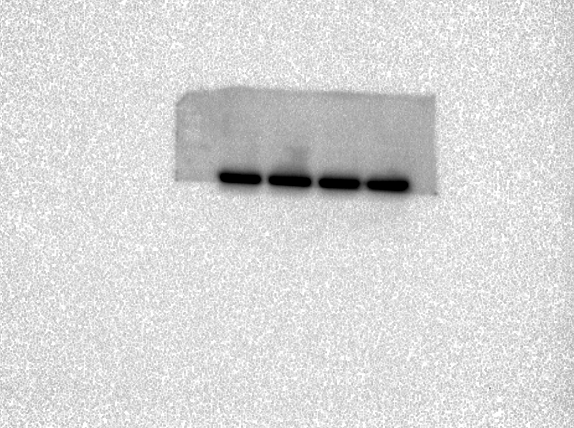

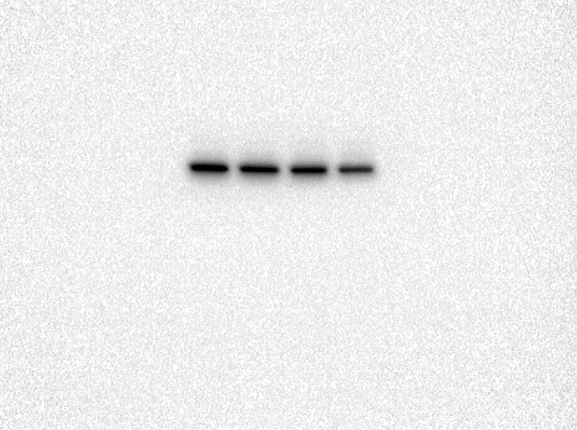
**

GFAP

PCNA

Tublin

MAP2

TUBB3

60

75

25

45

45

35

25

**Fig S4A
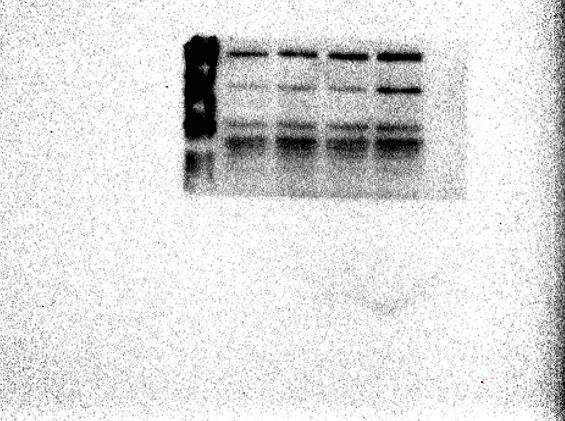

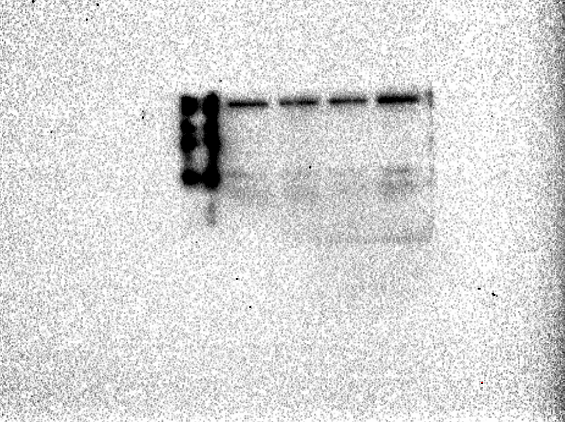
**

**
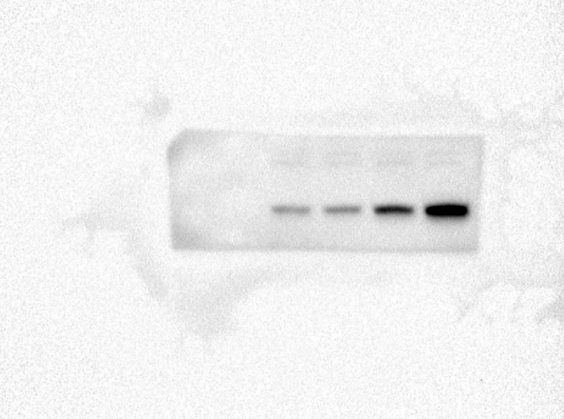

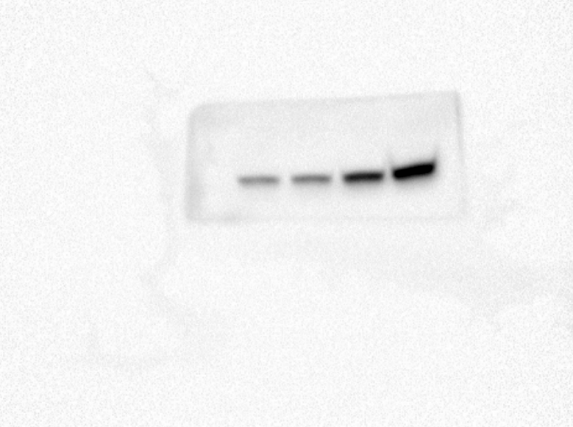

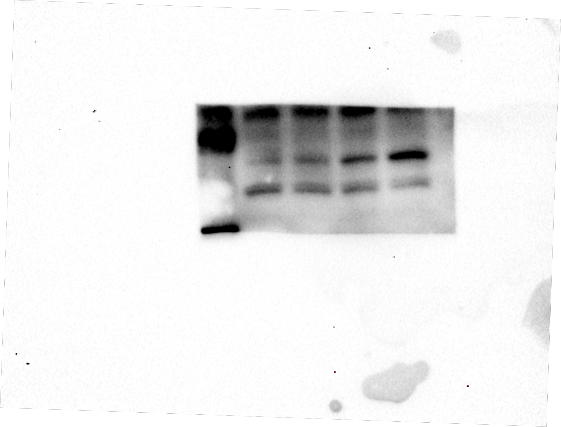

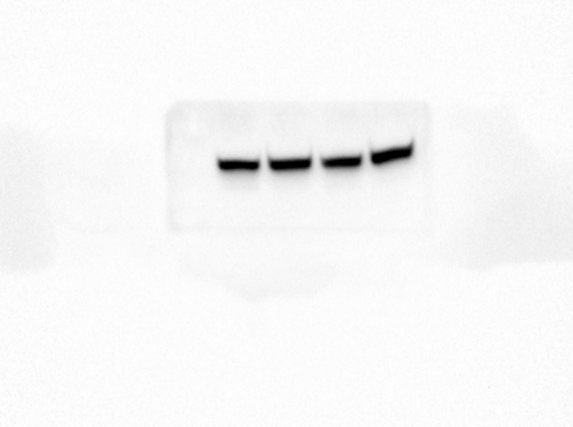
**

H3K14ac

H3K9ac

H3

H3ac

MAP2

TUBB3

25

10

15

25

10

15

25

10

15

25

10

15

60

75

25

45

**Fig S5E**


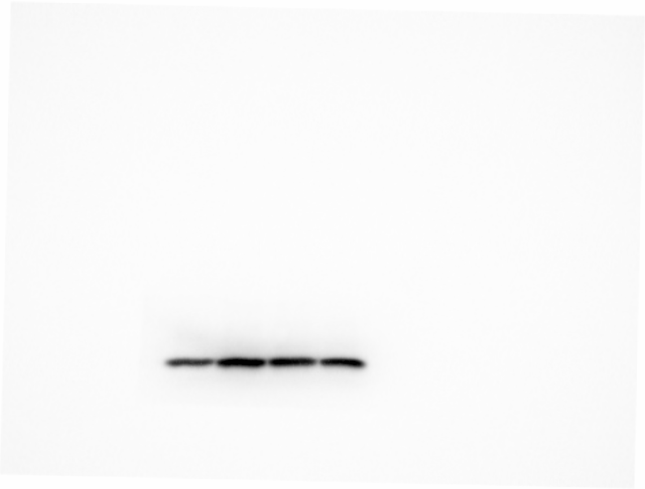

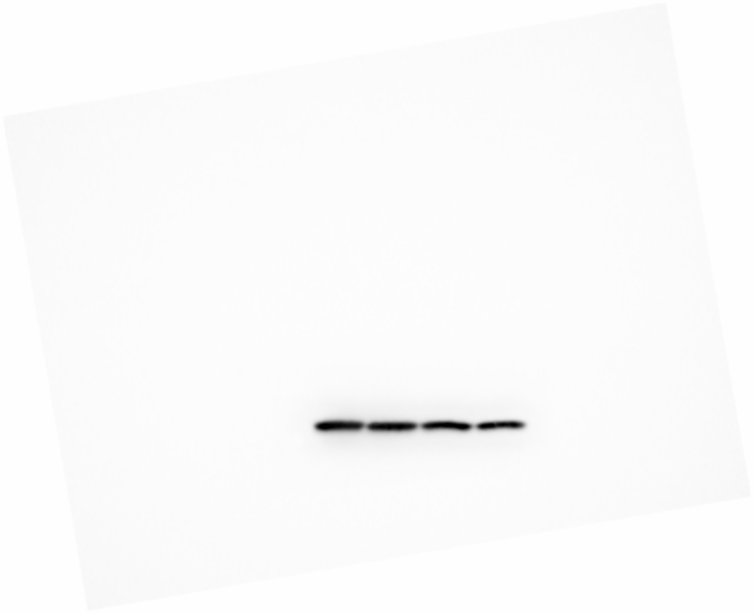

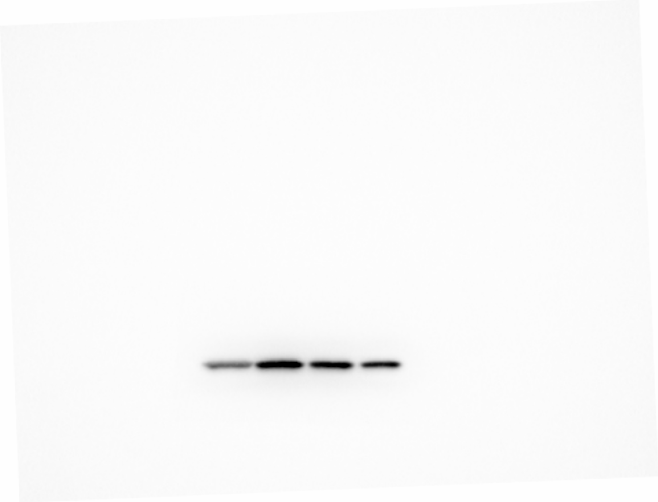


H3

H3K14ac

H3K9ac

25

15

10

25

15

10

25

15

10

**Fig 6D**


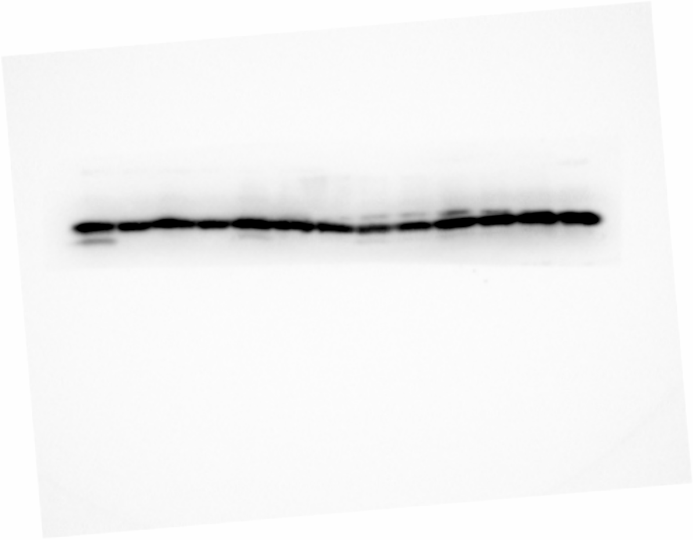

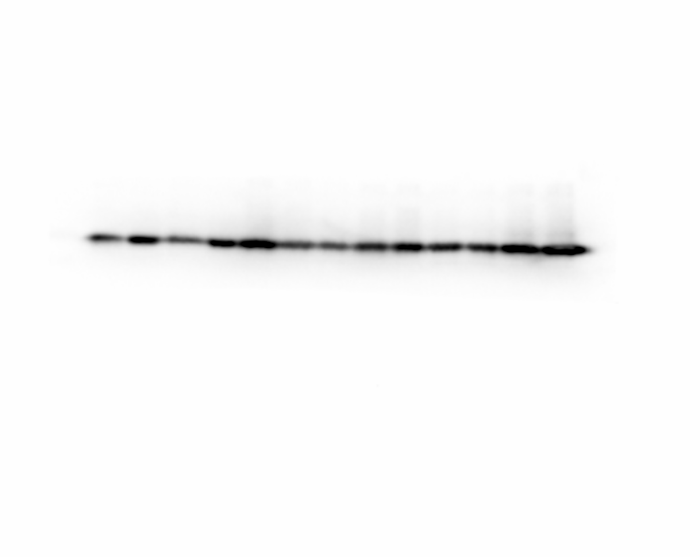

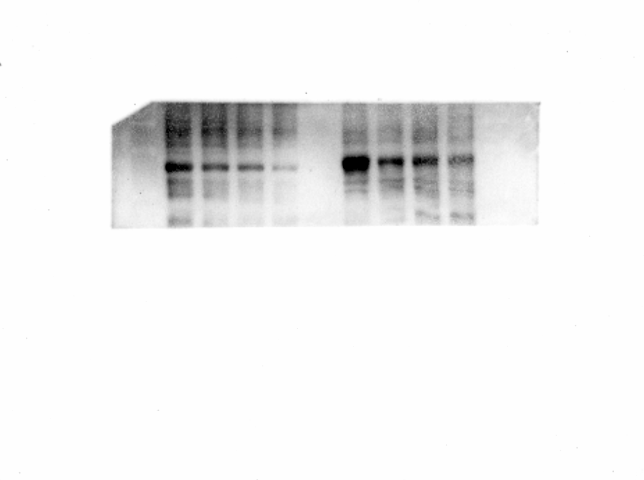

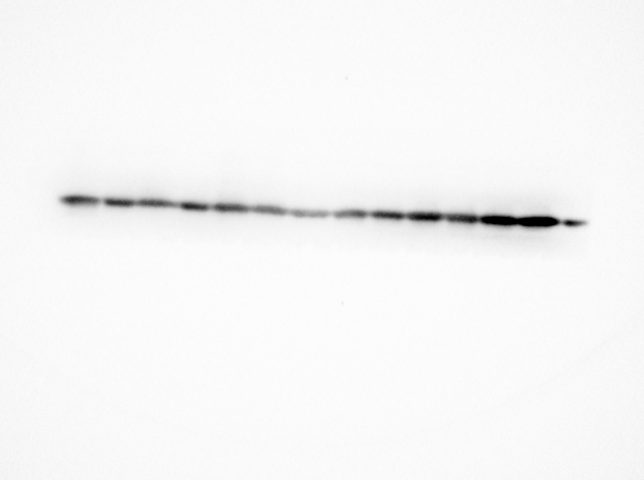


H3

CD133

H3K14ac

H3K9ac

GSC-11

GSC-1

GSC-11

GSC-1

GSC-11

GSC-1

GSC-11

GSC-1

180

75

25

15

10

25

15

10

25

15

10
